# Smoking, DNA Methylation, and Breast Cancer: A Mendelian Randomization Study

**DOI:** 10.3389/fonc.2021.745918

**Published:** 2021-09-28

**Authors:** Haibo Tang, Desong Yang, Chaofei Han, Ping Mu

**Affiliations:** ^1^ Department of Metabolic and Bariatric Surgery, The Third Xiangya Hospital, Central South University, Changsha, China; ^2^ Department of Thoracic Surgery II, Hunan Cancer Hospital and The Affiliated Cancer Hospital of Xiangya School of Medicine, Central South University, Changsha, China; ^3^ Department of Burn and Plastic Surgery, The Third Xiangya Hospital, Central South University, Changsha, China; ^4^ Department of Physiology, Shenyang Medical College, Shenyang, China

**Keywords:** Mendelian randomization (MR), smoking, DNA methylation, breast cancer, causal inference

## Abstract

**Background:**

Smoking was strongly associated with breast cancer in previous studies. Whether smoking promotes breast cancer through DNA methylation remains unknown.

**Methods:**

Two-sample Mendelian randomization (MR) analyses were conducted to assess the causal effect of smoking-related DNA methylation on breast cancer risk. We used 436 smoking-related CpG sites extracted from 846 middle-aged women in the ARIES project as exposure data. We collected summary data of breast cancer from one of the largest meta-analyses, including 69,501 cases for ER+ breast cancer and 21,468 cases for ER− breast cancer. A total of 485 single-nucleotide polymorphisms (SNPs) were selected as instrumental variables (IVs) for smoking-related DNA methylation. We further performed an MR Steiger test to estimate the likely direction of causal estimate between DNA methylation and breast cancer. We also conducted colocalization analysis to evaluate whether smoking-related CpG sites shared a common genetic causal SNP with breast cancer in a given region.

**Results:**

We established four significant associations after multiple testing correction: the CpG sites of cg2583948 [OR = 0.94, 95% CI (0.91–0.97)], cg0760265 [OR = 1.07, 95% CI (1.03–1.11)], cg0420946 [OR = 0.95, 95% CI (0.93–0.98)], and cg2037583 [OR =1.09, 95% CI (1.04–1.15)] were associated with the risk of ER+ breast cancer. All the four smoking-related CpG sites had a larger variance than that in ER+ breast cancer (all *p* < 1.83 × 10^−11^) in the MR Steiger test. Further colocalization analysis showed that there was strong evidence (based on PPH4 > 0.8) supporting a common genetic causal SNP between the CpG site of cg2583948 [with *IMP3* expression (PPH4 = 0.958)] and ER+ breast cancer. There were no causal associations between smoking-related DNA methylation and ER− breast cancer.

**Conclusions:**

These findings highlight potential targets for the prevention of ER+ breast cancer. Tissue-specific epigenetic data are required to confirm these results.

## Introduction

In the latest global cancer data released by the International Agency for Research on Cancer (IARC), breast cancer has been confirmed as the most commonly diagnosed cancer in women (https://www.iarc.who.int/). The data indicated that one in every four cancer cases diagnosed is breast cancer, and one in every six cancer deaths is breast cancer in women in 2020. Breast cancer caused 685,000 deaths in 2020, and it became the fifth leading cause of cancer mortality worldwide. It has been confirmed that cigarette smoking, one of the most important environmental risk factors, represents a significant effect on breast cancer risk (1–4).

Cigarette smoking was reported to induce reactive oxygen species (ROS), oxidative stress, and DNA methylation, which play vital roles in carcinogenesis (5, 6). The changes in DNA methylation profiles have been detected between smokers and nonsmokers in various cancers (6–8), and the effect of cigarette smoking on the DNA methylation patterns of breast tumors has also been revealed by using a cancer-focused array (9), whereas cancer-focused array only provides the correlation analysis, which could not avoid the interference of confounding factors and reverse causality. In contrast to previous studies with the above limitations, Mendelian randomization (MR) offers an opportunity to efficiently and reliably assess the causal effects between smoking-related DNA methylation patterns and breast cancer risk.

MR is considered as “nature’s randomized control trial” (10), using genetic variants significantly associated with the exposure of interest to explore causal effects on the outcomes (11), which is similar to random different interventions in randomized controlled trials (RCTs). We performed a two-sample MR study to evaluate the effect magnitude and direction of smoking-related methylation on the risk of breast cancer.

## Methods

### Study Design

**Figure 1:**
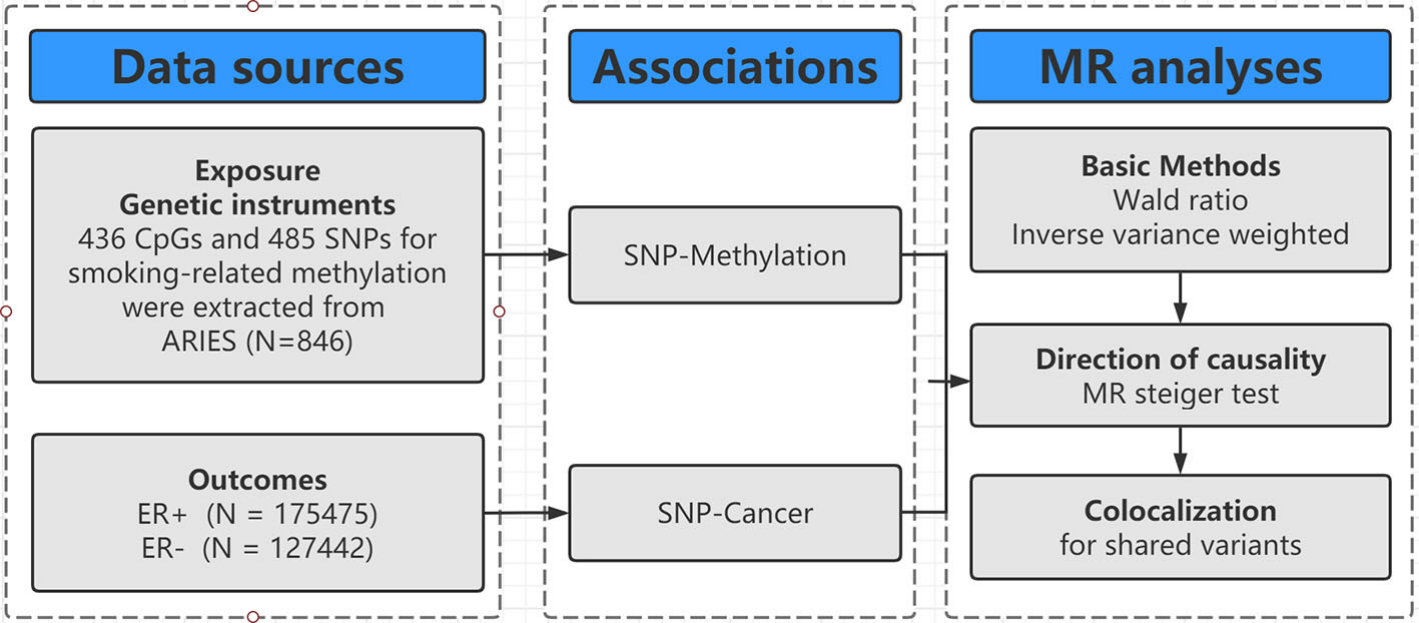
Flowchart of Mendelian randomization framework in this study. SNP indicates single-nucleotide polymorphism; ER, estrogen receptor; MR, mendelian randomization.

The design of our study is shown in **Figure 1**. Firstly, we identified genetic variants as IVs for smoking-related methylation. Secondly, we collected the complete summary data from the large-scale genome-wide association studies (GWASs) for breast cancer. Thirdly, we performed a two-sample MR with two basic MR methods [e.g., Wald ratio for only one SNP and inverse-variance weighted (IVW) for two SNPs]. Fourthly, we conducted an MR Steiger test and a colocalization to evaluate the causal direction and identify the shared SNP between DNA methylation and breast cancer.

### Data for Exposure

We collected summary data of smoking-related DNA methylation from the ARIES (Accessible Resource for Integrated Epigenomics Studies) project, which used the Illumina Infinium HumanMethylation450 (HM450) BeadChip to generate epigenetic data on cord blood and peripheral blood samples from 1,018 mother–offspring pairs at five time points (birth, childhood, adolescence, antenatal period, and middle age) (12). A recent study considered those methylation quantitative trait loci (mQTLs) identified in the middle-age time point among women in ARIES (mean age = 47.5 years, *n* = 846) and found 474 smoking-related CpG sites proxied by at least one mQTL (96% for cis, 4% for trans). Of these, 406 CpGs (86%) were proxied by a single SNP (13). On this basis, we further screened valid IVs according to the following criteria: (1) we selected SNPs as IVs using a *p*-value threshold of 5 × 10^−8^ (IV assumption 1, **Supplementary Figure S1**); (2) we included SNPs that have definitive allele information especially effect allele and its frequency; and (3) only cis-mQTLs (referring to genetic variants that act on local genes) were included in this study because trans-mQTLs (referring to genetic variants that act on distant genes and genes locating at different chromosomes) may lead to significant bias through horizontal pleiotropy. *F* statistic represents the strength of the relationship between IVs and VAT. Generally, *F* > 10 may attenuate bias produced by weak IVs (14).

### Data for Outcomes

We collected summary data of breast cancer from the largest available GWAS summary statistics to date, a meta-analysis of 67 studies including 69,501 ER+ and 21,648 ER− cases, and 105,974 controls for breast cancer (15). Of these, most studies were population-based case–control studies, or case–control studies nested within population-based cohorts. All studies provided the participants’ status and age at diagnosis, and the majority provided additional clinical data and lifestyle factors, which have been curated and incorporated into the Breast Cancer Association Consortium (BCAC). All participating studies were approved by their respective ethics review board, and all subjects provided informed consent.

### Statistical Analysis

#### Two-Sample Mendelian Randomization

As shown in **Supplementary Figure S1**, we calculated the effect of every single SNP using the basic model: β_causal effect_ = β_ZY_/β_ZX_ (β_ZX_ and β_ZY_ represent the regression coefficient on smoking-related DNA methylation and breast cancer, respectively). Generally, a valid instrument should satisfy three assumptions (**Supplementary Figure S1**): (1) must be truly associated with smoking-related methylation passing genome-wide significance (*p* < 5 × 10^−8^); (2) not associated with confounders of DNA methylation or breast cancer; and (3) should only be associated to the breast cancer through the smoking-related DNA methylation.

To evaluate the causal effects of smoking-related DNA methylation on the risk of breast cancer, we conducted a two-sample MR analysis (16) using two MR methods, including Wald ratio for only one SNP and IVW (17). The IVW is a conventional method to obtain an MR estimate performing a meta-analysis of each Wald ratio for multiple SNPs. The IVW could provide the strongest statistical power when none of the assumptions are violated.

#### Sensitivity Analyses

It is possible that smoking-related DNA methylation may have a causal effect on breast cancer. Another possibility concerns reverse causation, whereby SNPs used as proxies for DNA methylation have their primary effect through breast cancer rather than through DNA methylation. For this, we performed the MR Steiger test (18) to estimate the likely direction of effect between DNA methylation and breast cancer.

In addition, for smoking-related DNA methylation (CpG sites) where there was evidence supporting a causal relationship on breast cancer, we applied colocalization analysis to investigate whether the SNP responsible for influencing smoking-related DNA methylation at each CpG site was the same SNP influencing changes to breast cancer risk. As recommended by the developers of the colocalization, a posterior probability of hypothesis (PPH, exposure, and outcome are associated and share a single causal SNP) of 80% or higher was considered evidence of colocalization.

In our study, most CpG sites were proxied by a single SNP, so it was difficult to evaluate horizontal pleiotropy using conventional methods like evaluation of MR-Egger regression intercept. We conducted a phenome-wide association test (19) to assess the relationships of IVs with potential confounders of breast cancer such as body mass index, age at menarche or menopause, and alcohol usage. We also performed a Cochran’s Q-test for CpG sites proxied by at least two SNPs (20).

Based on the analyses as mentioned above, we could conclusively establish a robustly causal association when satisfying the following conditions: (1) the results of Wald ratio or IVW reached the multiple comparisons adjusted *p*-value (*q*-value after false discovery rate) < 0.05; (2) the MR Steiger test showed a direction of effect from smoking-related DNA methylation to breast cancer; and (3) colocalization analysis supported the idea that smoking-related DNA methylation and breast cancer shared a common genetic causal SNP in a given region.

MR analyses and the MR Steiger test were performed in R (version 4.0.3) with the R package “TwoSampleMR” (21), and colocalization analysis was performed using the R package “Coloc” (22). The false discovery rate was calculated by the R package “fdrtool”. The *p*-values were two-sided, and the statistical significance was set at the level of adjusted *p*-value < 0.05 after false discovery rate correction.

## Results

### Participant Characteristics and Instruments

The characteristics of the participants from the ARIES project for smoking-related DNA methylation and meta-analysis for breast cancer are shown in **Table 1**. After screening, 485 SNPs were obtained for 436 CpG sites (all cis-mQTLs) (**Supplementary Table S1**), with *F* statistics ranging from 19 to 952, reflecting a strong instrument strength for smoking-related DNA methylation. All the participants had an identical genetic background (all Europeans), as a consistent selection in exposure data, and to our knowledge, there was no sample overlap between the exposure and outcome GWASs.

**Table 1:** Characteristics of smoking-related methylation datasets and breast cancer.

Exposure	Data source	CpG	*F*	Cases/Controls	Sample size	Population
Smoking-related methylation	ARIES	436	19–952	NA	846	European
**Outcomes**	**Consortium**	**Studies**		**Cases/Controls**	**Sample size**	**Population**
ER+ breast cancer	BCAC	67		69,501/105,974	175,475	European
ER− breast cancer	BCAC	67		21,468/105,974	127,442	European

ARIES indicates Accessible Resource for Integrated Epigenomics Studies; ER, estrogen receptor; BCAC, Breast Cancer Association Consortium; NA, not applicable.

### Main Results of Two-Sample MR

**Figure 2:**
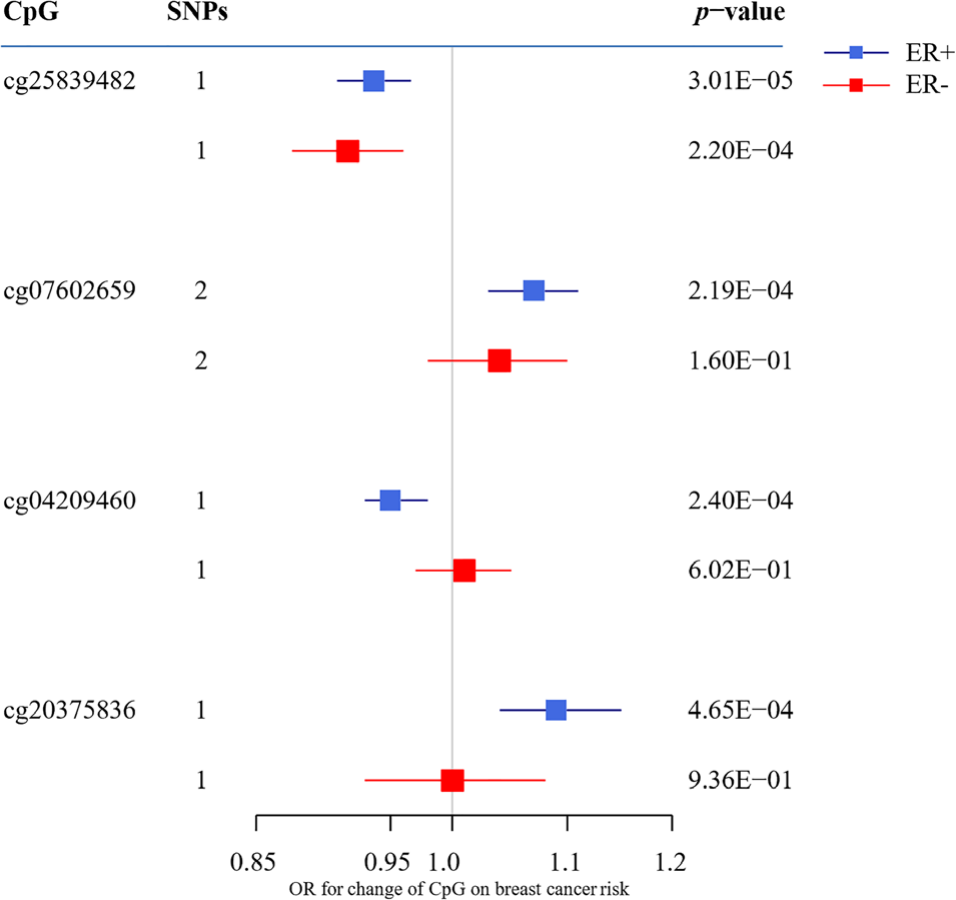
The association between CpG sites (passing the multiple testing correction) and the breast cancer risk. OR indicates odds ratio; ER, estrogen receptor; MR, mendelian randomization.

To assess the causal effect of DNA methylation at smoking-related CpG sites on breast cancer, we extracted the identified SNPs for mQTLs in the GWAS summary data from BCAC Consortium and conducted a two-sample MR. The top 10 results are shown in **Table 2** (full results in **Supplementary Tables S2**, **S3**). For ER+ breast cancer, we observed four CpG-cancer effect estimates that survived in multiple comparisons test (*q*-value after false discovery rate < 0.05): using rs8035987 as an instrument, we found that the smoking-related DNA methylation level of cg2583948 was associated with the risk of ER+ breast cancer [OR = 0.94, 95% CI (0.91–0.97)]. Similarly, the CpG sites of cg0760265 [OR = 1.07, 95% CI (1.03–1.11)], cg0420946 [OR = 0.95, 95% CI (0.93–0.98)], and cg2037583 [OR =1.09, 95% CI (1.04–1.15)] showed causal effects on ER+ breast cancer risk. For ER− breast cancer, we found no significant smoking-related CpG sites after false discovery rate correction. Moreover, in these four smoking-related CpG sites identified in ER+ breast cancer, only cg25839482 showed a nominally significant effect on ER− breast cancer risk [OR =0.92, 95% CI (0.88–0.96)], but failed to pass the multiple test correction (*q*-value = 0.085) (**Figure 2**).

**Table 2:** Two-sample Mendelian randomization estimations showing the effect of smoking-related methylation on the risk of breast cancer subtypes (top ten CpGs according to *p*-value).

CpG	Chr	Position	Method	SNPs	Odds ratio	95% CI		*p*-value	*q*-value	Gene	Power
**ER+**											
cg2583948	15	75931953	Wald ratio	1	0.94	0.91	0.97	3.01E-05	**1.03E-02**	*IMP3*	1.00
cg0760265*	17	80844196	IVW	2	1.07	1.03	1.11	2.19E-04	**2.68E-02**	*TBCD*	0.91
cg0420946	17	4711018	Wald ratio	1	0.95	0.93	0.98	2.40E-04	**2.74E-02**	*PLD2*	1.00
cg2037583	13	99135543	Wald ratio	1	1.09	1.04	1.15	4.65E-04	**3.99E-02**	*STK24*	0.97
cg1227506	1	45279329	Wald ratio	1	0.93	0.89	0.97	8.06E-04	5.34E-02	*BTBD19*	0.93
cg2607605*	5	421317	IVW	2	1.05	1.02	1.08	1.04E-03	5.95E-02	*AHRR*	0.79
cg1782334	10	80848143	Wald ratio	1	1.04	1.02	1.07	1.62E-03	7.78E-02	*ZMIZ1*	1.00
cg0156570	14	103245090	Wald ratio	1	1.09	1.03	1.14	2.19E-03	9.07E-02	*TRAF3*	0.97
cg1870825	22	39545030	Wald ratio	1	0.96	0.94	0.99	2.82E-03	1.01E-01	*CBX7*	0.97
cg0318838	2	233245886	Wald ratio	1	0.93	0.88	0.97	3.39E-03	1.09E-01	*ALPP*	0.87
**ER-**											
cg2583948	15	75931953	Wald ratio	1	0.92	0.88	0.96	2.20E-04	8.47E-02	*IMP3*	0.98
cg1882444	15	89154058	Wald ratio	1	0.94	0.91	0.98	2.10E-03	3.64E-01	*MIR7-2*	0.97
cg1261648	11	62379063	Wald ratio	1	0.87	0.79	0.96	3.31E-03	4.25E-01	*ROM1*	0.97
cg2761893	8	71581788	Wald ratio	1	1.13	1.04	1.23	5.33E-03	4.91E-01	*XKR9*	0.99
cg2670350	2	113404661	Wald ratio	1	0.93	0.88	0.99	1.30E-02	5.77E-01	*SLC20A1*	0.91
cg1693695	17	57915665	Wald ratio	1	0.91	0.85	0.98	1.44E-02	5.84E-01	*TMEM49*	0.90
cg1782334	10	80848143	Wald ratio	1	1.05	1.01	1.09	1.60E-02	5.91E-01	*ZMIZ1*	0.96
cg1345216	13	96204518	Wald ratio	1	1.09	1.02	1.18	1.76E-02	5.96E-01	*CLDN10*	0.82
cg2403312	16	30485383	Wald ratio	1	1.04	1.01	1.07	1.81E-02	5.98E-01	*ITGAL*	0.84
cg1858406	2	64975916	Wald ratio	1	0.91	0.84	0.98	1.98E-02	6.02E-01		0.78

Bold font indicates the estimation passed the FDR test.

CI indicates confidence interval; IVW, Inverse variance weighted; ER, estrogen receptor; SNP, single nucleotide polymorphism. Statistical power was calculated by a web tool (https://shiny.cnsgenomics.com/mRnd/) (23).

* Cochran’s Q-test for cg0760265 (Q-statistics, 0.19; p = 0.66) and cg2607605 (Q-statistics, 0.05; p = 0.83).

### Sensitivity Analyses

By calculating the variance explained in the smoking-related CpG sites and breast cancer subtypes by the instrumenting SNPs, and testing the difference of variance between exposure and outcome, we found that all four smoking-related CpG sites had a larger variance than that in ER+ breast cancer (all *p* < 1.83× 10^−11^), suggesting a forward causal direction from smoking-related DNA methylation to breast cancer risk (**Table 3**). Further colocalization analysis provided the posterior probability of five hypotheses (PP.H0–H4), supporting the idea that the CpG site of cg2583948 and ER+ breast cancer shared a common SNP (PP.H0–H3 < 0.05, and PP.H4 = 0.958) (**Table 3**). Although the PP.H4 of cg04209460 was close to the threshold of 0.8, PP.H1 was not satisfied (PP.H1 = 0.212, H1 represents only the CpG site that has a genetic association in the given region). There was no evidence that the CpG sites of cg07602659 and cg20375836 shared a common SNP with ER+ breast cancer. Moreover, a phenome-wide association study suggested that cg20375836 is associated with body mass index (*p* = 5.86E-07) (**Supplementary Table S4**), which has been proved to be a significant risk factor of breast cancer.

**Table 3:** MR Steiger directionality test and colocalization for four top findings in ER+ breast cancer.

CpG	PP.H0	PP.H1	PP.H2	PP.H3	PP.H4	Steiger *p*-value	Evidence
**ER+ breast cancer**							
cg2583948	4.38E-27	4.19E-02	1.00E-28	0	9.58E-01	1.01E-34	Strong
cg07602659	3.43E-07	8.83E-01	8.13E-11	9.29E-05	1.17E-01	1.86E-21	Weak
cg04209460	5.52E-43	2.12E-01	2.05E-45	0	7.88E-01	1.92E-57	Moderate
cg20375836	5.82E-06	3.67E-01	1.01E-08	0	6.33E-01	1.83E-11	Weak

ER indicates estrogen receptor; PP, posterior probability.

## Discussion

In this study, we performed MR analyses to test if genetic evidence supported a causal relationship of smoking-related DNA methylation with breast cancer risk. Our MR results showed that DNA methylation may be a vital bridge linking smoking and breast cancer, especially for the subtype of ER+ breast cancer.

The associations between smoking and DNA methylation have been established based on epigenome-wide association analyses (24–26). A recent MR study assessing the role of genome-wide DNA methylation between smoking and risk of lung cancer identified 75 significant CpG sites from the Trøndelag Health Study (HUNT) (27). The top DNA methylation sites around or within genes, such as *AHRR*, *F2RL3*, *RARA*, *MGAT3*, *GPR15*, and *PRSS23*, were proved to be associated with smoking. Of these, DNA methylation at cg05575921 in the *AHRR* gene has been found to be most strongly influenced by smoking in previous studies, but most restricted to lung cancer or chronic obstructive pulmonary disease. Only a few studies have investigated the association between DNA methylation and breast cancer risk, indicating that both DNA hypo-methylation and DNA hyper-methylation may be significantly associated with breast cancer (28).

However, we failed to extract instrumental SNPs for the DNA methylation at cg05575921 in our study. When we used two SNPs (rs72711366 and rs77454118) to proxy the CpG site of cg2607605 (with *AHRR* expression), the effect of methylation on the risk of ER+ breast cancer achieved a nominal significance but did not pass the multiple testing threshold (OR = 1.05, 95% CI [1.02–1.08], *p* = 1.04E-03, *q* = 5.95E-02). We finally identified the DNA methylation at cg2583948, which locates at the promoter region of *IMP3* and inhibits *IMP3* expression (29), as a crucial mediator between smoking and ER+ breast cancer. The *IMP3* gene encodes the human and mouse homologs of the yeast U3 snoRNP-associated protein Imp3 (30). The protein Imp3 localizes to nucleoli and interacts with the U3 snoRNA, and is essential for the early cleavage steps in pre-rRNA processing (31). As reported in a previous study, pre-rRNA processing plays a key role in ribosome synthesis, which leads to cell growth and represents specific hallmarks of cancer cells (32). Furthermore, increasing evidence indicates the existence of a strong relationship between the aberrant rRNA synthesis and the development of cancers: on the one hand, altered rRNA processing may reduce the stability of the *p53* gene, causing *p53* to lose its tumor suppressor function; on the other hand, abnormal ribosomal biosynthesis causes altered translation and may result in increased translation of oncogenes and impaired translation of tumor suppressor genes (33). Overexpression of *IMP3* was found in colorectal cancer tissue, and downregulation of *IMP3* suppressed the protein translation rates and cell growth in colorectal cancer cells (34). However, few studies focused on the relationship of *IMP3* expression with other cancer types including breast cancer. Therefore, further large-scale epigenetic and basic studies are required to confirm this new finding.

In addition, we observed a distinct effect between DNA methylation and different subtypes of breast cancer. Compared with ER− breast cancer, DNA methylation seemed to play a more important role in linking smoking and ER+ breast cancer. This was consistent with findings from prior studies, reporting that different subtypes of breast cancer display different patterns of DNA methylation. Specifically, ER+/luminal breast cancer is characterized by a remarkably higher frequency of DNA methylation compared to ER−/basal-like tumors, and a large amount of genes are differentially methylated in different breast cancer subtypes (35, 36). Taken together, these findings suggest that DNA methylation profiles could be more active in ER+ breast cancer, resulting in more mediation effects linking smoking behavior and the risk of ER+ breast cancer.

In this study, modification of cg25839482 for the *IMP3* gene was first reported to be associated with breast cancer using two-sample MR combined with colocalization analysis. There are still several shortages in our study. One is the relatively small sample sizes for smoking-related DNA methylation, which provides a limited statistical power. Second is the deficiency of tissue-specific data because the DNA methylation level in blood cannot accurately reflect the methylation level in the breast tissue. Third, the samples of the methylation cohort are middle-aged women, while the samples from the breast cancer cohort are a whole age group. The difference in age between the two cohorts may lead to bias.

## Conclusions

In summary, our MR results provided evidence that DNA methylation modification in blood, especially the CpG site of cg2583948, seems to represent a causal pathway linking smoking and the ER+ breast cancer risk, other than ER− breast cancer, which offers a novel insight into probing potential mechanisms and intervention targets for different breast cancer subtypes.

## Data Availability Statement

The original contributions presented in the study are included in the article/**Supplementary Material**. Further inquiries can be directed to the corresponding authors.

## Author Contributions

HT: Data collection, formal analysis, statistical analysis, and writing—original draft. DY: Data collection and formal analysis. CH and PM: Methodology, writing—review and editing, and supervision. All authors contributed to the article and approved the submitted version.

## Funding

This work was supported by Hunan Cancer Hospital Climb Plan (ZX2020005).

## Conflict of Interest

The authors declare that the research was conducted in the absence of any commercial or financial relationships that could be construed as a potential conflict of interest.

## Publisher’s Note

All claims expressed in this article are solely those of the authors and do not necessarily represent those of their affiliated organizations, or those of the publisher, the editors and the reviewers. Any product that may be evaluated in this article, or claim that may be made by its manufacturer, is not guaranteed or endorsed by the publisher.

## Supplementary Material

Supplementary Figure S1 Instrumental variable (IV) assumptions of Mendelian randomization.
