# Supplementary figures and images for: Smoking, DNA Methylation, and Breast Cancer: A Mendelian Randomization Study
Source: Front Oncol. 2021 Sep 28;11:745918. doi: 10.3389/fonc.2021.745918 (PMC8507148; doi:10.3389/fonc.2021.745918)

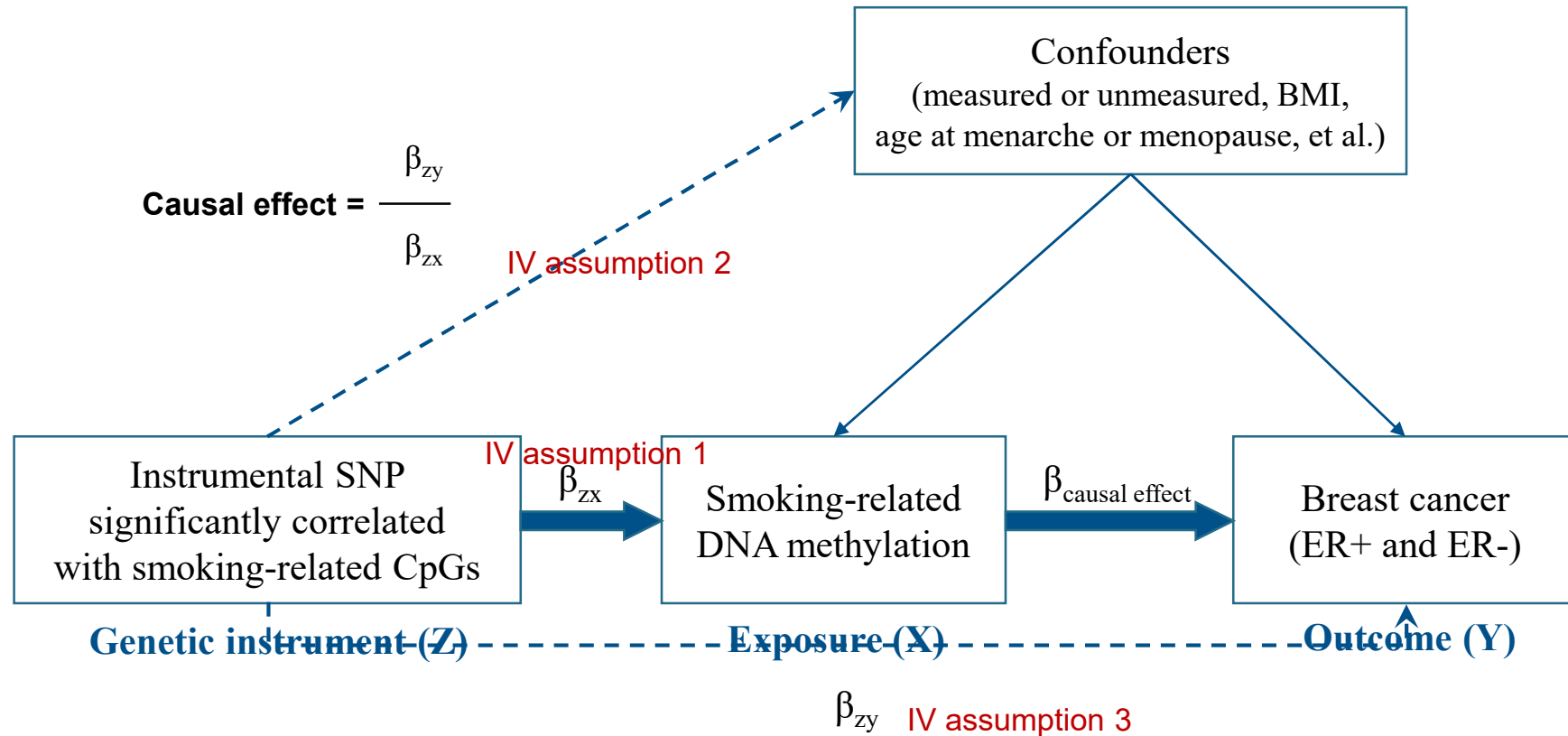

Supplement: Supplementary Figure S1 — Instrumental variable (IV) assumptions of Mendelian randomization. [file Presentation_1.pdf]
