# Supplementary material for: Smoking, DNA Methylation, and Breast Cancer: A Mendelian Randomization Study
Source: Front Oncol. 2021 Sep 28;11:745918. doi: 10.3389/fonc.2021.745918 (PMC8507148; doi:10.3389/fonc.2021.745918)
Supplement: Supplementary file 2 [file Table_1.docx]

Table S1. Single-nucleotide polymorphisms associated with smoking-related CpG sites.

| exposure | chr | Gene | SNP | snp_pos | beta | se | pval | effect_allele | other_allele | eaf | r2 | fstat |
| --- | --- | --- | --- | --- | --- | --- | --- | --- | --- | --- | --- | --- |
| cg00022866 | 11 | CCDC88B | rs7122759 | 64089673 | -0.9578 | 0.030771 | 5.27E-213 | T | C | 0.3262 | 0.621783 | 1640.696 |
| cg00024404 | 5 | SERINC5 | rs1126176 | 79552184 | 0.3353 | 0.046492 | 2.76E-13 | A | G | 0.3688 | 0.052101 | 54.85446 |
| cg00073460 | 6 | ZC3H12D | rs12660849 | 149858994 | 0.468 | 0.065059 | 3.16E-13 | A | T | 0.08688 | 0.051846 | 54.57185 |
| cg00099441 | 2 |  | rs7560331 | 11264830 | 0.3761 | 0.039551 | 9.61E-22 | G | A | 0.3617 | 0.087914 | 96.19454 |
| cg00153942 | 11 | KRTAP5-8 | rs7940512 | 71251875 | 0.3629 | 0.028863 | 1.48E-36 | C | T | 0.4332 | 0.147731 | 172.9922 |
| cg00177243 | 10 | CACNB2 | rs76077446 | 18497253 | 1.282 | 0.094898 | 6.90E-42 | A | T | 0.05024 | 0.168332 | 201.9977 |
| cg00178850 | 1 | EDARADD | rs6700178 | 236544350 | -0.3818 | 0.058477 | 3.31E-11 | G | C | 0.1755 | 0.043136 | 44.98999 |
| cg00205605 | 1 |  | rs867436 | 2523723 | 0.6416 | 0.036984 | 1.02E-67 | T | C | 0.3251 | 0.261442 | 353.281 |
| cg00383167 | 18 |  | rs62103233 | 77635572 | 0.363 | 0.04498 | 3.51E-16 | G | A | 0.2849 | 0.064486 | 68.79314 |
| cg00498211 | 2 | SLC1A4 | rs7604992 | 65215487 | 0.736301 | 0.053292 | 1.01E-43 | G | C | 0.1578 | 0.1753 | 105.9622 |
| cg00498211 | 2 | SLC1A4 | rs34452143 | 65188125 | -0.37392 | 0.057295 | 3.37E-11 | A | G | 0.1324 | 0.0431 | 22.45296 |
| cg00574379 | 7 |  | rs111669732 | 38354558 | 0.7014 | 0.042191 | 2.32E-62 | C | A | 0.2565 | 0.243014 | 320.3866 |
| cg00579402 | 19 | FUT6 | rs778798 | 5839613 | 0.6568 | 0.039571 | 3.60E-62 | A | C | 0.2707 | 0.24235 | 319.2308 |
| cg00580497 | 5 |  | rs7726442 | 1157313 | 0.5161 | 0.04608 | 2.04E-29 | C | T | 0.3357 | 0.119376 | 135.2869 |
| cg00668559 | 6 | NFKBIE | rs730775 | 44232074 | 0.7161 | 0.03462 | 2.37E-95 | G | A | 0.4161 | 0.349643 | 536.5415 |
| cg00689225 | 14 | NFKBIA | rs72666636 | 35837014 | -0.4051 | 0.035481 | 1.71E-30 | T | G | 0.2252 | 0.123705 | 140.8864 |
| cg00689360 | 4 | ADH1C | rs4269183 | 100190185 | -0.35164 | 0.043228 | 2.07E-16 | G | A | 0.4492 | 0.065464 | 34.9198 |
| cg00689360 | 4 | ADH1C | rs2213041 | 100247351 | 0.386459 | 0.056767 | 4.95E-12 | A | C | 0.1773 | 0.046698 | 24.41925 |
| cg00756943 | 15 |  | rs79722842 | 91203393 | 1.12894 | 0.086724 | 4.86E-39 | C | G | 0.05142 | 0.157389 | 93.11349 |
| cg00756943 | 15 |  | rs4932356 | 91160507 | -0.49589 | 0.04437 | 2.67E-29 | A | T | 0.2405 | 0.118905 | 67.27358 |
| cg00806481 | 1 | PRDM16 | rs1569419 | 2996602 | 0.9239 | 0.04133 | 5.50E-111 | T | C | 0.2411 | 0.394819 | 651.0945 |
| cg00835193 | 19 | LINGO3 | rs56291613 | 2323197 | 0.3839 | 0.045028 | 7.59E-18 | A | G | 0.3741 | 0.071551 | 76.91126 |
| cg00893603 | 13 | ATP8A2 | rs9553696 | 26575440 | -0.5651 | 0.039505 | 1.02E-46 | G | A | 0.4025 | 0.186572 | 228.9066 |
| cg00917251 | 1 | RCAN3 | rs189552363 | 24829685 | 0.7476 | 0.079486 | 2.59E-21 | T | G | 0.06442 | 0.086118 | 94.04456 |
| cg00981651 | 20 | PCIF1 | rs11697323 | 44551579 | 0.4887 | 0.040677 | 1.50E-33 | T | C | 0.4551 | 0.135901 | 156.9604 |
| cg00995520 | 1 | KCNA3 | rs2640487 | 111206885 | 0.2505 | 0.040273 | 2.49E-10 | T | C | 0.477 | 0.039347 | 40.87656 |
| cg01020987 | 1 | C1orf174 | rs12092962 | 3814883 | 0.5183 | 0.06083 | 7.94E-18 | G | C | 0.1525 | 0.071467 | 76.81362 |
| cg01062937 | 16 | ZFPM1 | rs870021 | 88536774 | -0.1724 | 0.026883 | 7.14E-11 | A | T | 0.2985 | 0.041692 | 43.4191 |
| cg01185345 | 13 | STK24 | rs7336828 | 99193474 | 0.4261 | 0.049843 | 6.22E-18 | A | G | 0.1844 | 0.071916 | 77.33386 |
| cg01268763 | 14 | DDHD1 | rs2552401 | 53655979 | -0.4288 | 0.058558 | 1.22E-13 | G | C | 0.1584 | 0.053629 | 56.55438 |
| cg01289343 | 13 | RASA3 | rs1556126 | 114805743 | 0.4963 | 0.041124 | 7.75E-34 | C | T | 0.4314 | 0.137036 | 158.4792 |
| cg01294327 | 19 | LINGO3 | rs57678395 | 2350114 | 0.8049 | 0.037225 | 5.49E-104 | C | T | 0.4054 | 0.374996 | 598.7893 |
| cg01314044 | 11 |  | rs2509324 | 94245740 | 0.39 | 0.042035 | 8.63E-21 | A | G | 0.4078 | 0.083935 | 91.44285 |
| cg01328473 | 2 | PXDN | rs11127326 | 1712139 | -0.3037 | 0.041919 | 2.16E-13 | A | G | 0.2287 | 0.052551 | 55.35534 |
| cg01360605 | 20 |  | rs6015692 | 58784339 | 0.2702 | 0.047426 | 6.09E-09 | C | G | 0.2695 | 0.033323 | 34.4024 |
| cg01435643 | 13 | MCF2L | rs3011511 | 113684919 | -0.3894 | 0.045469 | 5.45E-18 | A | G | 0.2423 | 0.072159 | 77.61513 |
| cg01439670 | 11 | LRRC32 | rs4945097 | 76368553 | 0.3134 | 0.045653 | 3.33E-12 | G | A | 0.5 | 0.047443 | 49.70626 |

| cg01447828 | 19 | PRX | rs118110133 | 40967838 | -1.292 | 0.102722 | 1.40E-36 | T | C | 0.04137 | 0.147822 | 173.117 |
| --- | --- | --- | --- | --- | --- | --- | --- | --- | --- | --- | --- | --- |
| cg01465596 | 1 | HIVEP3 | rs2786487 | 42368339 | -0.4882 | 0.033831 | 1.66E-47 | G | C | 0.4309 | 0.189514 | 233.36 |
| cg01471372 | 8 | TACC1 | rs6993024 | 38577517 | -0.24147 | 0.043228 | 1.16E-08 | G | T | 0.3582 | 0.032104 | 16.53448 |
| cg01471372 | 8 | TACC1 | rs34153723 | 38590994 | 0.412651 | 0.05234 | 1.58E-15 | C | A | 0.2098 | 0.061696 | 32.77755 |
| cg01498900 | 14 | ACOT2 | rs2215013 | 74084833 | -0.4251 | 0.03878 | 2.92E-28 | G | A | 0.4758 | 0.114705 | 129.3072 |
| cg01561259 | 17 | RPTOR | rs1006809 | 78617956 | 0.2388 | 0.036774 | 4.19E-11 | C | T | 0.3848 | 0.042694 | 44.50855 |
| cg01565703 | 14 | TRAF3 | rs11850030 | 103256199 | -0.3541 | 0.052586 | 8.27E-12 | G | C | 0.1868 | 0.045738 | 47.83419 |
| cg01643605 | 16 |  | rs8053800 | 29043450 | 0.2847 | 0.037171 | 9.36E-15 | T | G | 0.331 | 0.058401 | 61.89878 |
| cg01651915 | 8 |  | rs1498170 | 55746328 | -0.3224 | 0.037395 | 3.31E-18 | T | A | 0.3522 | 0.073076 | 78.67965 |
| cg01937809 | 1 | ZC3H12A | rs115755325 | 37932644 | -0.8061 | 0.125696 | 7.13E-11 | A | G | 0.01182 | 0.041694 | 43.42106 |
| cg01940297 | 8 | MIR1207 | rs2648876 | 129072966 | 0.4898 | 0.043103 | 3.18E-30 | A | G | 0.3605 | 0.122626 | 139.4857 |
| cg02032558 | 4 |  | rs17031978 | 155663171 | 0.633944 | 0.087542 | 2.22E-13 | T | C | 0.0792 | 0.052507 | 27.62519 |
| cg02068690 | 2 | DTNB | rs2304426 | 25602142 | 0.192 | 0.035 | 2.06E-08 | G | A | 0.3652 | 0.031025 | 31.95479 |
| cg02186444 | 17 | ARMC7 | rs11077776 | 73137187 | 0.3392 | 0.038411 | 5.20E-19 | A | G | 0.3446 | 0.076466 | 82.63194 |
| cg02279625 | 15 | SH2D7 | rs12910083 | 78384669 | 0.9678 | 0.108983 | 3.34E-19 | T | C | 0.03901 | 0.077277 | 83.5813 |
| cg02373104 | 7 | MIR548F3 | rs2708257 | 147592449 | 0.5127 | 0.038797 | 3.59E-40 | C | A | 0.3546 | 0.16175 | 192.5762 |
| cg02405476 | 20 | UBE2C | rs4625980 | 44414086 | 0.3705 | 0.043555 | 8.96E-18 | C | T | 0.331 | 0.071245 | 76.55728 |
| cg02462416 | 11 |  | rs7107675 | 2034097 | -0.686 | 0.053615 | 8.74E-38 | A | T | 0.2417 | 0.152521 | 179.6106 |
| cg02508743 | 8 | LYN | rs72653917 | 56892844 | -0.4111 | 0.050317 | 1.54E-16 | A | T | 0.1956 | 0.066009 | 70.53243 |
| cg02532700 | 22 | NCF4 | rs4821544 | 37258503 | 0.5065 | 0.040694 | 7.31E-36 | C | T | 0.3191 | 0.145013 | 169.2697 |
| cg02629070 | 6 |  | rs13219238 | 41398931 | -0.3786 | 0.061332 | 3.35E-10 | T | C | 0.1696 | 0.038784 | 40.26809 |
| cg02660097 | 11 |  | rs921675 | 68869034 | 0.518983 | 0.058766 | 5.17E-19 | A | G | 0.1525 | 0.076474 | 41.27902 |
| cg02660097 | 11 |  | rs10750842 | 68860898 | 0.55511 | 0.042007 | 3.61E-40 | C | A | 0.4793 | 0.161741 | 96.18507 |
| cg02672759 | 19 |  | rs35790907 | 39730755 | -0.2765 | 0.050254 | 1.88E-08 | T | A | 0.2677 | 0.031199 | 32.13964 |
| cg02704502 | 11 |  | rs7107675 | 2034097 | -0.628 | 0.050925 | 3.05E-35 | A | T | 0.2417 | 0.142575 | 165.9508 |
| cg02782510 | 3 | B3GALNT1 | rs28693895 | 160610687 | -0.8112 | 0.126026 | 6.10E-11 | C | T | 0.02187 | 0.041987 | 43.73929 |
| cg02787737 | 11 |  | rs80235995 | 133928704 | -0.5279 | 0.064323 | 1.13E-16 | T | C | 0.1082 | 0.066572 | 71.17705 |
| cg02871659 | 16 | SNHG9 | rs397435 | 2010138 | -0.7164 | 0.046863 | 4.66E-53 | G | A | 0.1809 | 0.209931 | 265.1813 |
| cg02959759 | 12 | CACNA1C | rs11062335 | 2841592 | 0.4428 | 0.067722 | 3.11E-11 | T | C | 0.1336 | 0.043254 | 45.11913 |
| cg02998240 | 1 |  | rs12562207 | 16508059 | 0.7839 | 0.034281 | 4.98E-116 | G | A | 0.3723 | 0.408715 | 689.8503 |
| cg03051880 | 6 | MAN1A1 | rs195075 | 119653287 | -0.3791 | 0.048211 | 1.87E-15 | C | T | 0.2689 | 0.06139 | 65.27388 |
| cg03144619 | 1 | GALNT2 | rs12139970 | 230406460 | -0.43713 | 0.047279 | 1.17E-20 | T | G | 0.2849 | 0.083386 | 45.3495 |
| cg03144619 | 1 | GALNT2 | rs6698963 | 230415025 | -0.47405 | 0.044751 | 1.60E-26 | G | A | 0.4639 | 0.107623 | 60.12029 |
| cg03188382 | 2 | ALPP | rs12620827 | 233244703 | 0.5434 | 0.083512 | 3.84E-11 | C | T | 0.07565 | 0.042858 | 44.68746 |
| cg03222009 | 2 | HS6ST1 | rs62158068 | 129062947 | -0.4084 | 0.049639 | 9.57E-17 | T | C | 0.2855 | 0.066885 | 71.5361 |
| cg03234777 | 11 | AMICA1 | rs7926717 | 118121399 | -0.5363 | 0.049418 | 9.73E-28 | T | G | 0.1856 | 0.11258 | 126.6083 |
| cg03333699 | 7 | ADAP1 | rs4722409 | 947236 | 0.3482 | 0.042079 | 6.43E-17 | C | T | 0.4066 | 0.067618 | 72.37707 |
| cg03373393 | 17 | HAP1 | rs7406082 | 39896062 | -0.36733 | 0.053544 | 3.44E-12 | T | C | 0.1826 | 0.047383 | 24.79541 |

| cg03480935 | 15 | SMAD3 | rs17214419 | 67416657 | 0.3739 | 0.036513 | 6.56E-25 | T | A | 0.3333 | 0.101018 | 112.1448 |
| --- | --- | --- | --- | --- | --- | --- | --- | --- | --- | --- | --- | --- |
| cg03519967 | 1 | MAN1C1 | rs2744779 | 26014410 | -0.6464 | 0.076395 | 1.32E-17 | T | C | 0.05969 | 0.070531 | 75.73136 |
| cg03554335 | 17 | C17orf72 | rs62070902 | 62070358 | 0.3075 | 0.036433 | 1.58E-17 | T | G | 0.3528 | 0.070197 | 75.3458 |
| cg03609435 | 17 |  | rs2028069 | 34966917 | -0.2712 | 0.033924 | 6.51E-16 | C | T | 0.3753 | 0.063343 | 67.49135 |
| cg03710029 | 17 | SLC38A10 | rs8076302 | 79265701 | 0.5282 | 0.032873 | 2.14E-58 | T | C | 0.2234 | 0.229083 | 296.5623 |
| cg03785755 | 6 |  | rs112751982 | 26191958 | 0.4167 | 0.055285 | 2.40E-14 | T | C | 0.1318 | 0.05665 | 59.93164 |
| cg03789791 | 16 |  | rs252287 | 29165923 | 0.4881 | 0.060406 | 3.23E-16 | C | T | 0.172 | 0.064639 | 68.9677 |
| cg03864215 | 11 | KCNJ11 | rs5213 | 17408404 | 0.3927 | 0.045865 | 5.55E-18 | C | T | 0.3664 | 0.072127 | 77.57827 |
| cg03884592 | 1 | HIVEP3 | rs2786487 | 42368339 | -0.6535 | 0.038618 | 1.54E-64 | G | C | 0.4309 | 0.250555 | 333.6514 |
| cg03970900 | 1 |  | rs983816 | 222446350 | 0.4743 | 0.038729 | 8.74E-35 | G | A | 0.3629 | 0.140778 | 163.516 |
| cg04017131 | 8 | ANGPT2 | rs10108504 | 6422030 | 0.66213 | 0.087054 | 1.41E-14 | C | T | 0.07329 | 0.057634 | 30.48753 |
| cg04017131 | 8 | ANGPT2 | rs2442626 | 6374306 | 0.612292 | 0.100255 | 5.06E-10 | C | T | 0.0461 | 0.038007 | 19.69506 |
| cg04019522 | 2 | BIN1 | rs11692858 | 127867601 | -0.57964 | 0.077231 | 3.06E-14 | T | C | 0.04965 | 0.056195 | 29.68102 |
| cg04019522 | 2 | BIN1 | rs6709706 | 127844357 | 0.218 | 0.035135 | 2.74E-10 | T | C | 0.4285 | 0.039162 | 20.31807 |
| cg04019636 | 1 |  | rs2819360 | 201977254 | -0.3782 | 0.043277 | 1.17E-18 | T | C | 0.445 | 0.074973 | 80.88751 |
| cg04144533 | 1 | RASSF5 | rs72752997 | 206682038 | -0.4367 | 0.044868 | 1.09E-22 | G | A | 0.3676 | 0.091843 | 100.9291 |
| cg04209460 | 17 | PLD2 | rs10852865 | 4707619 | -0.8186 | 0.051708 | 9.46E-57 | A | C | 0.1643 | 0.22323 | 286.8079 |
| cg04232972 | 16 |  | rs72793353 | 30623514 | -0.8271 | 0.100804 | 1.15E-16 | T | C | 0.0461 | 0.066542 | 71.14313 |
| cg04263702 | 7 | FBXL18 | rs4559154 | 5530607 | -0.51209 | 0.049006 | 7.36E-26 | G | T | 0.3126 | 0.104916 | 58.43081 |
| cg04263702 | 7 | FBXL18 | rs10807949 | 5528375 | -0.31221 | 0.046421 | 8.75E-12 | T | C | 0.4131 | 0.045632 | 23.83537 |
| cg04337534 | 11 | GAL3ST3 | rs577013 | 65832973 | 0.2179 | 0.038249 | 6.10E-09 | C | T | 0.477 | 0.033319 | 34.39814 |
| cg04396998 | 19 | PPP1R15A | rs564196 | 49376582 | 0.6782 | 0.058682 | 3.39E-31 | A | G | 0.133 | 0.126524 | 144.5613 |
| cg04411044 | 1 |  | rs75639682 | 36175044 | -0.54373 | 0.0767 | 6.75E-13 | C | T | 0.05851 | 0.050427 | 26.47274 |
| cg04411044 | 1 |  | rs67906703 | 36178590 | 0.714353 | 0.077541 | 1.59E-20 | T | C | 0.06206 | 0.082822 | 45.01486 |
| cg04506190 | 3 | PLXND1 | rs9837325 | 129315831 | -0.3709 | 0.056609 | 2.84E-11 | A | C | 0.1885 | 0.043423 | 45.30341 |
| cg04545963 | 14 | NFKBIA | rs72666636 | 35837014 | -0.4716 | 0.031888 | 8.59E-50 | T | G | 0.2252 | 0.197987 | 246.3681 |
| cg04666465 | 16 | GGA2 | rs4967956 | 23489025 | -0.8763 | 0.04635 | 5.08E-80 | G | A | 0.1909 | 0.302085 | 431.9742 |
| cg04806562 | 17 | TEX19 | rs79159073 | 80284818 | 0.5095 | 0.093014 | 2.15E-08 | G | A | 0.05083 | 0.030939 | 31.86332 |
| cg04816394 | 5 | RNU5E | rs4704707 | 80533359 | -0.4351 | 0.047804 | 4.44E-20 | G | A | 0.1915 | 0.080955 | 87.90998 |
| cg04907244 | 7 | SNORD93 | rs11983782 | 22893795 | -0.5349 | 0.037608 | 3.30E-46 | G | C | 0.4492 | 0.18467 | 226.0446 |
| cg05122453 | 9 | SH2D3C | rs913990 | 130538720 | 0.3155 | 0.041027 | 7.36E-15 | G | T | 0.3345 | 0.058847 | 62.40141 |
| cg05183538 | 16 | ZFPM1 | rs71394100 | 88534619 | -0.1964 | 0.035669 | 1.83E-08 | T | C | 0.3115 | 0.031244 | 32.18719 |
| cg05204104 | 2 | ARL4C | rs1464264 | 235199587 | -0.2324 | 0.035146 | 1.89E-11 | G | A | 0.422 | 0.044186 | 46.13608 |
| cg05214460 | 10 | ADD3 | rs12572438 | 111769236 | 0.5228 | 0.059252 | 5.56E-19 | C | T | 0.1288 | 0.076343 | 82.48785 |
| cg05379350 | 17 | GIT1 | rs62068577 | 27924878 | -0.2854 | 0.044387 | 6.39E-11 | G | T | 0.4078 | 0.041901 | 43.64598 |
| cg05542681 | 16 | FBXL16 | rs11865690 | 740682 | -0.6205 | 0.049995 | 1.13E-35 | T | C | 0.2187 | 0.144265 | 168.2491 |
| cg05593667 | 6 |  | rs7755718 | 35489971 | -1.141 | 0.076891 | 4.09E-50 | G | A | 0.05378 | 0.199175 | 248.2141 |
| cg05599106 | 19 | ZNF20 | rs113671908 | 12240965 | 0.5222 | 0.0643 | 2.31E-16 | T | C | 0.107 | 0.065262 | 69.67854 |

| cg05661533 | 5 | LPCAT1 | rs72717531 | 1490019 | 0.2447 | 0.04149 | 1.84E-09 | G | C | 0.2104 | 0.035575 | 36.81392 |
| --- | --- | --- | --- | --- | --- | --- | --- | --- | --- | --- | --- | --- |
| cg05720226 | 7 | ST7 | rs10278192 | 116784638 | 0.3679 | 0.036958 | 1.20E-23 | G | A | 0.1767 | 0.095808 | 105.7476 |
| cg05830220 | 16 | KLHDC4 | rs35381489 | 87769041 | 0.345594 | 0.058985 | 2.33E-09 | C | T | 0.1743 | 0.035134 | 18.15198 |
| cg05830220 | 16 | KLHDC4 | rs28367166 | 87755155 | -0.36445 | 0.043563 | 2.98E-17 | A | G | 0.4498 | 0.069038 | 36.96767 |
| cg05877788 | 17 | TP53I13 | rs565977 | 27898828 | 0.6556 | 0.040984 | 6.76E-58 | T | C | 0.3014 | 0.227312 | 293.5952 |
| cg05886626 | 15 | THBS1 | rs2618157 | 39844315 | -0.5256 | 0.071827 | 1.26E-13 | A | G | 0.1087 | 0.053557 | 56.47488 |
| cg05914034 | 7 | FBXL18 | rs4559154 | 5530607 | -0.38682 | 0.051566 | 3.16E-14 | G | T | 0.3126 | 0.056139 | 29.65006 |
| cg05914034 | 7 | FBXL18 | rs10807949 | 5528375 | -0.26595 | 0.048932 | 2.74E-08 | T | C | 0.4131 | 0.030488 | 15.67599 |
| cg05987787 | 6 | SYNJ2 | rs9456988 | 158448559 | 0.3094 | 0.045254 | 4.04E-12 | G | C | 0.4167 | 0.047078 | 49.30467 |
| cg06065019 | 2 |  | rs11695345 | 177361516 | 0.835 | 0.047777 | 1.07E-68 | T | G | 0.09693 | 0.264758 | 359.3758 |
| cg06193043 | 1 | NPPA | rs169158 | 11898228 | -1.114 | 0.052402 | 1.37E-100 | C | T | 0.1229 | 0.365139 | 573.9979 |
| cg06197751 | 3 | SGEF | rs399118 | 153927792 | -0.4544 | 0.04269 | 9.28E-27 | G | A | 0.3452 | 0.108592 | 121.5773 |
| cg06283478 | 4 | SORCS2 | rs35828773 | 7638700 | -0.2975 | 0.043527 | 4.11E-12 | A | G | 0.2796 | 0.047049 | 49.27355 |
| cg06321596 | 16 | XYLT1 | rs56399325 | 17562895 | -0.4315 | 0.046598 | 1.02E-20 | A | C | 0.3079 | 0.083628 | 91.07716 |
| cg06382664 | 11 | RELT | rs7131383 | 73071263 | -0.4054 | 0.046888 | 2.66E-18 | C | T | 0.2116 | 0.073474 | 79.14148 |
| cg06418475 | 19 | CPAMD8 | rs2608732 | 17008578 | 0.6109 | 0.039756 | 1.38E-53 | G | C | 0.4835 | 0.211854 | 268.2626 |
| cg06433467 | 5 |  | rs72759856 | 67730101 | 1.004 | 0.081813 | 6.41E-35 | A | C | 0.05201 | 0.14131 | 164.2353 |
| cg06435765 | 18 | CLUL1 | rs7244330 | 592581 | 0.3803 | 0.043913 | 2.35E-18 | T | C | 0.2784 | 0.073702 | 79.40701 |
| cg06459104 | 18 | EPB41L3 | rs11081195 | 5451640 | 0.63847 | 0.036315 | 1.72E-69 | C | T | 0.3918 | 0.267441 | 181.9912 |
| cg06459104 | 18 | EPB41L3 | rs112527405 | 5424367 | 1.38916 | 0.104515 | 1.30E-40 | C | A | 0.03191 | 0.163453 | 97.40212 |
| cg06478823 | 16 | ACSM3 | rs35518073 | 20740257 | 0.7815 | 0.067124 | 1.25E-31 | T | C | 0.1194 | 0.128258 | 146.8341 |
| cg06522681 | 19 | CCDC105 | rs1476593 | 15026125 | 0.5376 | 0.058398 | 1.69E-20 | A | G | 0.1944 | 0.082709 | 89.98572 |
| cg06670463 | 4 | UBE2D3 | rs13117052 | 103749747 | 0.526572 | 0.069918 | 2.51E-14 | C | G | 0.09752 | 0.056564 | 29.88764 |
| cg06791426 | 12 | FBRSL1 | rs2875247 | 133129836 | -0.6244 | 0.040317 | 2.11E-54 | C | T | 0.4586 | 0.214797 | 273.0089 |
| cg06861736 | 22 | MED15 | rs165792 | 20910883 | -0.2564 | 0.037659 | 4.93E-12 | G | A | 0.2796 | 0.046706 | 48.89622 |
| cg06880612 | 2 |  | rs2310185 | 102742168 | -0.2125 | 0.037789 | 9.37E-09 | C | T | 0.2866 | 0.03251 | 33.53565 |
| cg06951627 | 6 |  | rs112751982 | 26191958 | 0.4179 | 0.046467 | 1.20E-19 | T | C | 0.1318 | 0.079146 | 85.77633 |
| cg06968912 | 17 | PTRF | rs113366589 | 40516616 | -0.3273 | 0.055226 | 1.55E-09 | G | T | 0.09752 | 0.035905 | 37.16744 |
| cg07027613 | 12 | C1RL | rs3782925 | 7262024 | 0.3045 | 0.040293 | 2.06E-14 | T | C | 0.331 | 0.056935 | 60.25139 |
| cg07029024 | 11 |  | rs12807143 | 65249731 | 0.299036 | 0.049328 | 6.71E-10 | A | G | 0.1389 | 0.037476 | 19.40937 |
| cg07029024 | 11 |  | rs34582634 | 65220206 | -0.35612 | 0.041048 | 2.06E-18 | A | G | 0.195 | 0.073946 | 39.80552 |
| cg07090714 | 6 |  | rs12200128 | 6857183 | 0.501581 | 0.05938 | 1.49E-17 | G | T | 0.08688 | 0.070305 | 37.69735 |
| cg07090714 | 6 |  | rs17142630 | 6857640 | -0.48458 | 0.046492 | 9.75E-26 | G | A | 0.1413 | 0.104416 | 58.11975 |
| cg07180646 | 1 | TMEM51 | rs941456 | 15471731 | -0.63252 | 0.077419 | 1.54E-16 | T | C | 0.0526 | 0.066006 | 35.22928 |
| cg07202214 | 11 | LRRC32 | rs4945097 | 76368553 | 0.2713 | 0.045013 | 8.34E-10 | G | A | 0.5 | 0.037067 | 38.41726 |
| cg07207043 | 6 |  | rs7769136 | 7052225 | 1.043 | 0.052021 | 1.02E-89 | T | C | 0.1413 | 0.332558 | 497.262 |
| cg07277038 | 3 | LRRC33 | rs4916532 | 196373561 | 0.450773 | 0.062316 | 2.35E-13 | A | G | 0.1755 | 0.052398 | 18.35801 |
| cg07277038 | 3 | LRRC33 | rs34282171 | 196374894 | -0.69202 | 0.059884 | 3.45E-31 | G | A | 0.09397 | 0.126496 | 48.0786 |

| cg07277038 | 3 | LRRC33 | rs74626344 | 196375351 | 0.506402 | 0.086596 | 2.49E-09 | T | C | 0.08215 | 0.035008 | 12.04421 |
| --- | --- | --- | --- | --- | --- | --- | --- | --- | --- | --- | --- | --- |
| cg07278634 | 5 |  | rs7737970 | 1937863 | 0.316507 | 0.039067 | 2.71E-16 | C | G | 0.4214 | 0.064961 | 34.6331 |
| cg07278634 | 5 |  | rs901506 | 1959880 | -0.38066 | 0.045347 | 2.34E-17 | G | A | 0.2553 | 0.069481 | 37.2223 |
| cg07383757 | 11 | TRPC2 | rs72852291 | 3645520 | -0.3336 | 0.051708 | 5.53E-11 | C | T | 0.1247 | 0.042171 | 43.93951 |
| cg07421287 | 1 | KCNA3 | rs2640487 | 111206885 | 0.3282 | 0.039469 | 4.57E-17 | T | C | 0.477 | 0.068248 | 73.09992 |
| cg07450086 | 3 | SELT | rs114662645 | 150316713 | 0.6328 | 0.053719 | 2.48E-32 | A | G | 0.1684 | 0.131062 | 150.5284 |
| cg07502868 | 16 | LMF1 | rs72769410 | 997454 | -0.6537 | 0.076616 | 7.18E-18 | T | C | 0.07683 | 0.071652 | 77.02834 |
| cg07520810 | 10 | ARID5B | rs4948499 | 63825807 | 0.2451 | 0.036824 | 1.41E-11 | G | A | 0.4297 | 0.044742 | 46.74357 |
| cg07572233 | 1 |  | rs6660731 | 234688888 | -1.112 | 0.154286 | 2.85E-13 | A | G | 0.01478 | 0.052037 | 54.78367 |
| cg07602659 | 17 | TBCD | rs4986117 | 80809105 | 0.352891 | 0.047345 | 4.54E-14 | G | T | 0.2577 | 0.055463 | 29.27161 |
| cg07602659 | 17 | TBCD | rs8067926 | 80838588 | -0.24283 | 0.04198 | 3.64E-09 | A | G | 0.477 | 0.034292 | 17.70162 |
| cg07839313 | 19 | BST2 | rs12609479 | 17516628 | -0.3361 | 0.05695 | 1.80E-09 | G | A | 0.1667 | 0.03562 | 36.86178 |
| cg07941108 | 14 |  | rs58363992 | 90766361 | 0.2943 | 0.040075 | 1.04E-13 | C | T | 0.2884 | 0.053921 | 56.87997 |
| cg08035323 | 2 |  | rs11674642 | 9844069 | 0.2591 | 0.041499 | 2.14E-10 | C | G | 0.3298 | 0.039629 | 41.18163 |
| cg08097581 | 19 | AKAP8L | rs10419500 | 15486404 | -0.6244 | 0.046516 | 2.21E-41 | C | G | 0.2524 | 0.1664 | 199.2173 |
| cg08176694 | 12 | PITPNM2 | rs146903396 | 123411870 | 0.854351 | 0.111403 | 8.66E-15 | T | C | 0.03369 | 0.058543 | 30.99869 |
| cg08176694 | 12 | PITPNM2 | rs4148856 | 123450765 | 0.29378 | 0.047364 | 2.78E-10 | G | C | 0.2122 | 0.039138 | 20.30494 |
| cg08305533 | 22 | SFI1 | rs5998074 | 32054026 | -0.4208 | 0.06589 | 8.49E-11 | C | A | 0.09397 | 0.041366 | 43.0651 |
| cg08384239 | 2 | TANC1 | rs2288105 | 159992699 | 0.9259 | 0.079843 | 2.14E-31 | T | C | 0.08097 | 0.127321 | 145.6051 |
| cg08397758 | 10 | PYROXD2 | rs7091677 | 100167553 | 0.3825 | 0.047596 | 4.62E-16 | A | G | 0.2358 | 0.063975 | 68.21134 |
| cg08458637 | 1 | TSNAX-DI | rs2356384 | 231743681 | 0.3088 | 0.033501 | 1.52E-20 | G | A | 0.3345 | 0.082909 | 90.22395 |
| cg08472795 | 2 | HS6ST1 | rs13014434 | 129062842 | 0.417 | 0.050914 | 1.30E-16 | C | A | 0.1968 | 0.066315 | 70.88268 |
| cg08479476 | 2 | PCBP1 | rs4852428 | 70298396 | 0.9165 | 0.08845 | 1.85E-25 | G | A | 0.05792 | 0.103275 | 114.9384 |
| cg08712631 | 7 | MAD1L1 | rs56137415 | 1899322 | -0.8613 | 0.10497 | 1.15E-16 | T | C | 0.04314 | 0.066544 | 71.14558 |
| cg08787968 | 11 | WT1 | rs12293603 | 32446701 | 0.4067 | 0.053506 | 1.47E-14 | A | C | 0.2228 | 0.057562 | 60.95565 |
| cg08867399 | 2 | HS6ST1 | rs141232401 | 129060427 | -0.70398 | 0.130497 | 3.43E-08 | T | C | 0.02896 | 0.03006 | 10.28923 |
| cg08867399 | 2 | HS6ST1 | rs6761320 | 129050354 | -0.32806 | 0.046945 | 1.39E-12 | T | C | 0.422 | 0.049073 | 17.1329 |
| cg08867399 | 2 | HS6ST1 | rs67592012 | 129070028 | -0.40169 | 0.052597 | 1.11E-14 | T | C | 0.2861 | 0.05808 | 20.47151 |
| cg09006487 | 3 | RYBP | rs11717301 | 72397284 | 0.3477 | 0.047842 | 1.83E-13 | A | C | 0.2524 | 0.052866 | 55.70517 |
| cg09069072 | 1 | TMEM51 | rs6659540 | 15482697 | 0.8815 | 0.054973 | 3.64E-58 | T | C | 0.1643 | 0.228267 | 295.1933 |
| cg09099830 | 16 | ITGAL | rs7196129 | 30471109 | -0.3241 | 0.038987 | 4.66E-17 | T | C | 0.4728 | 0.068211 | 73.05828 |
| cg09206294 | 15 | MAPKBP1 | rs8025614 | 42142912 | 0.2479 | 0.042805 | 3.49E-09 | G | A | 0.4604 | 0.03437 | 35.52262 |
| cg09307264 | 17 | INCA1 | rs76101349 | 4902473 | -0.7456 | 0.079218 | 2.44E-21 | T | C | 0.07151 | 0.086231 | 94.17939 |
| cg09338136 | 5 | AHRR | rs150579152 | 397161 | 0.5877 | 0.076898 | 1.06E-14 | C | T | 0.09811 | 0.058161 | 61.6293 |
| cg09447622 | 6 | TCP11 | rs13196771 | 35109838 | -0.3852 | 0.062365 | 3.28E-10 | T | C | 0.1525 | 0.038827 | 40.31496 |
| cg09479241 | 17 | TLCD1 | rs9890687 | 26928363 | 0.56333 | 0.064897 | 1.97E-18 | G | A | 0.09397 | 0.074024 | 39.85082 |
| cg09479241 | 17 | TLCD1 | rs7218743 | 27219548 | 0.353016 | 0.042925 | 9.84E-17 | T | C | 0.2612 | 0.066833 | 35.70211 |
| cg09611599 | 11 |  | rs3808988 | 20384377 | -1.177 | 0.049066 | 1.86E-127 | C | T | 0.1217 | 0.439037 | 781.0823 |

| cg09635954 | 7 | PRR15 | rs73090506 | 29603199 | 0.422895 | 0.046434 | 4.22E-20 | G | A | 0.302 | 0.08105 | 43.96684 |
| --- | --- | --- | --- | --- | --- | --- | --- | --- | --- | --- | --- | --- |
| cg09635954 | 7 | PRR15 | rs62457133 | 29600423 | -0.51248 | 0.057995 | 4.93E-19 | T | C | 0.1543 | 0.076561 | 41.32977 |
| cg09639152 | 3 |  | rs78755722 | 45032222 | -1.381 | 0.122631 | 1.02E-29 | T | C | 0.03132 | 0.120593 | 136.8557 |
| cg09658497 | 7 | GNA12 | rs7790322 | 2830498 | -0.4862 | 0.041796 | 1.40E-31 | T | C | 0.4072 | 0.128058 | 146.5709 |
| cg09701700 | 10 | MIR146B | rs1536309 | 104195202 | -0.5096 | 0.043883 | 1.77E-31 | G | A | 0.3706 | 0.127651 | 146.037 |
| cg09735822 | 12 |  | rs11613876 | 133014486 | 0.3691 | 0.041066 | 1.26E-19 | G | A | 0.49 | 0.079056 | 85.6703 |
| cg09935388 | 1 | GFI1 | rs115427247 | 92878407 | -0.7447 | 0.1273 | 2.46E-09 | T | C | 0.02837 | 0.035032 | 36.23103 |
| cg09938479 | 1 |  | rs114168682 | 178456014 | -1.321 | 0.166651 | 1.12E-15 | T | C | 0.01773 | 0.06233 | 66.3407 |
| cg10012512 | 7 |  | rs11983032 | 157223543 | -0.3584 | 0.041842 | 5.38E-18 | G | A | 0.4498 | 0.072183 | 77.64295 |
| cg10130088 | 11 |  | rs77062901 | 122127334 | 0.3502 | 0.047129 | 5.40E-14 | A | G | 0.12 | 0.055139 | 58.23954 |
| cg10180092 | 3 | TNIK | rs902953 | 170988345 | -0.62849 | 0.039128 | 2.34E-58 | C | T | 0.4651 | 0.228949 | 148.0202 |
| cg10180092 | 3 | TNIK | rs7616996 | 170991408 | -0.26575 | 0.045963 | 3.70E-09 | T | C | 0.2695 | 0.034263 | 17.68624 |
| cg10255761 | 3 | KLHDC8B | rs4955427 | 49260646 | -0.3254 | 0.039 | 3.60E-17 | T | C | 0.2796 | 0.068685 | 73.60332 |
| cg10381071 | 15 | TLE3 | rs661046 | 70379247 | -0.5572 | 0.047005 | 1.03E-32 | G | T | 0.25 | 0.132587 | 152.5481 |
| cg10403394 | 15 | TPM1 | rs7170462 | 63345622 | 0.680648 | 0.04587 | 4.12E-50 | A | G | 0.2039 | 0.19916 | 123.9717 |
| cg10403394 | 15 | TPM1 | rs28645615 | 63348601 | -0.43499 | 0.053451 | 2.01E-16 | T | C | 0.1424 | 0.065519 | 34.95132 |
| cg10453071 | 16 | DOK4 | rs72788084 | 57512664 | 0.513861 | 0.091766 | 1.07E-08 | A | G | 0.05615 | 0.032253 | 16.61388 |
| cg10453071 | 16 | DOK4 | rs4784819 | 57571090 | 0.259819 | 0.045447 | 5.42E-09 | A | C | 0.4119 | 0.033541 | 17.30063 |
| cg10585661 | 3 | FAM131A | rs2178403 | 184039666 | -0.4816 | 0.053005 | 5.14E-20 | A | G | 0.2595 | 0.080689 | 43.75419 |
| cg10585661 | 3 | FAM131A | rs9845144 | 184056716 | 0.376791 | 0.04968 | 1.67E-14 | A | G | 0.2831 | 0.057323 | 30.31341 |
| cg10590964 | 2 |  | rs13397682 | 202948195 | 0.406192 | 0.040496 | 5.61E-24 | T | C | 0.2482 | 0.097178 | 53.65776 |
| cg10590964 | 2 |  | rs6722274 | 202927048 | -0.31644 | 0.039687 | 7.71E-16 | T | C | 0.2683 | 0.063028 | 33.53325 |
| cg10672416 | 12 | C12orf65 | rs1790104 | 123709409 | -0.3263 | 0.042132 | 4.79E-15 | C | T | 0.2884 | 0.059643 | 63.29939 |
| cg10696445 | 11 | TOLLIP | rs3793970 | 1318897 | 0.3494 | 0.045648 | 9.73E-15 | C | T | 0.2671 | 0.058328 | 61.81694 |
| cg10717312 | 2 | SNED1 | rs6437222 | 241920808 | -0.44603 | 0.074757 | 1.21E-09 | T | C | 0.08806 | 0.036363 | 18.81091 |
| cg10717312 | 2 | SNED1 | rs7560898 | 241977665 | -0.44914 | 0.043205 | 1.30E-25 | A | G | 0.4947 | 0.103906 | 57.80345 |
| cg10750959 | 6 | MAPK13 | rs851007 | 36064036 | 0.2356 | 0.042764 | 1.80E-08 | G | A | 0.4704 | 0.031278 | 32.22289 |
| cg10835286 | 2 |  | rs62143990 | 66659779 | -0.6746 | 0.040951 | 2.86E-61 | A | G | 0.3126 | 0.239208 | 313.7906 |
| cg10843276 | 4 | PCGF3 | rs5024302 | 725948 | -0.552 | 0.035889 | 1.10E-53 | A | G | 0.3853 | 0.212206 | 268.8282 |
| cg10843343 | 12 | KRT8 | rs10735847 | 53305606 | -0.3604 | 0.041969 | 4.45E-18 | C | G | 0.331 | 0.07253 | 78.04612 |
| cg10858677 | 15 |  | rs28802894 | 49362316 | 0.3513 | 0.049215 | 4.73E-13 | A | C | 0.2275 | 0.051091 | 53.73406 |
| cg10959726 | 17 | C17orf49 | rs3180517 | 6917850 | -0.3542 | 0.064069 | 1.62E-08 | A | C | 0.1442 | 0.031482 | 32.44079 |
| cg11014740 | 2 |  | rs12469358 | 129045584 | -0.368 | 0.05388 | 4.25E-12 | C | T | 0.1767 | 0.046987 | 49.20459 |
| cg11066209 | 7 | FTSJ2 | rs1108036 | 2263354 | 0.4287 | 0.042695 | 5.03E-24 | A | G | 0.3162 | 0.097372 | 107.6606 |
| cg11095027 | 11 | TOLLIP | rs55693520 | 1272800 | -1.167 | 0.138966 | 2.28E-17 | T | C | 0.02128 | 0.069532 | 74.5782 |
| cg11105358 | 5 |  | rs4397143 | 176777022 | -0.7217 | 0.040069 | 7.95E-73 | C | A | 0.3251 | 0.278593 | 385.4076 |
| cg11130692 | 8 | EIF2C2 | rs3735805 | 141577522 | -0.3162 | 0.049597 | 9.13E-11 | T | C | 0.1383 | 0.041231 | 42.91765 |
| cg11146034 | 12 | ELK3 | rs2300554 | 96617024 | 0.4123 | 0.046214 | 2.30E-19 | A | G | 0.3942 | 0.077956 | 84.37729 |

| cg11212234 | 5 |  | rs10040209 | 74353535 | 0.6928 | 0.049181 | 2.29E-45 | A | G | 0.2139 | 0.18151 | 221.3187 |
| --- | --- | --- | --- | --- | --- | --- | --- | --- | --- | --- | --- | --- |
| cg11229399 | 1 |  | rs1925714 | 228703289 | 0.6134 | 0.05342 | 8.07E-31 | A | G | 0.1726 | 0.125017 | 142.5931 |
| cg11305121 | 2 | RALB | rs62168280 | 121036385 | -0.2957 | 0.036986 | 6.49E-16 | T | G | 0.3387 | 0.063349 | 67.49878 |
| cg11323506 | 5 | RNU5E | rs17294956 | 80540556 | -0.3887 | 0.043097 | 9.47E-20 | G | C | 0.2488 | 0.079575 | 86.28146 |
| cg11405655 | 1 |  | rs3934834 | 1005806 | 0.4832 | 0.059087 | 1.45E-16 | T | C | 0.146 | 0.066124 | 70.66409 |
| cg11461808 | 7 | GPER | rs3808351 | 1126659 | -0.3538 | 0.040756 | 1.96E-18 | A | G | 0.3369 | 0.074033 | 79.7924 |
| cg11527913 | 1 | GALNT2 | rs12091838 | 230410162 | 0.4564 | 0.057302 | 8.27E-16 | T | C | 0.1501 | 0.062899 | 66.98651 |
| cg11530213 | 8 | PLAT | rs4615558 | 42006818 | 0.5664 | 0.034499 | 7.15E-61 | A | G | 0.4007 | 0.237813 | 311.3902 |
| cg11601297 | 19 | ITPKC | rs3745213 | 41248009 | 0.495 | 0.052584 | 2.40E-21 | T | C | 0.1353 | 0.086257 | 94.2113 |
| cg11660018 | 11 | PRSS23 | rs36061072 | 86493470 | -0.3525 | 0.051764 | 4.89E-12 | A | C | 0.2086 | 0.046723 | 48.91534 |
| cg11827514 | 8 |  | rs6993344 | 143126970 | 0.619 | 0.040742 | 1.96E-52 | T | C | 0.3948 | 0.207661 | 261.5614 |
| cg11902777 | 5 | AHRR | rs11749415 | 359522 | 0.461 | 0.074146 | 2.53E-10 | A | T | 0.1076 | 0.039316 | 40.84306 |
| cg12275060 | 1 | BTBD19 | rs932691 | 45323887 | 0.3564 | 0.048812 | 1.42E-13 | C | A | 0.3617 | 0.053334 | 56.22624 |
| cg12289251 | 10 | CACNB2 | rs76077446 | 18497253 | -0.7635 | 0.099703 | 9.46E-15 | A | T | 0.05024 | 0.05838 | 61.87593 |
| cg12421513 | 1 | RHD | rs2982358 | 25745204 | 0.3268 | 0.039084 | 3.10E-17 | G | T | 0.4551 | 0.068964 | 73.92467 |
| cg12504098 | 7 |  | rs2534599 | 38278883 | -0.32717 | 0.05035 | 4.07E-11 | C | G | 0.1956 | 0.042747 | 22.26105 |
| cg12504098 | 7 |  | rs2009434 | 38282166 | 0.313601 | 0.049446 | 1.13E-10 | G | T | 0.2069 | 0.040825 | 21.2175 |
| cg12616487 | 11 | ROM1 | rs12808829 | 62378660 | -0.27 | 0.039036 | 2.31E-12 | A | G | 0.3493 | 0.048126 | 50.45862 |
| cg12619504 | 5 | SQSTM1 | rs76360119 | 179183793 | 1.436 | 0.131278 | 3.77E-28 | A | G | 0.02128 | 0.114252 | 128.7318 |
| cg12803068 | 7 | MYO1G | rs61087358 | 45017743 | -0.4054 | 0.051127 | 1.10E-15 | A | G | 0.2098 | 0.062368 | 66.38337 |
| cg12828729 | 5 |  | rs6871223 | 134821336 | 0.652 | 0.071743 | 5.04E-20 | T | C | 0.09752 | 0.080725 | 87.63763 |
| cg13039251 | 5 | PDZD2 | rs62360842 | 31847718 | 0.5345 | 0.058417 | 2.85E-20 | G | A | 0.1217 | 0.081761 | 88.86333 |
| cg13057898 | 1 | LRRC47 | rs6667255 | 3711689 | 0.6304 | 0.045425 | 4.31E-44 | C | T | 0.2565 | 0.176707 | 214.2056 |
| cg13142374 | 3 | FLNB | rs9311663 | 58026373 | 0.4101 | 0.05221 | 2.00E-15 | C | T | 0.08511 | 0.061262 | 65.12895 |
| cg13185177 | 3 | GP5 | rs7434119 | 194166825 | 0.2837 | 0.041582 | 4.47E-12 | C | T | 0.1631 | 0.04689 | 49.09858 |
| cg13257421 | 2 | TAF1B | rs79642340 | 9985601 | -0.73387 | 0.058406 | 1.65E-36 | G | A | 0.07742 | 0.147549 | 86.28403 |
| cg13257421 | 2 | TAF1B | rs6432044 | 10041284 | 0.269469 | 0.032877 | 1.24E-16 | A | C | 0.4178 | 0.066407 | 35.4587 |
| cg13443605 | 17 | HS3ST3B1 | rs1052686 | 14207430 | -0.3337 | 0.05696 | 2.34E-09 | C | G | 0.2275 | 0.035128 | 36.3344 |
| cg13452162 | 13 | CLDN10 | rs946228 | 96133018 | 0.3167 | 0.041242 | 8.01E-15 | A | G | 0.4374 | 0.058689 | 62.22297 |
| cg13708645 | 12 | KDM2B | rs60370741 | 121966676 | 0.4698 | 0.040729 | 4.40E-31 | C | T | 0.4864 | 0.126071 | 143.9693 |
| cg13782866 | 1 | TSNAX-DI | rs58403453 | 231813438 | 0.5519 | 0.041708 | 2.85E-40 | T | C | 0.3392 | 0.162141 | 193.1313 |
| cg13809441 | 6 |  | rs1294416 | 6741327 | 0.4669 | 0.040874 | 1.61E-30 | G | A | 0.4923 | 0.123816 | 141.0298 |
| cg13985198 | 1 | SNORD46 | rs116807928 | 45282250 | 0.4979 | 0.053849 | 1.16E-20 | T | C | 0.1909 | 0.083395 | 90.80041 |
| cg13985437 | 11 | LRRC32 | rs4945097 | 76368553 | 0.2637 | 0.048188 | 2.22E-08 | G | A | 0.5 | 0.030882 | 31.80248 |
| cg14016875 | 17 | HS3ST3B1 | rs9904251 | 14193662 | -0.4516 | 0.046619 | 1.71E-22 | G | T | 0.2429 | 0.091033 | 99.9495 |
| cg14120896 | 19 |  | rs1053817 | 56089219 | 0.4682 | 0.068921 | 5.48E-12 | T | C | 0.1194 | 0.046508 | 48.67918 |
| cg14174221 | 21 | UBASH3A | rs35500161 | 43835492 | 0.4565 | 0.03222 | 7.22E-46 | C | T | 0.331 | 0.183394 | 224.131 |
| cg14194983 | 1 | NPPA | rs169158 | 11898228 | -1.017 | 0.060849 | 5.22E-63 | C | T | 0.1229 | 0.245265 | 324.3191 |

| cg14222656 | 22 |  | rs11704811 | 24833164 | -0.69 | 0.084743 | 1.94E-16 | T | C | 0.06974 | 0.06558 | 70.04269 |
| --- | --- | --- | --- | --- | --- | --- | --- | --- | --- | --- | --- | --- |
| cg14242895 | 11 | MLL | rs3132786 | 118365956 | 0.3279 | 0.033956 | 2.31E-22 | C | T | 0.1927 | 0.090494 | 99.29869 |
| cg14295611 | 9 |  | rs443876 | 136898922 | 0.2531 | 0.041808 | 7.07E-10 | G | T | 0.367 | 0.03738 | 38.7536 |
| cg14316231 | 8 | MYST3 | rs11786808 | 41893079 | -0.2249 | 0.040974 | 2.02E-08 | C | T | 0.3806 | 0.031058 | 31.98928 |
| cg14330293 | 17 | MYO1C | rs4411554 | 1319765 | -0.2346 | 0.043161 | 2.73E-08 | C | G | 0.2961 | 0.030492 | 31.3876 |
| cg14357089 | 10 | CACNB2 | rs73595550 | 18503690 | -0.8983 | 0.100595 | 2.13E-19 | C | T | 0.04846 | 0.078094 | 84.5393 |
| cg14391586 | 20 | SOX18 | rs816943 | 62676009 | 0.5708 | 0.039283 | 3.89E-48 | G | A | 0.4805 | 0.191859 | 236.9333 |
| cg14391923 | 8 |  | rs11781301 | 140586695 | -0.58056 | 0.042446 | 6.91E-43 | T | C | 0.2086 | 0.172139 | 103.6543 |
| cg14391923 | 8 |  | rs2615413 | 140590799 | 0.429273 | 0.037107 | 2.97E-31 | T | C | 0.3245 | 0.126753 | 72.35808 |
| cg14459011 | 4 | NHEDC2 | rs12500173 | 103997683 | 0.3748 | 0.064227 | 2.68E-09 | C | G | 0.1052 | 0.034869 | 36.05602 |
| cg14550518 | 3 | ZNF385D | rs7634827 | 21792882 | 0.3735 | 0.050952 | 1.15E-13 | A | G | 0.3227 | 0.053736 | 56.67402 |
| cg14602222 | 12 | RAD52 | rs79338718 | 986416 | -0.33423 | 0.056743 | 1.93E-09 | A | C | 0.1667 | 0.035489 | 18.34244 |
| cg14602222 | 12 | RAD52 | rs10744729 | 1024594 | 0.454951 | 0.043254 | 3.57E-26 | T | G | 0.4462 | 0.106204 | 59.23371 |
| cg14624381 | 9 |  | rs10988389 | 132218041 | 0.428 | 0.039924 | 4.08E-27 | T | C | 0.3487 | 0.110048 | 123.4089 |
| cg14656441 | 1 | NDUFS5 | rs12567986 | 39493618 | -0.4 | 0.037936 | 2.71E-26 | C | T | 0.3079 | 0.106694 | 119.1987 |
| cg14841514 | 10 | ZMIZ1 | rs946009 | 80864094 | -0.2559 | 0.042159 | 6.40E-10 | T | C | 0.4515 | 0.037567 | 38.9554 |
| cg14880079 | 3 | GP5 | rs1466733 | 194120998 | 0.2519 | 0.035922 | 1.17E-12 | G | A | 0.2411 | 0.049397 | 51.86033 |
| cg15029032 | 15 |  | rs3743326 | 86289879 | -0.91212 | 0.074585 | 1.09E-34 | C | G | 0.08806 | 0.140407 | 81.42563 |
| cg15029032 | 15 |  | rs11632436 | 86295286 | -0.30121 | 0.041468 | 1.88E-13 | G | C | 0.4953 | 0.052812 | 27.7946 |
| cg15059065 | 19 | NR2F6 | rs891202 | 17338033 | 0.298679 | 0.046908 | 9.62E-11 | T | C | 0.3777 | 0.041132 | 21.38396 |
| cg15059065 | 19 | NR2F6 | rs74182298 | 17346953 | -0.4563 | 0.046261 | 3.00E-23 | G | A | 0.3002 | 0.09417 | 51.82383 |
| cg15059804 | 1 | ZNF362 | rs4653002 | 33770526 | 0.5508 | 0.039463 | 1.42E-44 | C | T | 0.4261 | 0.178532 | 216.8986 |
| cg15084803 | 3 | CHCHD4 | rs2607742 | 14151478 | -0.7061 | 0.058839 | 1.77E-33 | A | G | 0.1336 | 0.135618 | 156.5827 |
| cg15089077 | 1 | TTC22 | rs10888879 | 55243590 | -1.342 | 0.122428 | 2.92E-28 | T | G | 0.03369 | 0.1147 | 129.301 |
| cg15122985 | 20 | TCEA2 | rs816956 | 62689037 | 0.3067 | 0.043507 | 8.98E-13 | G | C | 0.4238 | 0.049894 | 52.40889 |
| cg15130459 | 5 | SNORD72 | rs41271051 | 40834735 | -0.938 | 0.146467 | 7.56E-11 | C | T | 0.02128 | 0.041584 | 43.30181 |
| cg15233611 | 12 | SETD1B | rs1168666 | 122243905 | -0.2979 | 0.044992 | 1.78E-11 | C | T | 0.1832 | 0.044298 | 46.2582 |
| cg15331301 | 6 |  | rs12202737 | 150387746 | 0.2852 | 0.036727 | 4.07E-15 | T | C | 0.2796 | 0.059947 | 63.64258 |
| cg15339249 | 4 | DTHD1 | rs11724949 | 36299128 | 0.3242 | 0.049076 | 1.97E-11 | T | C | 0.2323 | 0.044106 | 46.04931 |
| cg15474579 | 6 | CDKN1A | rs762624 | 36645588 | -0.3829 | 0.040575 | 1.92E-21 | C | A | 0.2423 | 0.086659 | 94.69106 |
| cg15578140 | 7 | MIR548F3 | rs10266911 | 147715709 | -0.2726 | 0.041633 | 2.92E-11 | C | T | 0.3091 | 0.043369 | 45.24479 |
| cg15626881 | 7 |  | rs140931098 | 33916333 | 1.22 | 0.203636 | 1.04E-09 | C | G | 0.01241 | 0.036648 | 37.96657 |
| cg15693483 | 7 | C7orf50 | rs77868187 | 1093968 | 0.9989 | 0.045885 | 2.25E-105 | C | A | 0.1507 | 0.378978 | 609.0289 |
| cg15693572 | 3 |  | rs7649867 | 22401148 | -0.4145 | 0.04725 | 8.74E-19 | C | G | 0.3162 | 0.075514 | 81.51906 |
| cg15787744 | 6 | NFKBIE | rs730775 | 44232074 | 0.3899 | 0.03599 | 1.19E-27 | G | A | 0.4161 | 0.11222 | 126.1519 |
| cg15903032 | 10 |  | rs4590800 | 101301093 | 0.3167 | 0.051298 | 3.33E-10 | A | C | 0.2547 | 0.038793 | 40.27838 |
| cg15937073 | 1 | HIVEP3 | rs783629 | 42363275 | -0.6416 | 0.038173 | 1.07E-63 | C | T | 0.4309 | 0.247652 | 328.514 |
| cg15950273 | 14 | TRAF3 | rs59312719 | 103309391 | 1.03 | 0.116139 | 3.70E-19 | C | T | 0.02955 | 0.077087 | 83.35884 |

| cg15951188 | 17 | KCNAB3 | rs4444395 | 7828275 | -0.4728 | 0.044811 | 2.51E-26 | G | A | 0.4628 | 0.106824 | 119.3615 |
| --- | --- | --- | --- | --- | --- | --- | --- | --- | --- | --- | --- | --- |
| cg15955046 | 2 | COLEC11 | rs11123637 | 3654190 | -0.56475 | 0.039298 | 3.95E-47 | T | C | 0.3623 | 0.188116 | 115.5036 |
| cg15985905 | 12 |  | rs905409 | 128079959 | 0.2365 | 0.033139 | 4.79E-13 | C | A | 0.2358 | 0.05107 | 53.71085 |
| cg16037981 | 17 | RAP1GAP2 | rs178567 | 2699322 | 0.3294 | 0.046965 | 1.16E-12 | T | C | 0.328 | 0.049415 | 51.87946 |
| cg16119613 | 12 |  | rs6489164 | 127788585 | -0.56478 | 0.089132 | 1.18E-10 | T | C | 0.05792 | 0.040754 | 21.17876 |
| cg16119613 | 12 |  | rs750278 | 127760579 | -0.29851 | 0.045915 | 3.98E-11 | G | A | 0.2736 | 0.04279 | 22.28439 |
| cg16274678 | 1 | TPM3 | rs2494667 | 154109153 | 0.435639 | 0.043235 | 3.52E-24 | A | G | 0.487 | 0.098012 | 54.16836 |
| cg16274678 | 1 | TPM3 | rs6676022 | 154162503 | -0.42788 | 0.071117 | 8.91E-10 | T | C | 0.1099 | 0.036943 | 19.1227 |
| cg16416158 | 1 | ECE1 | rs213030 | 21653847 | 0.5193 | 0.036748 | 1.22E-45 | T | C | 0.2683 | 0.182546 | 222.8632 |
| cg16516405 | 1 |  | rs76374387 | 225655151 | -0.8471 | 0.052574 | 1.04E-58 | A | G | 0.08688 | 0.230191 | 298.4259 |
| cg16554099 | 6 |  | rs554785 | 7036609 | 0.277322 | 0.042501 | 3.40E-11 | C | T | 0.25 | 0.043085 | 22.4451 |
| cg16554099 | 6 |  | rs7769136 | 7052225 | 0.46943 | 0.052112 | 1.05E-19 | T | C | 0.1413 | 0.079391 | 42.98955 |
| cg16767506 | 7 |  | rs1969595 | 142468189 | -0.3605 | 0.035361 | 1.05E-24 | G | C | 0.4273 | 0.100184 | 111.1151 |
| cg16786458 | 5 | PPARGC1B | rs17797688 | 149114148 | -0.41101 | 0.058809 | 1.39E-12 | A | G | 0.1182 | 0.049083 | 25.73079 |
| cg16786458 | 5 | PPARGC1B | rs17711318 | 149125721 | 0.633414 | 0.054019 | 4.70E-32 | G | C | 0.1371 | 0.129954 | 74.45839 |
| cg16822035 | 13 | MCF2L | rs2993280 | 113633193 | -0.2481 | 0.036346 | 4.37E-12 | G | C | 0.3895 | 0.046935 | 49.14774 |
| cg16936953 | 17 | TMEM49 | rs8068913 | 57973096 | -0.3425 | 0.041893 | 1.47E-16 | T | C | 0.2311 | 0.06609 | 70.626 |
| cg16983588 | 11 | PRDM10 | rs7924347 | 129808408 | -0.2657 | 0.045332 | 2.30E-09 | G | A | 0.4675 | 0.035159 | 36.36728 |
| cg17009069 | 2 | ITM2C | rs4972973 | 231737798 | 0.2575 | 0.034871 | 7.66E-14 | A | G | 0.4699 | 0.05449 | 57.51455 |
| cg17098103 | 1 | HIVEP3 | rs1004870 | 42370787 | -0.3526 | 0.038885 | 6.07E-20 | C | T | 0.4113 | 0.080386 | 87.23782 |
| cg17127702 | 1 | PARP1 | rs76887998 | 226539353 | 0.952803 | 0.042808 | 4.77E-110 | C | T | 0.1643 | 0.392201 | 321.6718 |
| cg17127702 | 1 | PARP1 | rs6681537 | 226609436 | -0.24229 | 0.04343 | 1.21E-08 | T | C | 0.1613 | 0.032026 | 16.49294 |
| cg17390562 | 6 | EZR | rs9346767 | 159197371 | 0.3397 | 0.034773 | 7.65E-23 | T | C | 0.4941 | 0.092483 | 101.7037 |
| cg17449254 | 21 | C21orf121 | rs58417802 | 43443113 | 0.5888 | 0.082935 | 6.26E-13 | C | A | 0.06797 | 0.050569 | 53.15626 |
| cg17551891 | 7 | MAD1L1 | rs4721164 | 1932629 | -0.43448 | 0.049108 | 4.47E-19 | G | C | 0.2204 | 0.076741 | 41.435 |
| cg17551891 | 7 | MAD1L1 | rs56770872 | 1913784 | -0.50628 | 0.094795 | 4.63E-08 | A | G | 0.05319 | 0.029498 | 15.15159 |
| cg17580614 | 17 | ADORA2B | rs11651258 | 15736473 | -0.649 | 0.03624 | 5.07E-72 | C | T | 0.2849 | 0.275918 | 380.2966 |
| cg17586094 | 2 |  | rs772787 | 235239488 | 0.4537 | 0.041898 | 1.26E-27 | A | G | 0.3404 | 0.112126 | 126.0334 |
| cg17823346 | 10 | ZMIZ1 | rs12359051 | 80845405 | -0.5906 | 0.029963 | 8.73E-87 | G | A | 0.3233 | 0.323482 | 477.2003 |
| cg17884674 | 19 | CSNK1G2 | rs11673037 | 1955524 | 0.463 | 0.035057 | 3.99E-40 | C | T | 0.263 | 0.161577 | 192.3299 |
| cg17924476 | 5 | AHRR | rs6871034 | 256109 | 0.3685 | 0.046204 | 7.58E-16 | G | C | 0.4675 | 0.06306 | 67.1696 |
| cg17927313 | 15 | PLEKHO2 | rs113818297 | 65127504 | -0.3152 | 0.038852 | 2.47E-16 | G | A | 0.3582 | 0.065132 | 69.53048 |
| cg18033416 | 17 | RHBDL3 | rs12453610 | 30595839 | 0.2459 | 0.038276 | 6.62E-11 | C | T | 0.4557 | 0.041834 | 43.57313 |
| cg18132076 | 6 | GMDS | rs9503060 | 1986607 | 0.3796 | 0.035937 | 2.21E-26 | T | C | 0.3895 | 0.107053 | 119.6475 |
| cg18146737 | 1 | GFI1 | rs115427247 | 92878407 | -0.9259 | 0.139936 | 1.84E-11 | T | C | 0.02837 | 0.04424 | 46.19516 |
| cg18151030 | 21 | PRDM15 | rs2236691 | 43220178 | 0.4029 | 0.04472 | 1.04E-19 | C | T | 0.2275 | 0.079413 | 86.09077 |
| cg18262201 | 10 | PFKFB3 | rs61839726 | 6185344 | -0.50199 | 0.058474 | 4.55E-18 | A | G | 0.1448 | 0.07249 | 38.96075 |
| cg18369034 | 14 | PTGDR | rs11623990 | 52733137 | -0.36845 | 0.054291 | 5.74E-12 | A | C | 0.1584 | 0.046421 | 24.26742 |

| cg18369034 | 14 | PTGDR | rs4901266 | 52749004 | -0.30142 | 0.039708 | 1.59E-14 | A | G | 0.4131 | 0.057418 | 30.36662 |
| --- | --- | --- | --- | --- | --- | --- | --- | --- | --- | --- | --- | --- |
| cg18432895 | 8 | DUSP4 | rs12540995 | 29197529 | -0.6039 | 0.04625 | 2.89E-39 | T | C | 0.2329 | 0.158263 | 187.6438 |
| cg18446336 | 7 | GNA12 | rs2644307 | 2832973 | -0.3831 | 0.04217 | 5.20E-20 | A | G | 0.4096 | 0.080668 | 87.57118 |
| cg18503679 | 3 | ZNF385D | rs7634827 | 21792882 | 0.3418 | 0.049929 | 3.80E-12 | A | G | 0.3227 | 0.047192 | 49.43081 |
| cg18533225 | 22 | KLHDC7B | rs6009988 | 50985875 | 0.5819 | 0.048704 | 3.33E-33 | C | T | 0.1986 | 0.134525 | 155.1239 |
| cg18584067 | 2 |  | rs1107436 | 64975919 | 0.3096 | 0.044179 | 1.21E-12 | C | T | 0.3103 | 0.049336 | 51.79279 |
| cg18585107 | 11 | RPS6KA4 | rs538147 | 64129722 | 0.329186 | 0.039564 | 4.39E-17 | A | G | 0.4019 | 0.068324 | 36.55699 |
| cg18585107 | 11 | RPS6KA4 | rs7936860 | 64161278 | -0.26573 | 0.039395 | 7.63E-12 | G | A | 0.4255 | 0.045888 | 23.97537 |
| cg18617091 | 22 | TMPRSS6 | rs733655 | 37495051 | 0.5952 | 0.036912 | 8.53E-59 | C | T | 0.2429 | 0.230499 | 298.9442 |
| cg18708252 | 22 | CBX7 | rs710190 | 39527790 | -0.5768 | 0.036983 | 3.86E-55 | C | T | 0.4604 | 0.217458 | 277.331 |
| cg18810691 | 7 | CRYGN | rs12539860 | 151135503 | -0.6194 | 0.038814 | 1.25E-57 | T | C | 0.3286 | 0.226368 | 292.0185 |
| cg18824446 | 15 | MIR7-2 | rs6496492 | 89056941 | 0.6332 | 0.039521 | 4.49E-58 | T | C | 0.3398 | 0.227943 | 294.6514 |
| cg18919541 | 12 |  | rs71454676 | 121529227 | 0.748331 | 0.080835 | 1.05E-20 | G | A | 0.06856 | 0.083585 | 45.46737 |
| cg18919541 | 12 |  | rs73216105 | 121519921 | -0.55393 | 0.073499 | 2.41E-14 | T | C | 0.0857 | 0.05664 | 29.93023 |
| cg18946533 | 15 | SH2D7 | rs12910083 | 78384669 | 0.6504 | 0.105573 | 3.62E-10 | T | C | 0.03901 | 0.038638 | 40.11027 |
| cg19022697 | 1 | TTC22 | rs10888879 | 55243590 | -0.992 | 0.128851 | 6.87E-15 | T | G | 0.03369 | 0.058976 | 62.54647 |
| cg19030554 | 16 | NME3 | rs8055552 | 1828839 | -0.3234 | 0.050129 | 5.54E-11 | G | A | 0.2193 | 0.042167 | 21.94559 |
| cg19030554 | 16 | NME3 | rs2575329 | 1819433 | -0.59607 | 0.043311 | 2.14E-43 | G | A | 0.4054 | 0.17407 | 105.0618 |
| cg19197419 | 20 | UBE2C | rs6032535 | 44417334 | 0.3501 | 0.044388 | 1.54E-15 | T | C | 0.3298 | 0.061743 | 65.67389 |
| cg19406367 | 1 | SGIP1 | rs11811067 | 67205722 | 0.4031 | 0.044812 | 1.18E-19 | C | T | 0.2866 | 0.079179 | 85.81525 |
| cg19452802 | 6 |  | rs10807342 | 46451316 | 0.3278 | 0.047502 | 2.59E-12 | T | C | 0.3375 | 0.047914 | 50.22492 |
| cg19696491 | 15 | CHRNA5 | rs76712448 | 78836719 | 0.3764 | 0.040173 | 3.65E-21 | A | G | 0.461 | 0.085498 | 93.3041 |
| cg19717773 | 7 | GNA12 | rs2644307 | 2832973 | -0.37674 | 0.04866 | 4.88E-15 | A | G | 0.4096 | 0.05961 | 31.59948 |
| cg19717773 | 7 | GNA12 | rs1182197 | 2863289 | -0.28518 | 0.050459 | 7.94E-09 | C | A | 0.3723 | 0.032821 | 16.91673 |
| cg19757176 | 1 | TPM3 | rs4400599 | 154127100 | 0.5086 | 0.040339 | 9.54E-37 | T | C | 0.4817 | 0.148476 | 174.0168 |
| cg19758448 | 17 | PGAP3 | rs1565920 | 37831613 | -0.5634 | 0.043725 | 2.73E-38 | G | A | 0.3168 | 0.154484 | 182.3446 |
| cg19802390 | 1 |  | rs9782931 | 2841713 | 0.5113 | 0.049753 | 4.48E-25 | A | T | 0.234 | 0.101697 | 112.9837 |
| cg19804488 | 17 | GALK1 | rs60700976 | 73775585 | -0.4689 | 0.05542 | 1.33E-17 | A | G | 0.1353 | 0.070524 | 75.72359 |
| cg19872095 | 1 | ZC3H12A | rs115755325 | 37932644 | -0.7443 | 0.117815 | 1.33E-10 | A | G | 0.01182 | 0.040524 | 42.15085 |
| cg19935065 | 10 | DNTT | rs10748673 | 98122352 | -0.3343 | 0.056413 | 1.55E-09 | A | T | 0.2329 | 0.035898 | 37.16013 |
| cg19956914 | 7 | SUMF2 | rs10242503 | 56243419 | -0.3255 | 0.048492 | 9.57E-12 | A | G | 0.2547 | 0.045464 | 47.53436 |
| cg20059377 | 15 | TBC1D2B | rs72732535 | 78285918 | 0.8137 | 0.040115 | 8.86E-92 | T | G | 0.2429 | 0.338857 | 511.5065 |
| cg20124610 | 13 | CARS2 | rs7330080 | 111367115 | 0.3257 | 0.04365 | 4.27E-14 | C | T | 0.2518 | 0.055577 | 58.72997 |
| cg20322193 | 7 | RALA | rs76408259 | 39664721 | -0.4217 | 0.06482 | 3.87E-11 | G | A | 0.1572 | 0.042844 | 44.67239 |
| cg20375836 | 13 | STK24 | rs4411372 | 99130423 | 0.3183 | 0.046483 | 3.75E-12 | C | T | 0.2991 | 0.047218 | 49.45863 |
| cg20379671 | 22 |  | rs5757372 | 39320602 | 0.6414 | 0.04967 | 1.89E-38 | T | C | 0.185 | 0.155101 | 183.2069 |
| cg20451986 | 11 |  | rs80235995 | 133928704 | -0.5093 | 0.057544 | 4.35E-19 | T | C | 0.1082 | 0.076791 | 83.01162 |
| cg20469837 | 2 | GALNT5 | rs11899353 | 158111397 | 0.5461 | 0.061464 | 3.20E-19 | A | T | 0.1099 | 0.077354 | 83.67153 |

| cg20533899 | 11 | LRRC32 | rs12718488 | 76366690 | 0.3066 | 0.045311 | 6.59E-12 | G | T | 0.3611 | 0.046162 | 48.29947 |
| --- | --- | --- | --- | --- | --- | --- | --- | --- | --- | --- | --- | --- |
| cg20698421 | 2 | SLC1A4 | rs6546118 | 65212178 | 0.710608 | 0.055936 | 2.81E-37 | G | A | 0.159 | 0.150549 | 88.34948 |
| cg20698421 | 2 | SLC1A4 | rs759456 | 65194302 | -0.31918 | 0.042929 | 5.23E-14 | G | T | 0.3729 | 0.0552 | 29.12471 |
| cg20853880 | 2 | KLF11 | rs6747078 | 10177158 | -0.40311 | 0.051295 | 1.94E-15 | G | A | 0.2589 | 0.061317 | 32.56328 |
| cg20912205 | 3 | NAT6 | rs35926495 | 50255663 | -0.3486 | 0.044383 | 2.01E-15 | T | C | 0.3859 | 0.061256 | 65.12256 |
| cg21187770 | 2 | KIF3C | rs11126321 | 26203016 | -0.7451 | 0.075622 | 3.33E-23 | A | G | 0.07683 | 0.093981 | 103.5225 |
| cg21201401 | 20 | LIME1 | rs1291206 | 62329169 | 0.238 | 0.035197 | 6.81E-12 | A | G | 0.227 | 0.046102 | 48.2339 |
| cg21201657 | 19 | SAFB | rs1890098 | 5709412 | 0.3381 | 0.03932 | 4.03E-18 | T | C | 0.3174 | 0.072713 | 78.25762 |
| cg21307484 | 22 | IL2RB | rs3218258 | 37544245 | -0.3403 | 0.025794 | 4.81E-40 | A | G | 0.2719 | 0.161262 | 191.883 |
| cg21356710 | 2 | MFSD2B | rs925229 | 24234440 | 0.4381 | 0.040341 | 8.94E-28 | G | A | 0.4374 | 0.11273 | 126.7987 |
| cg21664281 | 3 |  | rs1697343 | 193560865 | 0.8087 | 0.045369 | 2.26E-71 | A | G | 0.1779 | 0.273749 | 376.1804 |
| cg21717508 | 11 | SNX15 | rs616124 | 64777041 | -0.2092 | 0.037028 | 8.03E-09 | A | C | 0.3901 | 0.032801 | 33.84524 |
| cg21733098 | 12 |  | rs1622912 | 127931111 | -0.35728 | 0.04406 | 2.55E-16 | C | T | 0.49 | 0.065073 | 34.6965 |
| cg21791252 | 6 |  | rs3800341 | 33864998 | 0.4599 | 0.047836 | 3.48E-22 | G | A | 0.2015 | 0.089748 | 98.39963 |
| cg21869609 | 19 | LINGO3 | rs57678395 | 2350114 | 0.7245 | 0.038661 | 1.17E-78 | C | T | 0.4054 | 0.297692 | 423.0292 |
| cg21913886 | 1 | TMEM51 | rs61782688 | 15475675 | -0.6652 | 0.063374 | 4.48E-26 | C | G | 0.1424 | 0.105798 | 118.0784 |
| cg21929761 | 1 |  | rs78483830 | 32173659 | -0.9773 | 0.079527 | 5.20E-35 | G | T | 0.04078 | 0.141668 | 164.7197 |
| cg21990700 | 12 | LOC28331 | rs3782925 | 7262024 | 0.3169 | 0.04411 | 3.38E-13 | T | C | 0.331 | 0.051722 | 54.43345 |
| cg22041417 | 19 | TPM4 | rs36048257 | 16178250 | -0.551 | 0.033069 | 1.24E-62 | A | G | 0.3582 | 0.243963 | 322.0418 |
| cg22053945 | 17 | HOXB3 | rs7405452 | 46674670 | -0.5076 | 0.071742 | 7.46E-13 | T | C | 0.08215 | 0.050242 | 52.79373 |
| cg22132788 | 7 | MYO1G | rs61087358 | 45017743 | -0.4352 | 0.052252 | 4.08E-17 | A | G | 0.2098 | 0.068455 | 73.33869 |
| cg22158051 | 5 |  | rs325241 | 143172039 | -0.633 | 0.06112 | 1.95E-25 | A | G | 0.1312 | 0.103181 | 114.822 |
| cg22327175 | 11 | SORL1 | rs578506 | 121323477 | 0.283 | 0.04258 | 1.50E-11 | G | C | 0.4965 | 0.044619 | 46.60894 |
| cg22563815 | 15 | CHRNA5 | rs76712448 | 78836719 | 0.3536 | 0.036377 | 1.23E-22 | A | G | 0.461 | 0.091624 | 100.6636 |
| cg22586569 | 7 | FBXL18 | rs4724704 | 5541657 | -0.516 | 0.038922 | 2.04E-40 | G | A | 0.3788 | 0.162694 | 193.9184 |
| cg22642495 | 19 | C19orf66 | rs34484805 | 10223427 | -0.242 | 0.042372 | 5.61E-09 | G | A | 0.4616 | 0.033478 | 34.5682 |
| cg22650458 | 7 | C7orf41 | rs66691214 | 30188804 | 0.5385 | 0.037675 | 1.21E-46 | T | G | 0.3706 | 0.186306 | 228.5046 |
| cg22678092 | 1 | FCRLB | rs905592 | 161691922 | -0.6202 | 0.05027 | 2.85E-35 | T | C | 0.2128 | 0.142696 | 166.1149 |
| cg22789605 | 12 | SLC11A2 | rs440601 | 51403891 | -0.3251 | 0.049368 | 2.27E-11 | G | A | 0.2577 | 0.043843 | 45.76155 |
| cg22858500 | 10 | CACNB2 | rs74661789 | 18498027 | -0.8765 | 0.08856 | 2.14E-23 | C | T | 0.04905 | 0.094775 | 104.4883 |
| cg22871253 | 6 | EZR | rs9456354 | 159248864 | 0.295 | 0.038103 | 4.89E-15 | C | G | 0.4988 | 0.059607 | 63.25866 |
| cg22905866 | 4 |  | rs7670160 | 185462486 | 0.36635 | 0.045569 | 4.51E-16 | C | T | 0.3245 | 0.06402 | 34.0968 |
| cg22905866 | 4 |  | rs6823464 | 185449890 | 1.02259 | 0.10159 | 3.91E-24 | C | A | 0.0461 | 0.097825 | 54.05374 |
| cg23126342 | 13 | PCDH9 | rs61959555 | 67972681 | 0.31756 | 0.048565 | 3.10E-11 | A | G | 0.247 | 0.04326 | 22.53997 |
| cg23161492 | 15 | ANPEP | rs11853650 | 90358635 | -0.49163 | 0.038697 | 2.78E-37 | C | G | 0.4882 | 0.150563 | 88.359 |
| cg23161492 | 15 | ANPEP | rs111889606 | 90448701 | 0.750049 | 0.119731 | 1.87E-10 | G | C | 0.02955 | 0.039881 | 20.70634 |
| cg23222488 | 5 |  | rs35582636 | 176759778 | 0.2381 | 0.043256 | 1.85E-08 | A | C | 0.26 | 0.031226 | 32.16764 |
| cg23395310 | 9 | SNHG7 | rs3739939 | 139616840 | -0.27285 | 0.03831 | 5.31E-13 | G | A | 0.3895 | 0.050876 | 26.72088 |

| cg23395310 | 9 | SNHG7 | rs113982899 | 139622748 | 0.694795 | 0.048505 | 7.74E-47 | C | G | 0.1661 | 0.187024 | 114.6792 |
| --- | --- | --- | --- | --- | --- | --- | --- | --- | --- | --- | --- | --- |
| cg23681440 | 13 |  | rs2901312 | 27502147 | 0.5067 | 0.083974 | 8.00E-10 | G | A | 0.08333 | 0.037147 | 38.50342 |
| cg23716141 | 17 | 9-Sep | rs117078461 | 75360041 | 0.615039 | 0.071015 | 2.34E-18 | G | C | 0.06619 | 0.073707 | 39.66645 |
| cg23716141 | 17 | 9-Sep | rs2574846 | 75348110 | 0.439531 | 0.036698 | 2.35E-33 | C | T | 0.4657 | 0.13513 | 77.88715 |
| cg23762517 | 1 | HIVEP3 | rs783629 | 42363275 | -0.7329 | 0.038828 | 9.07E-80 | C | T | 0.4309 | 0.301277 | 430.3193 |
| cg23771366 | 11 | PRSS23 | rs67939314 | 86492304 | -0.4652 | 0.071545 | 3.96E-11 | G | T | 0.12 | 0.042801 | 44.62499 |
| cg23813257 | 16 | IL32 | rs13335800 | 3112197 | 0.2662 | 0.032165 | 6.36E-17 | A | T | 0.3452 | 0.067638 | 72.39946 |
| cg23884241 | 7 | HOXA4 | rs10225507 | 27170583 | 0.6038 | 0.05155 | 5.47E-32 | C | T | 0.198 | 0.12969 | 148.7177 |
| cg23940612 | 2 |  | rs3181381 | 218923606 | 0.5376 | 0.036934 | 2.68E-48 | A | G | 0.3995 | 0.192461 | 237.8535 |
| cg23975840 | 12 |  | rs7960700 | 117039310 | 0.3694 | 0.039514 | 4.44E-21 | G | A | 0.1897 | 0.085143 | 92.88051 |
| cg24033122 | 16 | ITGAL | rs11150589 | 30482494 | -0.7245 | 0.037499 | 1.80E-83 | T | C | 0.4864 | 0.313079 | 454.8604 |
| cg24049493 | 1 | HIVEP3 | rs2147904 | 42371414 | -0.3866 | 0.042414 | 3.94E-20 | T | C | 0.4362 | 0.081176 | 88.17046 |
| cg24249791 | 7 |  | rs17162969 | 142009774 | 1.254 | 0.066101 | 1.48E-80 | T | C | 0.0857 | 0.303803 | 435.5014 |
| cg24361098 | 2 | BCL11A | rs168562 | 60747192 | -0.488 | 0.036003 | 3.72E-42 | T | C | 0.2961 | 0.169353 | 203.4729 |
| cg24426691 | 13 | CLDN10 | rs946229 | 96132887 | 0.5259 | 0.052039 | 2.60E-24 | A | G | 0.1779 | 0.098555 | 109.1109 |
| cg24448421 | 12 |  | rs28445463 | 68733835 | -0.6385 | 0.052041 | 6.64E-35 | A | G | 0.1566 | 0.141249 | 164.1523 |
| cg24490227 | 11 |  | rs11601844 | 133932785 | -0.35835 | 0.063366 | 7.78E-09 | G | C | 0.1117 | 0.032861 | 16.93756 |
| cg24490227 | 11 |  | rs12795628 | 133925600 | -0.39013 | 0.04635 | 1.93E-17 | T | C | 0.2199 | 0.069834 | 37.42579 |
| cg24497361 | 11 | RHOG | rs4597058 | 3855859 | -0.3078 | 0.041869 | 9.80E-14 | C | G | 0.3842 | 0.054029 | 57.00027 |
| cg24503796 | 10 | SCD | rs1502593 | 102109202 | 0.4548 | 0.041571 | 3.69E-28 | A | G | 0.4019 | 0.114287 | 128.7759 |
| cg24504361 | 12 | KRT8 | rs10735847 | 53305606 | -0.32159 | 0.039416 | 1.69E-16 | C | G | 0.331 | 0.065834 | 35.13118 |
| cg24504361 | 12 | KRT8 | rs118151492 | 53270887 | 0.619375 | 0.093868 | 2.08E-11 | C | T | 0.04492 | 0.044008 | 22.94815 |
| cg24539517 | 10 | GRK5 | rs10886475 | 121161224 | 0.5671 | 0.04418 | 5.15E-38 | A | G | 0.266 | 0.153414 | 180.8527 |
| cg24647428 | 3 |  | rs7374806 | 100785426 | 0.6645 | 0.035206 | 9.22E-80 | G | C | 0.4947 | 0.301254 | 430.2735 |
| cg24718197 | 17 |  | rs12937453 | 79497036 | -0.4365 | 0.053928 | 2.88E-16 | A | C | 0.1785 | 0.064849 | 69.2071 |
| cg24924577 | 15 | SEMA4B | rs35089261 | 90753234 | 0.4912 | 0.045776 | 3.66E-27 | T | G | 0.1348 | 0.110241 | 123.6514 |
| cg25064552 | 1 | SLC39A1 | rs11264763 | 153948705 | -0.3232 | 0.035646 | 6.11E-20 | T | C | 0.2949 | 0.080373 | 87.22252 |
| cg25189904 | 1 | GNG12 | rs7540541 | 68299552 | 0.3532 | 0.047487 | 5.12E-14 | G | T | 0.3777 | 0.05524 | 58.35246 |
| cg25284397 | 7 | CDK6 | rs113181986 | 92266380 | 0.5931 | 0.095243 | 2.37E-10 | C | G | 0.05201 | 0.039433 | 40.96976 |
| cg25313468 | 4 | REST | rs55762216 | 57765731 | -0.40969 | 0.059889 | 3.94E-12 | C | G | 0.1944 | 0.047129 | 24.65576 |
| cg25313468 | 4 | REST | rs781669 | 57819794 | 0.291431 | 0.04602 | 1.20E-10 | C | T | 0.4787 | 0.040709 | 21.15452 |
| cg25347941 | 22 | TTLL1 | rs134994 | 43487047 | 0.322436 | 0.042323 | 1.28E-14 | A | G | 0.2441 | 0.057812 | 30.58787 |
| cg25607920 | 1 | HIVEP3 | rs2786487 | 42368339 | -0.6738 | 0.039147 | 1.08E-66 | G | C | 0.4309 | 0.257953 | 346.9275 |
| cg25625514 | 11 |  | rs1149577 | 76545414 | -0.7066 | 0.049411 | 1.09E-46 | C | T | 0.2145 | 0.186476 | 228.7621 |
| cg25649826 | 17 | USP22 | rs4985973 | 20970687 | -0.2364 | 0.029806 | 1.08E-15 | G | C | 0.1909 | 0.062398 | 66.41803 |
| cg25684105 | 12 | TXNRD1 | rs4964635 | 104587838 | -0.61 | 0.053785 | 4.09E-30 | T | A | 0.1283 | 0.122186 | 138.9145 |
| cg25741533 | 19 |  | rs17271883 | 5834212 | 0.4178 | 0.047226 | 4.50E-19 | T | C | 0.2979 | 0.076729 | 82.93954 |
| cg25809905 | 17 | ITGA2B | rs62081212 | 42468139 | 1.007 | 0.082849 | 2.71E-34 | A | G | 0.05496 | 0.138839 | 160.9 |

| cg25839482 | 15 | IMP3 | rs8035987 | 75750383 | 0.559 | 0.045196 | 1.94E-35 | C | T | 0.2417 | 0.143352 | 167.0063 |
| --- | --- | --- | --- | --- | --- | --- | --- | --- | --- | --- | --- | --- |
| cg26038582 | 1 | HIVEP3 | rs783629 | 42363275 | -0.6706 | 0.037929 | 2.96E-70 | C | T | 0.4309 | 0.270009 | 369.1409 |
| cg26055950 | 1 | TP73 | rs1122723 | 3594105 | -0.3901 | 0.039938 | 7.75E-23 | G | A | 0.432 | 0.09246 | 101.6756 |
| cg26076054 | 5 | AHRR | rs72711366 | 418093 | -0.54809 | 0.062925 | 1.52E-18 | G | A | 0.1436 | 0.074505 | 40.13073 |
| cg26102435 | 14 |  | rs34881810 | 99557138 | -0.4767 | 0.0493 | 2.03E-22 | C | T | 0.2092 | 0.09072 | 99.57215 |
| cg26126879 | 8 |  | rs548488 | 103909612 | -0.6966 | 0.033485 | 2.02E-96 | T | A | 0.2831 | 0.352841 | 544.1244 |
| cg26132737 | 2 | ANKRD44 | rs72926656 | 198029428 | -0.3771 | 0.061195 | 3.58E-10 | C | T | 0.1507 | 0.038658 | 40.13166 |
| cg26146569 | 15 | KLF13 | rs28510484 | 31637569 | -0.4132 | 0.032916 | 1.91E-36 | C | G | 0.172 | 0.147297 | 172.396 |
| cg26161820 | 17 | STARD3 | rs33938012 | 37785200 | -0.6517 | 0.08472 | 7.22E-15 | G | T | 0.03487 | 0.058882 | 62.44124 |
| cg26253500 | 7 | CHRM2 | rs1364407 | 136609925 | 0.3309 | 0.030051 | 1.69E-28 | C | T | 0.331 | 0.115665 | 130.532 |
| cg26351966 | 10 | SCD | rs2275656 | 102106205 | 0.38897 | 0.057037 | 4.57E-12 | C | G | 0.3859 | 0.046851 | 24.50301 |
| cg26351966 | 10 | SCD | rs670213 | 102106367 | 0.345219 | 0.056141 | 3.90E-10 | C | T | 0.497 | 0.038501 | 19.96136 |
| cg26403843 | 5 | RNF145 | rs10057691 | 158617525 | 0.539653 | 0.042108 | 6.67E-38 | A | T | 0.224 | 0.152978 | 90.03263 |
| cg26403843 | 5 | RNF145 | rs11135052 | 158598381 | -0.27605 | 0.04589 | 8.97E-10 | T | C | 0.1708 | 0.03693 | 19.11568 |
| cg26574777 | 13 | PCCA | rs9557411 | 100950994 | -0.3742 | 0.039692 | 2.10E-21 | T | G | 0.4994 | 0.086502 | 94.50327 |
| cg26585644 | 16 | DCI | rs55985840 | 2301272 | -0.3865 | 0.056701 | 4.66E-12 | T | G | 0.1566 | 0.046811 | 49.0115 |
| cg26599989 | 11 | TOLLIP | rs55693520 | 1272800 | -1.004 | 0.149045 | 8.13E-12 | T | C | 0.02128 | 0.04577 | 47.86946 |
| cg26703507 | 2 | SLC20A1 | rs6542074 | 113429752 | -0.406 | 0.035888 | 5.66E-30 | C | T | 0.4669 | 0.121618 | 138.1794 |
| cg26718213 | 2 | SNED1 | rs7560898 | 241977665 | -0.3951 | 0.044467 | 3.19E-19 | A | G | 0.4947 | 0.077359 | 83.67746 |
| cg26728709 | 4 | ARSJ | rs4452507 | 114822605 | 0.5543 | 0.040159 | 1.23E-43 | A | C | 0.2441 | 0.174989 | 211.6807 |
| cg26900995 | 17 | CYTSB | rs6587211 | 19949548 | 0.3852 | 0.062006 | 2.61E-10 | G | A | 0.1448 | 0.039255 | 40.77687 |
| cg26908328 | 5 | SERINC5 | rs1126176 | 79552184 | 0.3268 | 0.042458 | 6.97E-15 | A | G | 0.3688 | 0.058948 | 62.51569 |
| cg26937798 | 16 | IL4R | rs7499292 | 27326268 | 0.2699 | 0.044291 | 5.51E-10 | G | T | 0.3983 | 0.037847 | 39.25728 |
| cg26963277 | 11 | KCNQ1OT | rs463924 | 2717680 | -0.2997 | 0.045793 | 2.98E-11 | T | C | 0.3079 | 0.043332 | 45.20441 |
| cg26995224 | 12 | KDM2B | rs28758996 | 121960480 | 0.5093 | 0.043852 | 1.75E-31 | A | G | 0.4864 | 0.127678 | 146.0726 |
| cg26996569 | 12 |  | rs117748688 | 121829852 | 1.383 | 0.127365 | 9.08E-28 | C | A | 0.0195 | 0.112703 | 126.7638 |
| cg27025995 | 8 | COL22A1 | rs111698074 | 139926282 | 1.08 | 0.068985 | 1.52E-55 | A | G | 0.07979 | 0.218908 | 279.6992 |
| cg27409015 | 2 | GALNT5 | rs2197585 | 158112900 | 0.567 | 0.058206 | 1.01E-22 | C | T | 0.107 | 0.09199 | 101.1063 |
| cg27526649 | 1 |  | rs2745939 | 208142081 | 0.5894 | 0.042974 | 4.11E-43 | C | A | 0.3777 | 0.172995 | 208.7644 |
| cg27618939 | 8 | XKR9 | rs368854 | 71489682 | 0.3702 | 0.047341 | 2.64E-15 | C | A | 0.1743 | 0.060747 | 64.54605 |
