# Supplementary material for: Smoking, DNA Methylation, and Breast Cancer: A Mendelian Randomization Study
Source: Front Oncol. 2021 Sep 28;11:745918. doi: 10.3389/fonc.2021.745918 (PMC8507148; doi:10.3389/fonc.2021.745918)
Supplement: Supplementary file 3 [file Table_2.docx]

Table S2. Two-sample MR results for smoking-related CpG sites on the risk of ER+ breast cancer.

| id.outcome | outcome | exposure | method | nsnp | b | se | pval | lo_ci | up_ci | or | or_lci95 | or_uci95 |
| --- | --- | --- | --- | --- | --- | --- | --- | --- | --- | --- | --- | --- |
| ieu-a-1127 | ER+ Breast | cg2583948 | Wald ratio | 1 | -0.06494 | 0.015564 | 3.01E-05 | -0.09544 | -0.03443 | 0.937126 | 0.908971 | 0.966153 |
| ieu-a-1127 | ER+ Breast | cg0760265 | IVW | 2 | 0.069344 | 0.018759 | 0.000219 | 0.032577 | 0.106112 | 1.071805 | 1.033113 | 1.111946 |
| ieu-a-1127 | ER+ Breast | cg0420946 | Wald ratio | 1 | -0.04667 | 0.012705 | 0.00024 | -0.07157 | -0.02176 | 0.954407 | 0.930935 | 0.978471 |
| ieu-a-1127 | ER+ Breast | cg2037583 | Wald ratio | 1 | 0.090167 | 0.025762 | 0.000465 | 0.039673 | 0.14066 | 1.094356 | 1.040471 | 1.151033 |
| ieu-a-1127 | ER+ Breast | cg1227506 | Wald ratio | 1 | -0.07239 | 0.021605 | 0.000806 | -0.11474 | -0.03004 | 0.930168 | 0.891601 | 0.970402 |
| ieu-a-1127 | ER+ Breast | cg2607605 | IVW | 2 | 0.049635 | 0.015134 | 0.00104 | 0.019971 | 0.079298 | 1.050887 | 1.020172 | 1.082527 |
| ieu-a-1127 | ER+ Breast | cg1782334 | Wald ratio | 1 | 0.042161 | 0.013376 | 0.001622 | 0.015943 | 0.068378 | 1.043062 | 1.016071 | 1.07077 |
| ieu-a-1127 | ER+ Breast | cg0156570 | Wald ratio | 1 | 0.08218 | 0.026829 | 0.00219 | 0.029596 | 0.134764 | 1.085651 | 1.030038 | 1.144267 |
| ieu-a-1127 | ER+ Breast | cg1870825 | Wald ratio | 1 | -0.03831 | 0.012829 | 0.002822 | -0.06346 | -0.01317 | 0.96241 | 0.938511 | 0.986917 |
| ieu-a-1127 | ER+ Breast | cg0318838 | Wald ratio | 1 | -0.07656 | 0.026132 | 0.003394 | -0.12777 | -0.02534 | 0.926302 | 0.880053 | 0.974982 |
| ieu-a-1127 | ER+ Breast | cg2531346 | IVW | 2 | -0.04934 | 0.016923 | 0.00355 | -0.08251 | -0.01617 | 0.951856 | 0.920801 | 0.983958 |
| ieu-a-1127 | ER+ Breast | cg0702902 | IVW | 2 | -0.06144 | 0.02196 | 0.005142 | -0.10449 | -0.0184 | 0.940405 | 0.900787 | 0.981765 |
| ieu-a-1127 | ER+ Breast | cg0098165 | Wald ratio | 1 | 0.041539 | 0.015142 | 0.006084 | 0.01186 | 0.071218 | 1.042414 | 1.011931 | 1.073815 |
| ieu-a-1127 | ER+ Breast | cg2037967 | Wald ratio | 1 | -0.04132 | 0.015123 | 0.006296 | -0.07096 | -0.01167 | 0.959526 | 0.931502 | 0.988393 |
| ieu-a-1127 | ER+ Breast | cg2518990 | Wald ratio | 1 | -0.05946 | 0.021801 | 0.006386 | -0.10219 | -0.01673 | 0.942277 | 0.902862 | 0.983412 |
| ieu-a-1127 | ER+ Breast | cg0002440 | Wald ratio | 1 | 0.062034 | 0.022965 | 0.006907 | 0.017024 | 0.107044 | 1.063999 | 1.017169 | 1.112984 |
| ieu-a-1127 | ER+ Breast | cg2690832 | Wald ratio | 1 | 0.063647 | 0.023562 | 0.006907 | 0.017466 | 0.109829 | 1.065717 | 1.01762 | 1.116087 |
| ieu-a-1127 | ER+ Breast | cg1075095 | Wald ratio | 1 | 0.083192 | 0.031409 | 0.008081 | 0.02163 | 0.144754 | 1.08675 | 1.021866 | 1.155755 |
| ieu-a-1127 | ER+ Breast | cg1919741 | Wald ratio | 1 | -0.05855 | 0.022279 | 0.008584 | -0.10222 | -0.01489 | 0.943127 | 0.902829 | 0.985223 |
| ieu-a-1127 | ER+ Breast | cg2752664 | Wald ratio | 1 | -0.0358 | 0.013743 | 0.009189 | -0.06273 | -0.00886 | 0.964834 | 0.939192 | 0.991176 |
| ieu-a-1127 | ER+ Breast | cg0240547 | Wald ratio | 1 | -0.05398 | 0.021053 | 0.010344 | -0.09524 | -0.01272 | 0.94745 | 0.909151 | 0.987363 |
| ieu-a-1127 | ER+ Breast | cg0450619 | Wald ratio | 1 | -0.06552 | 0.025613 | 0.010531 | -0.11572 | -0.01531 | 0.936584 | 0.890726 | 0.984803 |
| ieu-a-1127 | ER+ Breast | cg0750679 | Wald ratio | 1 | 0.042536 | 0.016762 | 0.011157 | 0.009684 | 0.075389 | 1.043454 | 1.009731 | 1.078304 |
| ieu-a-1127 | ER+ Breast | cg2640384 | Wald ratio | 1 | 0.095636 | 0.038037 | 0.011927 | 0.021083 | 0.170188 | 1.100358 | 1.021307 | 1.185527 |
| ieu-a-1127 | ER+ Breast | cg1182751 | Wald ratio | 1 | 0.033926 | 0.01357 | 0.012419 | 0.007328 | 0.060523 | 1.034508 | 1.007355 | 1.062392 |
| ieu-a-1127 | ER+ Breast | cg1858406 | Wald ratio | 1 | -0.06751 | 0.027132 | 0.012843 | -0.12068 | -0.01433 | 0.934722 | 0.886313 | 0.985774 |
| ieu-a-1127 | ER+ Breast | cg0131404 | Wald ratio | 1 | -0.04821 | 0.019487 | 0.013373 | -0.0864 | -0.01001 | 0.952938 | 0.917227 | 0.99004 |
| ieu-a-1127 | ER+ Breast | cg1595118 | Wald ratio | 1 | -0.04103 | 0.017132 | 0.016618 | -0.07461 | -0.00745 | 0.959798 | 0.928105 | 0.992574 |
| ieu-a-1127 | ER+ Breast | cg2171750 | Wald ratio | 1 | 0.089866 | 0.037763 | 0.017325 | 0.015851 | 0.163881 | 1.094028 | 1.015977 | 1.178075 |
| ieu-a-1127 | ER+ Breast | cg1045307 | IVW | 2 | -0.05226 | 0.022243 | 0.018798 | -0.09586 | -0.00866 | 0.949081 | 0.908593 | 0.991373 |
| ieu-a-1127 | ER+ Breast | cg1422265 | Wald ratio | 1 | -0.05 | 0.021304 | 0.018928 | -0.09176 | -0.00824 | 0.951229 | 0.912327 | 0.99179 |
| ieu-a-1127 | ER+ Breast | cg1700906 | Wald ratio | 1 | -0.06602 | 0.028738 | 0.021602 | -0.12235 | -0.00969 | 0.936113 | 0.884842 | 0.990354 |
| ieu-a-1127 | ER+ Breast | cg1547457 | Wald ratio | 1 | -0.04962 | 0.021677 | 0.02207 | -0.09211 | -0.00714 | 0.95159 | 0.912007 | 0.99289 |
| ieu-a-1127 | ER+ Breast | cg1562688 | Wald ratio | 1 | -0.06369 | 0.028279 | 0.024311 | -0.11911 | -0.00826 | 0.938297 | 0.887706 | 0.991772 |
| ieu-a-1127 | ER+ Breast | cg0102098 | Wald ratio | 1 | -0.04167 | 0.018715 | 0.02596 | -0.07836 | -0.00499 | 0.959182 | 0.924635 | 0.995019 |
| ieu-a-1127 | ER+ Breast | cg1980448 | Wald ratio | 1 | -0.05929 | 0.026658 | 0.026148 | -0.11154 | -0.00704 | 0.942436 | 0.894458 | 0.992987 |
| ieu-a-1127 | ER+ Breast | cg1110535 | Wald ratio | 1 | -0.02591 | 0.011778 | 0.027807 | -0.049 | -0.00283 | 0.974422 | 0.952185 | 0.997177 |

| ieu-a-1127 | ER+ Breast | cg1439158 | Wald ratio | 1 | -0.02908 | 0.013315 | 0.028947 | -0.05518 | -0.00299 | 0.971337 | 0.946316 | 0.997019 |
| --- | --- | --- | --- | --- | --- | --- | --- | --- | --- | --- | --- | --- |
| ieu-a-1127 | ER+ Breast | cg0337339 | Inverse var | 2 | -0.0424 | 0.020191 | 0.035726 | -0.08198 | -0.00283 | 0.958484 | 0.921293 | 0.997177 |
| ieu-a-1127 | ER+ Breast | cg1380944 | Wald ratio | 1 | 0.032984 | 0.015849 | 0.037426 | 0.001919 | 0.064048 | 1.033533 | 1.001921 | 1.066144 |
| ieu-a-1127 | ER+ Breast | cg1693695 | Wald ratio | 1 | -0.05109 | 0.025109 | 0.041863 | -0.10031 | -0.00188 | 0.950189 | 0.904557 | 0.998121 |
| ieu-a-1127 | ER+ Breast | cg0399187 | Wald ratio | 1 | 0.041387 | 0.020443 | 0.042918 | 0.001319 | 0.081455 | 1.042255 | 1.00132 | 1.084864 |
| ieu-a-1127 | ER+ Breast | cg0156125 | Wald ratio | 1 | -0.06575 | 0.032663 | 0.044134 | -0.12977 | -0.00173 | 0.936369 | 0.878301 | 0.998276 |
| ieu-a-1127 | ER+ Breast | cg1987209 | Wald ratio | 1 | 0.097004 | 0.048502 | 0.0455 | 0.00194 | 0.192068 | 1.101865 | 1.001942 | 1.211753 |
| ieu-a-1127 | ER+ Breast | cg0193780 | Wald ratio | 1 | 0.089567 | 0.044784 | 0.0455 | 0.001791 | 0.177343 | 1.093701 | 1.001793 | 1.19404 |
| ieu-a-1127 | ER+ Breast | cg0598778 | Wald ratio | 1 | -0.04945 | 0.024887 | 0.046921 | -0.09823 | -0.00067 | 0.951752 | 0.906441 | 0.999328 |
| ieu-a-1127 | ER+ Breast | cg0371002 | Wald ratio | 1 | -0.03446 | 0.017418 | 0.047899 | -0.0686 | -0.00032 | 0.96613 | 0.933705 | 0.999682 |
| ieu-a-1127 | ER+ Breast | cg1433029 | Wald ratio | 1 | 0.069054 | 0.035379 | 0.050961 | -0.00029 | 0.138397 | 1.071494 | 0.99971 | 1.148432 |
| ieu-a-1127 | ER+ Breast | cg0433753 | Wald ratio | 1 | -0.06746 | 0.034878 | 0.053087 | -0.13582 | 0.000899 | 0.934763 | 0.872996 | 1.0009 |
| ieu-a-1127 | ER+ Breast | cg0638266 | Wald ratio | 1 | -0.04243 | 0.0222 | 0.05599 | -0.08594 | 0.001085 | 0.95846 | 0.917649 | 1.001086 |
| ieu-a-1127 | ER+ Breast | cg0782742 | Wald ratio | 1 | -0.05489 | 0.028991 | 0.058314 | -0.11171 | 0.001933 | 0.946589 | 0.894301 | 1.001935 |
| ieu-a-1127 | ER+ Breast | cg0727703 | Inverse var | 3 | 0.030471 | 0.016149 | 0.059179 | -0.00118 | 0.062122 | 1.03094 | 0.99882 | 1.064093 |
| ieu-a-1127 | ER+ Breast | cg0218644 | Wald ratio | 1 | -0.04363 | 0.02329 | 0.061011 | -0.08928 | 0.002017 | 0.957306 | 0.914589 | 1.002019 |
| ieu-a-1127 | ER+ Breast | cg0847947 | Wald ratio | 1 | 0.033824 | 0.018112 | 0.061836 | -0.00168 | 0.069325 | 1.034403 | 0.998325 | 1.071784 |
| ieu-a-1127 | ER+ Breast | cg0632159 | Wald ratio | 1 | -0.03569 | 0.019235 | 0.063536 | -0.07339 | 0.002012 | 0.96494 | 0.929238 | 1.002014 |
| ieu-a-1127 | ER+ Breast | cg0619304 | Wald ratio | 1 | 0.017953 | 0.009874 | 0.069036 | -0.0014 | 0.037307 | 1.018115 | 0.998601 | 1.038012 |
| ieu-a-1127 | ER+ Breast | cg1419498 | Wald ratio | 1 | 0.019666 | 0.010816 | 0.069036 | -0.00153 | 0.040865 | 1.01986 | 0.998467 | 1.041712 |
| ieu-a-1127 | ER+ Breast | cg1755189 | Inverse var | 2 | -0.03221 | 0.017747 | 0.069551 | -0.06699 | 0.002576 | 0.968306 | 0.935204 | 1.00258 |
| ieu-a-1127 | ER+ Breast | cg1995691 | Wald ratio | 1 | 0.048233 | 0.027035 | 0.074409 | -0.00476 | 0.101223 | 1.049416 | 0.995256 | 1.106523 |
| ieu-a-1127 | ER+ Breast | cg1344360 | Wald ratio | 1 | 0.052742 | 0.029667 | 0.07544 | -0.00541 | 0.11089 | 1.054158 | 0.994609 | 1.117272 |
| ieu-a-1127 | ER+ Breast | cg0993538 | Wald ratio | 1 | -0.06741 | 0.038673 | 0.081324 | -0.14321 | 0.00839 | 0.934812 | 0.866573 | 1.008425 |
| ieu-a-1127 | ER+ Breast | cg1814673 | Wald ratio | 1 | -0.05422 | 0.031105 | 0.081324 | -0.11518 | 0.006748 | 0.947226 | 0.891203 | 1.006771 |
| ieu-a-1127 | ER+ Breast | cg2574153 | Wald ratio | 1 | -0.03231 | 0.018669 | 0.083493 | -0.0689 | 0.00428 | 0.968204 | 0.933416 | 1.004289 |
| ieu-a-1127 | ER+ Breast | cg0386421 | Wald ratio | 1 | -0.03387 | 0.019608 | 0.084119 | -0.0723 | 0.004563 | 0.966699 | 0.930252 | 1.004574 |
| ieu-a-1127 | ER+ Breast | cg2670350 | Wald ratio | 1 | -0.03177 | 0.018473 | 0.085432 | -0.06798 | 0.004433 | 0.968726 | 0.934279 | 1.004443 |
| ieu-a-1127 | ER+ Breast | cg1882444 | Wald ratio | 1 | -0.02069 | 0.01216 | 0.088887 | -0.04452 | 0.003146 | 0.979524 | 0.956454 | 1.003151 |
| ieu-a-1127 | ER+ Breast | cg2528439 | Wald ratio | 1 | -0.04873 | 0.028663 | 0.089131 | -0.10491 | 0.007452 | 0.952441 | 0.900409 | 1.00748 |
| ieu-a-1127 | ER+ Breast | cg1069644 | Wald ratio | 1 | -0.04007 | 0.023755 | 0.091652 | -0.08663 | 0.006491 | 0.960723 | 0.917018 | 1.006512 |
| ieu-a-1127 | ER+ Breast | cg0165191 | Wald ratio | 1 | -0.04373 | 0.026055 | 0.093236 | -0.0948 | 0.007333 | 0.957208 | 0.909553 | 1.007359 |
| ieu-a-1127 | ER+ Breast | cg0606501 | Wald ratio | 1 | -0.02874 | 0.017126 | 0.093284 | -0.06231 | 0.004824 | 0.971667 | 0.939593 | 1.004836 |
| ieu-a-1127 | ER+ Breast | cg0397090 | Wald ratio | 1 | 0.029306 | 0.017499 | 0.093993 | -0.00499 | 0.063605 | 1.02974 | 0.99502 | 1.065672 |
| ieu-a-1127 | ER+ Breast | cg0757223 | Wald ratio | 1 | 0.044424 | 0.026529 | 0.094017 | -0.00757 | 0.096421 | 1.045426 | 0.992457 | 1.101222 |
| ieu-a-1127 | ER+ Breast | cg0089360 | Wald ratio | 1 | 0.023359 | 0.01398 | 0.094744 | -0.00404 | 0.050759 | 1.023634 | 0.995966 | 1.052069 |
| ieu-a-1127 | ER+ Breast | cg2120140 | Wald ratio | 1 | 0.062185 | 0.037395 | 0.096328 | -0.01111 | 0.135479 | 1.064159 | 0.988952 | 1.145085 |
| ieu-a-1127 | ER+ Breast | cg0080648 | Wald ratio | 1 | 0.017967 | 0.010824 | 0.096914 | -0.00325 | 0.039182 | 1.01813 | 0.996758 | 1.039959 |

| ieu-a-1127 | ER+ Breast | cg1303925 | Wald ratio | 1 | 0.039289 | 0.023761 | 0.09822 | -0.00728 | 0.08586 | 1.040071 | 0.992745 | 1.089653 |
| --- | --- | --- | --- | --- | --- | --- | --- | --- | --- | --- | --- | --- |
| ieu-a-1127 | ER+ Breast | cg2658564 | Wald ratio | 1 | -0.04528 | 0.027684 | 0.101941 | -0.09954 | 0.008983 | 0.955732 | 0.905254 | 1.009024 |
| ieu-a-1127 | ER+ Breast | cg1018009 | Inverse var | 2 | -0.02023 | 0.012372 | 0.102098 | -0.04447 | 0.004024 | 0.979978 | 0.9565 | 1.004032 |
| ieu-a-1127 | ER+ Breast | cg0149890 | Wald ratio | 1 | 0.028464 | 0.017643 | 0.106672 | -0.00612 | 0.063044 | 1.028873 | 0.993902 | 1.065074 |
| ieu-a-1127 | ER+ Breast | cg2322248 | Wald ratio | 1 | 0.061319 | 0.038219 | 0.108627 | -0.01359 | 0.136228 | 1.063238 | 0.986501 | 1.145944 |
| ieu-a-1127 | ER+ Breast | cg1345216 | Wald ratio | 1 | 0.03947 | 0.024629 | 0.109031 | -0.0088 | 0.087742 | 1.040259 | 0.991235 | 1.091707 |
| ieu-a-1127 | ER+ Breast | cg0521446 | Wald ratio | 1 | -0.0329 | 0.020658 | 0.111252 | -0.07339 | 0.00759 | 0.967636 | 0.929239 | 1.007619 |
| ieu-a-1127 | ER+ Breast | cg1484151 | Wald ratio | 1 | -0.04533 | 0.028918 | 0.116982 | -0.10201 | 0.011348 | 0.955682 | 0.903022 | 1.011413 |
| ieu-a-1127 | ER+ Breast | cg0007346 | Wald ratio | 1 | 0.045726 | 0.029487 | 0.120968 | -0.01207 | 0.103521 | 1.046788 | 0.988004 | 1.109069 |
| ieu-a-1127 | ER+ Breast | cg0930726 | Wald ratio | 1 | -0.02763 | 0.017838 | 0.121412 | -0.06259 | 0.007334 | 0.972749 | 0.939327 | 1.007361 |
| ieu-a-1127 | ER+ Breast | cg0696891 | Wald ratio | 1 | -0.06569 | 0.042469 | 0.121921 | -0.14893 | 0.01755 | 0.936422 | 0.861632 | 1.017705 |
| ieu-a-1127 | ER+ Breast | cg2264249 | Wald ratio | 1 | -0.05 | 0.032645 | 0.12561 | -0.11398 | 0.013983 | 0.951229 | 0.892273 | 1.014082 |
| ieu-a-1127 | ER+ Breast | cg0566153 | Wald ratio | 1 | -0.06498 | 0.042501 | 0.126303 | -0.14828 | 0.018324 | 0.937089 | 0.86219 | 1.018493 |
| ieu-a-1127 | ER+ Breast | cg1190277 | Wald ratio | 1 | -0.03666 | 0.024078 | 0.127878 | -0.08385 | 0.010534 | 0.964004 | 0.919567 | 1.010589 |
| ieu-a-1127 | ER+ Breast | cg2085388 | Wald ratio | 1 | -0.03473 | 0.022823 | 0.128074 | -0.07946 | 0.010002 | 0.965866 | 0.923612 | 1.010052 |
| ieu-a-1127 | ER+ Breast | cg1712770 | Inverse var | 2 | -0.01643 | 0.010833 | 0.129437 | -0.03766 | 0.004806 | 0.983708 | 0.963042 | 1.004818 |
| ieu-a-1127 | ER+ Breast | cg1969649 | Wald ratio | 1 | -0.03002 | 0.019926 | 0.131896 | -0.06908 | 0.009033 | 0.970425 | 0.933256 | 1.009074 |
| ieu-a-1127 | ER+ Breast | cg2256381 | Wald ratio | 1 | -0.03196 | 0.02121 | 0.131896 | -0.07353 | 0.009615 | 0.968548 | 0.929109 | 1.009662 |
| ieu-a-1127 | ER+ Breast | cg1753347 | Wald ratio | 1 | 0.042611 | 0.028408 | 0.133614 | -0.01307 | 0.09829 | 1.043532 | 0.987018 | 1.103283 |
| ieu-a-1127 | ER+ Breast | cg1166001 | Wald ratio | 1 | 0.041418 | 0.027801 | 0.136278 | -0.01307 | 0.095909 | 1.042288 | 0.987013 | 1.100659 |
| ieu-a-1127 | ER+ Breast | cg1146180 | Wald ratio | 1 | -0.03335 | 0.022612 | 0.140213 | -0.07767 | 0.010967 | 0.967198 | 0.925269 | 1.011027 |
| ieu-a-1127 | ER+ Breast | cg2453951 | Wald ratio | 1 | 0.021337 | 0.014812 | 0.149733 | -0.0077 | 0.050369 | 1.021566 | 0.992334 | 1.051659 |
| ieu-a-1127 | ER+ Breast | cg0588662 | Wald ratio | 1 | -0.03558 | 0.024924 | 0.153441 | -0.08443 | 0.013272 | 0.965047 | 0.919037 | 1.013361 |
| ieu-a-1127 | ER+ Breast | cg1993506 | Wald ratio | 1 | 0.039485 | 0.028118 | 0.160243 | -0.01563 | 0.094598 | 1.040275 | 0.984495 | 1.099217 |
| ieu-a-1127 | ER+ Breast | cg0287165 | Wald ratio | 1 | -0.01898 | 0.01354 | 0.160897 | -0.04552 | 0.007554 | 0.981195 | 0.955499 | 1.007583 |
| ieu-a-1127 | ER+ Breast | cg0118534 | Wald ratio | 1 | -0.03074 | 0.022061 | 0.163433 | -0.07398 | 0.012495 | 0.969724 | 0.928688 | 1.012573 |
| ieu-a-1127 | ER+ Breast | cg1280668 | Wald ratio | 1 | 0.030623 | 0.022042 | 0.164728 | -0.01258 | 0.073825 | 1.031097 | 0.987501 | 1.076618 |
| ieu-a-1127 | ER+ Breast | cg1902269 | Wald ratio | 1 | -0.02712 | 0.019556 | 0.165564 | -0.06545 | 0.011214 | 0.973247 | 0.936648 | 1.011277 |
| ieu-a-1127 | ER+ Breast | cg1508907 | Wald ratio | 1 | -0.02004 | 0.014456 | 0.165564 | -0.04838 | 0.008289 | 0.980155 | 0.952773 | 1.008324 |
| ieu-a-1127 | ER+ Breast | cg0838423 | Wald ratio | 1 | 0.020197 | 0.01458 | 0.165996 | -0.00838 | 0.048774 | 1.020402 | 0.991654 | 1.049983 |
| ieu-a-1127 | ER+ Breast | cg1025576 | Wald ratio | 1 | -0.03596 | 0.026122 | 0.168676 | -0.08715 | 0.015243 | 0.964683 | 0.916536 | 1.01536 |
| ieu-a-1127 | ER+ Breast | cg1903055 | Inverse var | 2 | 0.016465 | 0.011966 | 0.168822 | -0.00699 | 0.039918 | 1.016601 | 0.993036 | 1.040725 |
| ieu-a-1127 | ER+ Breast | cg1975844 | Wald ratio | 1 | 0.019169 | 0.014022 | 0.171598 | -0.00831 | 0.046652 | 1.019354 | 0.991721 | 1.047758 |
| ieu-a-1127 | ER+ Breast | cg2580990 | Wald ratio | 1 | 0.021648 | 0.016187 | 0.181084 | -0.01008 | 0.053374 | 1.021884 | 0.989973 | 1.054824 |
| ieu-a-1127 | ER+ Breast | cg2053389 | Wald ratio | 1 | -0.03523 | 0.026419 | 0.182422 | -0.08701 | 0.016556 | 0.965388 | 0.916672 | 1.016694 |
| ieu-a-1127 | ER+ Breast | cg2403312 | Wald ratio | 1 | 0.014493 | 0.010904 | 0.18381 | -0.00688 | 0.035865 | 1.014598 | 0.993144 | 1.036516 |
| ieu-a-1127 | ER+ Breast | cg1250409 | Inverse var | 2 | 0.029109 | 0.022594 | 0.197612 | -0.01517 | 0.073393 | 1.029537 | 0.98494 | 1.076154 |
| ieu-a-1127 | ER+ Breast | cg1758609 | Wald ratio | 1 | -0.0227 | 0.017633 | 0.19792 | -0.05726 | 0.011858 | 0.977554 | 0.944346 | 1.011929 |

| ieu-a-1127 | ER+ Breast | cg0439699 | Wald ratio | 1 | 0.020643 | 0.016219 | 0.203115 | -0.01115 | 0.052433 | 1.020857 | 0.988915 | 1.053832 |
| --- | --- | --- | --- | --- | --- | --- | --- | --- | --- | --- | --- | --- |
| ieu-a-1127 | ER+ Breast | cg1894653 | Wald ratio | 1 | -0.04197 | 0.033057 | 0.204167 | -0.10677 | 0.022817 | 0.958895 | 0.898737 | 1.023079 |
| ieu-a-1127 | ER+ Breast | cg0227962 | Wald ratio | 1 | -0.02821 | 0.022215 | 0.204167 | -0.07175 | 0.015334 | 0.972186 | 0.930763 | 1.015452 |
| ieu-a-1127 | ER+ Breast | cg0628347 | Wald ratio | 1 | -0.03933 | 0.031261 | 0.208368 | -0.1006 | 0.021943 | 0.961436 | 0.904296 | 1.022185 |
| ieu-a-1127 | ER+ Breast | cg2605595 | Wald ratio | 1 | -0.02769 | 0.022046 | 0.209183 | -0.07089 | 0.015524 | 0.972695 | 0.93156 | 1.015645 |
| ieu-a-1127 | ER+ Breast | cg1412089 | Wald ratio | 1 | -0.03481 | 0.027766 | 0.209898 | -0.08924 | 0.019607 | 0.965785 | 0.91463 | 1.0198 |
| ieu-a-1127 | ER+ Breast | cg1132350 | Wald ratio | 1 | -0.02701 | 0.02161 | 0.2113 | -0.06937 | 0.015343 | 0.973348 | 0.932982 | 1.015462 |
| ieu-a-1127 | ER+ Breast | cg1059096 | Wald ratio | 1 | 0.026096 | 0.020926 | 0.212376 | -0.01492 | 0.067111 | 1.02644 | 0.985192 | 1.069414 |
| ieu-a-1127 | ER+ Breast | cg0020560 | Wald ratio | 1 | -0.01512 | 0.012157 | 0.213651 | -0.03895 | 0.008709 | 0.984995 | 0.961802 | 1.008748 |
| ieu-a-1127 | ER+ Breast | cg1803341 | Wald ratio | 1 | -0.03985 | 0.032127 | 0.214788 | -0.10282 | 0.023115 | 0.96093 | 0.902287 | 1.023384 |
| ieu-a-1127 | ER+ Breast | cg0900648 | Wald ratio | 1 | -0.03135 | 0.025309 | 0.21548 | -0.08095 | 0.018257 | 0.969137 | 0.922235 | 1.018425 |
| ieu-a-1127 | ER+ Breast | cg1505980 | Wald ratio | 1 | 0.016521 | 0.013435 | 0.218798 | -0.00981 | 0.042854 | 1.016659 | 0.990237 | 1.043786 |
| ieu-a-1127 | ER+ Breast | cg1569348 | Wald ratio | 1 | -0.01241 | 0.010111 | 0.219551 | -0.03223 | 0.007404 | 0.987663 | 0.968282 | 1.007432 |
| ieu-a-1127 | ER+ Breast | cg0906907 | Wald ratio | 1 | 0.01418 | 0.011571 | 0.220391 | -0.0085 | 0.03686 | 1.014281 | 0.991537 | 1.037548 |
| ieu-a-1127 | ER+ Breast | cg1603798 | Wald ratio | 1 | 0.029144 | 0.023983 | 0.224294 | -0.01786 | 0.076151 | 1.029573 | 0.982296 | 1.079125 |
| ieu-a-1127 | ER+ Breast | cg1590303 | Wald ratio | 1 | -0.03252 | 0.026839 | 0.225602 | -0.08513 | 0.020082 | 0.968 | 0.918395 | 1.020285 |
| ieu-a-1127 | ER+ Breast | cg2492457 | Wald ratio | 1 | -0.0283 | 0.023412 | 0.22678 | -0.07419 | 0.01759 | 0.972099 | 0.928499 | 1.017745 |
| ieu-a-1127 | ER+ Breast | cg2032219 | Wald ratio | 1 | 0.031776 | 0.026322 | 0.227352 | -0.01982 | 0.083367 | 1.032286 | 0.98038 | 1.086941 |
| ieu-a-1127 | ER+ Breast | cg1850367 | Wald ratio | 1 | -0.03306 | 0.027501 | 0.229314 | -0.08696 | 0.020843 | 0.96748 | 0.916711 | 1.021061 |
| ieu-a-1127 | ER+ Breast | cg1455051 | Wald ratio | 1 | -0.03025 | 0.025167 | 0.229314 | -0.07958 | 0.019074 | 0.970199 | 0.923502 | 1.019257 |
| ieu-a-1127 | ER+ Breast | cg1678645 | Inverse var | 2 | -0.01782 | 0.014887 | 0.231184 | -0.047 | 0.011354 | 0.982333 | 0.954083 | 1.011419 |
| ieu-a-1127 | ER+ Breast | cg1160129 | Wald ratio | 1 | -0.02788 | 0.023434 | 0.234182 | -0.07381 | 0.018053 | 0.972506 | 0.928848 | 1.018216 |
| ieu-a-1127 | ER+ Breast | cg0418004 | Wald ratio | 1 | 0.027792 | 0.023457 | 0.236103 | -0.01818 | 0.073768 | 1.028182 | 0.98198 | 1.076558 |
| ieu-a-1127 | ER+ Breast | cg1627467 | Inverse var | 2 | 0.019615 | 0.016565 | 0.236376 | -0.01285 | 0.052082 | 1.019808 | 0.987229 | 1.053463 |
| ieu-a-1127 | ER+ Breast | cg2258656 | Wald ratio | 1 | -0.01783 | 0.015116 | 0.238204 | -0.04746 | 0.011798 | 0.982329 | 0.953651 | 1.011868 |
| ieu-a-1127 | ER+ Breast | cg1314237 | Wald ratio | 1 | -0.03682 | 0.031456 | 0.241783 | -0.09847 | 0.024833 | 0.963849 | 0.90622 | 1.025144 |
| ieu-a-1127 | ER+ Breast | cg1843289 | Wald ratio | 1 | -0.01656 | 0.014406 | 0.250381 | -0.0448 | 0.011677 | 0.983577 | 0.956193 | 1.011746 |
| ieu-a-1127 | ER+ Breast | cg1709810 | Wald ratio | 1 | 0.024674 | 0.021554 | 0.252318 | -0.01757 | 0.06692 | 1.024981 | 0.982581 | 1.06921 |
| ieu-a-1127 | ER+ Breast | cg1424289 | Wald ratio | 1 | 0.033852 | 0.029582 | 0.252487 | -0.02413 | 0.091833 | 1.034431 | 0.976159 | 1.096182 |
| ieu-a-1127 | ER+ Breast | cg2657477 | Wald ratio | 1 | -0.02245 | 0.019776 | 0.256319 | -0.06121 | 0.016312 | 0.977802 | 0.940628 | 1.016446 |
| ieu-a-1127 | ER+ Breast | cg1497541 | Wald ratio | 1 | 0.023911 | 0.021074 | 0.256537 | -0.01739 | 0.065216 | 1.024199 | 0.982756 | 1.067389 |
| ieu-a-1127 | ER+ Breast | cg1861709 | Wald ratio | 1 | 0.018649 | 0.016465 | 0.25736 | -0.01362 | 0.050921 | 1.018824 | 0.98647 | 1.052239 |
| ieu-a-1127 | ER+ Breast | cg0652268 | Wald ratio | 1 | -0.02065 | 0.018229 | 0.25736 | -0.05638 | 0.015082 | 0.979564 | 0.945183 | 1.015196 |
| ieu-a-1127 | ER+ Breast | cg2394061 | Wald ratio | 1 | 0.015997 | 0.014137 | 0.257811 | -0.01171 | 0.043705 | 1.016126 | 0.988357 | 1.044675 |
| ieu-a-1127 | ER+ Breast | cg2625350 | Wald ratio | 1 | 0.028105 | 0.025083 | 0.262508 | -0.02106 | 0.077268 | 1.028504 | 0.979162 | 1.080332 |
| ieu-a-1127 | ER+ Breast | cg1001251 | Wald ratio | 1 | 0.024833 | 0.022321 | 0.265923 | -0.01892 | 0.068583 | 1.025143 | 0.98126 | 1.070989 |
| ieu-a-1127 | ER+ Breast | cg0267275 | Wald ratio | 1 | 0.035805 | 0.032188 | 0.265984 | -0.02728 | 0.098893 | 1.036453 | 0.973085 | 1.103949 |
| ieu-a-1127 | ER+ Breast | cg0414453 | Wald ratio | 1 | 0.020609 | 0.018548 | 0.266521 | -0.01575 | 0.056964 | 1.020823 | 0.984378 | 1.058617 |

| ieu-a-1127 | ER+ Breast | cg1401687 | Wald ratio | 1 | 0.022143 | 0.019929 | 0.266521 | -0.01692 | 0.061205 | 1.02239 | 0.983225 | 1.063116 |
| --- | --- | --- | --- | --- | --- | --- | --- | --- | --- | --- | --- | --- |
| ieu-a-1127 | ER+ Breast | cg0993847 | Wald ratio | 1 | 0.024148 | 0.022029 | 0.272983 | -0.01903 | 0.067325 | 1.024442 | 0.981152 | 1.069643 |
| ieu-a-1127 | ER+ Breast | cg1557814 | Wald ratio | 1 | 0.032282 | 0.029714 | 0.277293 | -0.02596 | 0.090521 | 1.032808 | 0.974377 | 1.094744 |
| ieu-a-1127 | ER+ Breast | cg0378575 | Wald ratio | 1 | 0.027598 | 0.025438 | 0.277963 | -0.02226 | 0.077456 | 1.027982 | 0.977985 | 1.080535 |
| ieu-a-1127 | ER+ Breast | cg0017885 | Wald ratio | 1 | 0.03012 | 0.027763 | 0.277963 | -0.0243 | 0.084536 | 1.030579 | 0.975997 | 1.088212 |
| ieu-a-1127 | ER+ Breast | cg0695162 | Wald ratio | 1 | 0.027519 | 0.025365 | 0.277963 | -0.0222 | 0.077234 | 1.027901 | 0.978048 | 1.080295 |
| ieu-a-1127 | ER+ Breast | cg0323477 | Wald ratio | 1 | 0.019206 | 0.017714 | 0.278271 | -0.01551 | 0.053925 | 1.019391 | 0.984606 | 1.055405 |
| ieu-a-1127 | ER+ Breast | cg1084327 | Wald ratio | 1 | 0.015761 | 0.014674 | 0.282789 | -0.013 | 0.044522 | 1.015886 | 0.987084 | 1.045528 |
| ieu-a-1127 | ER+ Breast | cg0809758 | Wald ratio | 1 | 0.014574 | 0.013773 | 0.289992 | -0.01242 | 0.04157 | 1.014681 | 0.987655 | 1.042446 |
| ieu-a-1127 | ER+ Breast | cg0057940 | Wald ratio | 1 | 0.015225 | 0.014464 | 0.29251 | -0.01312 | 0.043575 | 1.015342 | 0.986962 | 1.044538 |
| ieu-a-1127 | ER+ Breast | cg0401963 | Wald ratio | 1 | -0.02142 | 0.02036 | 0.292823 | -0.06132 | 0.018488 | 0.97881 | 0.94052 | 1.01866 |
| ieu-a-1127 | ER+ Breast | cg0262907 | Wald ratio | 1 | -0.02615 | 0.025092 | 0.297363 | -0.07533 | 0.023032 | 0.97419 | 0.927437 | 1.0233 |
| ieu-a-1127 | ER+ Breast | cg0401713 | Inverse var | 2 | -0.01654 | 0.015991 | 0.300855 | -0.04789 | 0.014798 | 0.983592 | 0.953243 | 1.014908 |
| ieu-a-1127 | ER+ Breast | cg0128934 | Wald ratio | 1 | -0.01592 | 0.015515 | 0.304904 | -0.04633 | 0.014491 | 0.984208 | 0.95473 | 1.014597 |
| ieu-a-1127 | ER+ Breast | cg2012461 | Wald ratio | 1 | 0.027019 | 0.026405 | 0.306187 | -0.02473 | 0.078772 | 1.027387 | 0.975569 | 1.081957 |
| ieu-a-1127 | ER+ Breast | cg0049821 | Inverse var | 2 | -0.01871 | 0.018449 | 0.310402 | -0.05487 | 0.017446 | 0.98146 | 0.946604 | 1.017599 |
| ieu-a-1127 | ER+ Breast | cg0083519 | Wald ratio | 1 | -0.02214 | 0.021881 | 0.311584 | -0.06503 | 0.020745 | 0.978102 | 0.937042 | 1.020962 |
| ieu-a-1127 | ER+ Breast | cg2424979 | Wald ratio | 1 | 0.010526 | 0.010606 | 0.320963 | -0.01026 | 0.031314 | 1.010582 | 0.989791 | 1.03181 |
| ieu-a-1127 | ER+ Breast | cg0909983 | Wald ratio | 1 | 0.024067 | 0.024684 | 0.32956 | -0.02431 | 0.072447 | 1.024359 | 0.97598 | 1.075136 |
| ieu-a-1127 | ER+ Breast | cg0750286 | Wald ratio | 1 | 0.023711 | 0.024476 | 0.33267 | -0.02426 | 0.071684 | 1.023995 | 0.97603 | 1.074316 |
| ieu-a-1127 | ER+ Breast | cg0164360 | Wald ratio | 1 | 0.027046 | 0.0281 | 0.335799 | -0.02803 | 0.082122 | 1.027415 | 0.97236 | 1.085588 |
| ieu-a-1127 | ER+ Breast | cg2613273 | Wald ratio | 1 | 0.032352 | 0.034208 | 0.344283 | -0.0347 | 0.099401 | 1.032881 | 0.965899 | 1.104509 |
| ieu-a-1127 | ER+ Breast | cg1445901 | Wald ratio | 1 | -0.03095 | 0.033084 | 0.349539 | -0.0958 | 0.033895 | 0.969524 | 0.90865 | 1.034476 |
| ieu-a-1127 | ER+ Breast | cg1305789 | Wald ratio | 1 | 0.013642 | 0.014594 | 0.3499 | -0.01496 | 0.042246 | 1.013736 | 0.985149 | 1.043151 |
| ieu-a-1127 | ER+ Breast | cg1152791 | Wald ratio | 1 | -0.02454 | 0.026731 | 0.358602 | -0.07693 | 0.027853 | 0.975759 | 0.925952 | 1.028244 |
| ieu-a-1127 | ER+ Breast | cg0720704 | Wald ratio | 1 | -0.00968 | 0.010642 | 0.36287 | -0.03054 | 0.011175 | 0.990363 | 0.969919 | 1.011238 |
| ieu-a-1127 | ER+ Breast | cg0299824 | Wald ratio | 1 | -0.00893 | 0.009823 | 0.363302 | -0.02818 | 0.010323 | 0.99111 | 0.972211 | 1.010376 |
| ieu-a-1127 | ER+ Breast | cg0970170 | Wald ratio | 1 | -0.01354 | 0.01511 | 0.370197 | -0.04316 | 0.016075 | 0.986551 | 0.957763 | 1.016205 |
| ieu-a-1127 | ER+ Breast | cg0278251 | Wald ratio | 1 | 0.031065 | 0.034763 | 0.371527 | -0.03707 | 0.099201 | 1.031553 | 0.963608 | 1.104288 |
| ieu-a-1127 | ER+ Breast | cg1975717 | Wald ratio | 1 | 0.01278 | 0.01455 | 0.379738 | -0.01574 | 0.041298 | 1.012862 | 0.984386 | 1.042162 |
| ieu-a-1127 | ER+ Breast | cg1881069 | Wald ratio | 1 | -0.01146 | 0.013077 | 0.380735 | -0.03709 | 0.014169 | 0.988603 | 0.963586 | 1.014269 |
| ieu-a-1127 | ER+ Breast | cg1121223 | Wald ratio | 1 | 0.011547 | 0.013279 | 0.384538 | -0.01448 | 0.037575 | 1.011614 | 0.985624 | 1.03829 |
| ieu-a-1127 | ER+ Breast | cg2192976 | Wald ratio | 1 | 0.02026 | 0.02333 | 0.385164 | -0.02547 | 0.065986 | 1.020467 | 0.974855 | 1.068212 |
| ieu-a-1127 | ER+ Breast | cg1836903 | Inverse var | 2 | 0.016578 | 0.019248 | 0.389079 | -0.02115 | 0.054304 | 1.016716 | 0.979074 | 1.055805 |
| ieu-a-1127 | ER+ Breast | cg1815103 | Wald ratio | 1 | 0.019856 | 0.023083 | 0.389671 | -0.02539 | 0.065098 | 1.020054 | 0.974934 | 1.067264 |
| ieu-a-1127 | ER+ Breast | cg0015394 | Wald ratio | 1 | -0.01846 | 0.021494 | 0.390355 | -0.06059 | 0.023665 | 0.981707 | 0.941209 | 1.023947 |
| ieu-a-1127 | ER+ Breast | cg2215805 | Wald ratio | 1 | -0.01485 | 0.017378 | 0.392803 | -0.04891 | 0.01921 | 0.98526 | 0.952267 | 1.019396 |
| ieu-a-1127 | ER+ Breast | cg2377136 | Wald ratio | 1 | 0.021926 | 0.025795 | 0.395325 | -0.02863 | 0.072485 | 1.022168 | 0.971773 | 1.075177 |

| ieu-a-1127 | ER+ Breast | cg2130748 | Wald ratio | 1 | -0.02145 | 0.025272 | 0.395972 | -0.07098 | 0.028081 | 0.978777 | 0.931476 | 1.028479 |
| --- | --- | --- | --- | --- | --- | --- | --- | --- | --- | --- | --- | --- |
| ieu-a-1127 | ER+ Breast | cg1140565 | Wald ratio | 1 | 0.020281 | 0.024007 | 0.398207 | -0.02677 | 0.067334 | 1.020489 | 0.973584 | 1.069653 |
| ieu-a-1127 | ER+ Breast | cg0537935 | Wald ratio | 1 | -0.02242 | 0.02733 | 0.411924 | -0.07599 | 0.031142 | 0.977825 | 0.926824 | 1.031632 |
| ieu-a-1127 | ER+ Breast | cg0246241 | Wald ratio | 1 | 0.010496 | 0.012974 | 0.418522 | -0.01493 | 0.035924 | 1.010551 | 0.985178 | 1.036577 |
| ieu-a-1127 | ER+ Breast | cg0270450 | Wald ratio | 1 | 0.011465 | 0.014172 | 0.418522 | -0.01631 | 0.039242 | 1.011531 | 0.98382 | 1.040022 |
| ieu-a-1127 | ER+ Breast | cg0126876 | Wald ratio | 1 | -0.01936 | 0.024254 | 0.424826 | -0.06689 | 0.028181 | 0.98083 | 0.935295 | 1.028582 |
| ieu-a-1127 | ER+ Breast | cg1282872 | Wald ratio | 1 | 0.016411 | 0.020706 | 0.428015 | -0.02417 | 0.056994 | 1.016546 | 0.976118 | 1.058649 |
| ieu-a-1127 | ER+ Breast | cg1682203 | Wald ratio | 1 | -0.0258 | 0.032648 | 0.429456 | -0.08979 | 0.038194 | 0.974534 | 0.914126 | 1.038933 |
| ieu-a-1127 | ER+ Breast | cg2740901 | Wald ratio | 1 | 0.018519 | 0.023457 | 0.429835 | -0.02746 | 0.064494 | 1.018691 | 0.972917 | 1.066619 |
| ieu-a-1127 | ER+ Breast | cg2693779 | Wald ratio | 1 | -0.02223 | 0.028159 | 0.429835 | -0.07742 | 0.03296 | 0.978015 | 0.9255 | 1.03351 |
| ieu-a-1127 | ER+ Breast | cg2388424 | Wald ratio | 1 | 0.012256 | 0.015568 | 0.431145 | -0.01826 | 0.042769 | 1.012331 | 0.981908 | 1.043697 |
| ieu-a-1127 | ER+ Breast | cg2442669 | Wald ratio | 1 | 0.015973 | 0.020346 | 0.432426 | -0.02391 | 0.055851 | 1.016101 | 0.976378 | 1.05744 |
| ieu-a-1127 | ER+ Breast | cg0038316 | Wald ratio | 1 | -0.01846 | 0.023691 | 0.435939 | -0.06489 | 0.027978 | 0.981712 | 0.937168 | 1.028373 |
| ieu-a-1127 | ER+ Breast | cg1858510 | Inverse var | 2 | 0.013724 | 0.017964 | 0.444904 | -0.02149 | 0.048934 | 1.013818 | 0.978743 | 1.050151 |
| ieu-a-1127 | ER+ Breast | cg2118777 | Wald ratio | 1 | 0.014763 | 0.019729 | 0.45428 | -0.02391 | 0.053432 | 1.014873 | 0.976378 | 1.054885 |
| ieu-a-1127 | ER+ Breast | cg1242151 | Wald ratio | 1 | 0.017136 | 0.02295 | 0.455265 | -0.02785 | 0.062118 | 1.017284 | 0.972538 | 1.064087 |
| ieu-a-1127 | ER+ Breast | cg2046983 | Wald ratio | 1 | 0.017762 | 0.023805 | 0.455574 | -0.0289 | 0.06442 | 1.017921 | 0.971518 | 1.066541 |
| ieu-a-1127 | ER+ Breast | cg0075694 | Inverse var | 2 | 0.008376 | 0.011341 | 0.460186 | -0.01385 | 0.030605 | 1.008411 | 0.986243 | 1.031078 |
| ieu-a-1127 | ER+ Breast | cg0745008 | Wald ratio | 1 | 0.011536 | 0.015645 | 0.460895 | -0.01913 | 0.0422 | 1.011603 | 0.981054 | 1.043103 |
| ieu-a-1127 | ER+ Breast | cg1378286 | Wald ratio | 1 | -0.01033 | 0.014314 | 0.47059 | -0.03838 | 0.017728 | 0.989725 | 0.962344 | 1.017886 |
| ieu-a-1127 | ER+ Breast | cg0143564 | Wald ratio | 1 | 0.015665 | 0.021828 | 0.472975 | -0.02712 | 0.058449 | 1.015788 | 0.973246 | 1.060191 |
| ieu-a-1127 | ER+ Breast | cg0775133 | Wald ratio | 1 | 0.013801 | 0.019453 | 0.478039 | -0.02433 | 0.05193 | 1.013897 | 0.975966 | 1.053302 |
| ieu-a-1127 | ER+ Breast | cg0266009 | Inverse var | 2 | 0.019388 | 0.027566 | 0.481853 | -0.03464 | 0.073418 | 1.019577 | 0.965951 | 1.07618 |
| ieu-a-1127 | ER+ Breast | cg0643576 | Wald ratio | 1 | -0.01893 | 0.027084 | 0.484534 | -0.07202 | 0.034152 | 0.981246 | 0.930515 | 1.034742 |
| ieu-a-1127 | ER+ Breast | cg0106293 | Wald ratio | 1 | 0.033643 | 0.048144 | 0.48468 | -0.06072 | 0.128005 | 1.034215 | 0.941087 | 1.136558 |
| ieu-a-1127 | ER+ Breast | cg1429561 | Wald ratio | 1 | -0.02173 | 0.031213 | 0.486302 | -0.08291 | 0.039447 | 0.978504 | 0.920436 | 1.040235 |
| ieu-a-1127 | ER+ Breast | cg1971777 | Inverse var | 2 | -0.01402 | 0.020181 | 0.487353 | -0.05357 | 0.025538 | 0.986082 | 0.94784 | 1.025867 |
| ieu-a-1127 | ER+ Breast | cg0645910 | Inverse var | 2 | 0.007516 | 0.01083 | 0.487722 | -0.01371 | 0.028743 | 1.007544 | 0.986382 | 1.02916 |
| ieu-a-1127 | ER+ Breast | cg1655409 | Inverse var | 2 | -0.01294 | 0.018803 | 0.491256 | -0.0498 | 0.023912 | 0.987141 | 0.951423 | 1.0242 |
| ieu-a-1127 | ER+ Breast | cg2506455 | Wald ratio | 1 | -0.01702 | 0.025062 | 0.49713 | -0.06614 | 0.032104 | 0.983127 | 0.936001 | 1.032625 |
| ieu-a-1127 | ER+ Breast | cg1826220 | Wald ratio | 1 | 0.01514 | 0.022311 | 0.497409 | -0.02859 | 0.05887 | 1.015255 | 0.971814 | 1.060637 |
| ieu-a-1127 | ER+ Breast | cg1040339 | Inverse var | 2 | -0.01518 | 0.023045 | 0.509995 | -0.06035 | 0.029985 | 0.984932 | 0.941434 | 1.030439 |
| ieu-a-1127 | ER+ Breast | cg2371614 | Inverse var | 2 | -0.00995 | 0.015174 | 0.512188 | -0.03969 | 0.019796 | 0.990103 | 0.961089 | 1.019993 |
| ieu-a-1127 | ER+ Breast | cg2205394 | Wald ratio | 1 | -0.01694 | 0.026005 | 0.514714 | -0.06791 | 0.034027 | 0.9832 | 0.934343 | 1.034612 |
| ieu-a-1127 | ER+ Breast | cg0481639 | Wald ratio | 1 | -0.01379 | 0.021604 | 0.52328 | -0.05613 | 0.028554 | 0.986305 | 0.945412 | 1.028966 |
| ieu-a-1127 | ER+ Breast | cg1113069 | Wald ratio | 1 | 0.023087 | 0.036369 | 0.52557 | -0.0482 | 0.094371 | 1.023355 | 0.952946 | 1.098967 |
| ieu-a-1127 | ER+ Breast | cg2471819 | Wald ratio | 1 | 0.016266 | 0.025659 | 0.526127 | -0.03403 | 0.066557 | 1.016399 | 0.966547 | 1.068822 |
| ieu-a-1127 | ER+ Breast | cg0583022 | Inverse var | 2 | 0.01077 | 0.017086 | 0.528466 | -0.02272 | 0.044259 | 1.010828 | 0.977538 | 1.045253 |

| ieu-a-1127 | ER+ Breast | cg1439192 | Inverse var | 2 | 0.008127 | 0.012985 | 0.531401 | -0.01732 | 0.033578 | 1.00816 | 0.982825 | 1.034148 |
| --- | --- | --- | --- | --- | --- | --- | --- | --- | --- | --- | --- | --- |
| ieu-a-1127 | ER+ Breast | cg2265045 | Wald ratio | 1 | -0.0091 | 0.014856 | 0.540207 | -0.03822 | 0.020019 | 0.990942 | 0.962504 | 1.02022 |
| ieu-a-1127 | ER+ Breast | cg0871263 | Wald ratio | 1 | -0.01567 | 0.025775 | 0.543116 | -0.06619 | 0.034845 | 0.984448 | 0.93595 | 1.035459 |
| ieu-a-1127 | ER+ Breast | cg1739056 | Wald ratio | 1 | 0.013247 | 0.021784 | 0.543116 | -0.02945 | 0.055943 | 1.013335 | 0.97098 | 1.057538 |
| ieu-a-1127 | ER+ Breast | cg1502903 | Wald ratio | 1 | 0.00899 | 0.01491 | 0.546548 | -0.02023 | 0.038214 | 1.009031 | 0.979969 | 1.038954 |
| ieu-a-1127 | ER+ Breast | cg0057437 | Wald ratio | 1 | 0.008127 | 0.013544 | 0.548506 | -0.01842 | 0.034674 | 1.00816 | 0.981748 | 1.035282 |
| ieu-a-1127 | ER+ Breast | cg1356140 | Wald ratio | 1 | -0.01401 | 0.023666 | 0.55396 | -0.06039 | 0.032379 | 0.986091 | 0.941396 | 1.032909 |
| ieu-a-1127 | ER+ Breast | cg0742128 | Wald ratio | 1 | 0.014321 | 0.024375 | 0.556868 | -0.03346 | 0.062096 | 1.014424 | 0.967098 | 1.064065 |
| ieu-a-1127 | ER+ Breast | cg0099552 | Wald ratio | 1 | 0.018762 | 0.031936 | 0.556868 | -0.04383 | 0.081357 | 1.01894 | 0.957114 | 1.084758 |
| ieu-a-1127 | ER+ Breast | cg1153021 | Wald ratio | 1 | -0.00777 | 0.013242 | 0.557428 | -0.03372 | 0.018185 | 0.992262 | 0.96684 | 1.018351 |
| ieu-a-1127 | ER+ Breast | cg1505906 | Wald ratio | 1 | -0.01473 | 0.025445 | 0.562625 | -0.0646 | 0.035141 | 0.985376 | 0.937438 | 1.035766 |
| ieu-a-1127 | ER+ Breast | cg0752081 | Wald ratio | 1 | -0.01795 | 0.031008 | 0.562625 | -0.07873 | 0.042823 | 0.982208 | 0.924292 | 1.043753 |
| ieu-a-1127 | ER+ Breast | cg1101474 | Wald ratio | 1 | 0.016576 | 0.028804 | 0.564972 | -0.03988 | 0.073033 | 1.016714 | 0.960904 | 1.075766 |
| ieu-a-1127 | ER+ Breast | cg2449736 | Wald ratio | 1 | -0.01494 | 0.025991 | 0.565291 | -0.06589 | 0.035997 | 0.985166 | 0.936237 | 1.036653 |
| ieu-a-1127 | ER+ Breast | cg1462438 | Wald ratio | 1 | 0.010748 | 0.019159 | 0.574814 | -0.0268 | 0.048299 | 1.010806 | 0.973552 | 1.049484 |
| ieu-a-1127 | ER+ Breast | cg1465644 | Wald ratio | 1 | 0.01125 | 0.02025 | 0.578515 | -0.02844 | 0.05094 | 1.011314 | 0.971961 | 1.05226 |
| ieu-a-1127 | ER+ Breast | cg0351996 | Wald ratio | 1 | 0.015006 | 0.027073 | 0.579383 | -0.03806 | 0.068069 | 1.015119 | 0.962658 | 1.070439 |
| ieu-a-1127 | ER+ Breast | cg2339531 | Inverse var | 2 | -0.00878 | 0.016129 | 0.586288 | -0.04039 | 0.022835 | 0.991261 | 0.960414 | 1.023098 |
| ieu-a-1127 | ER+ Breast | cg0333369 | Wald ratio | 1 | 0.012924 | 0.023837 | 0.587702 | -0.0338 | 0.059644 | 1.013007 | 0.966768 | 1.061458 |
| ieu-a-1127 | ER+ Breast | cg0720221 | Wald ratio | 1 | -0.01511 | 0.028013 | 0.58956 | -0.07002 | 0.039794 | 0.985001 | 0.932377 | 1.040596 |
| ieu-a-1127 | ER+ Breast | cg1398543 | Wald ratio | 1 | -0.01555 | 0.028821 | 0.58956 | -0.07204 | 0.04094 | 0.984572 | 0.930497 | 1.04179 |
| ieu-a-1127 | ER+ Breast | cg0143967 | Wald ratio | 1 | -0.01308 | 0.02425 | 0.58956 | -0.06061 | 0.034448 | 0.987003 | 0.941188 | 1.035048 |
| ieu-a-1127 | ER+ Breast | cg1122939 | Wald ratio | 1 | 0.008966 | 0.016629 | 0.589738 | -0.02363 | 0.041559 | 1.009007 | 0.976651 | 1.042434 |
| ieu-a-1127 | ER+ Breast | cg2562551 | Wald ratio | 1 | 0.006793 | 0.012737 | 0.593803 | -0.01817 | 0.031758 | 1.006816 | 0.981993 | 1.032267 |
| ieu-a-1127 | ER+ Breast | cg0068936 | Inverse var | 2 | 0.014185 | 0.027064 | 0.600193 | -0.03886 | 0.067231 | 1.014286 | 0.961884 | 1.069543 |
| ieu-a-1127 | ER+ Breast | cg0520410 | Wald ratio | 1 | -0.01893 | 0.036145 | 0.600411 | -0.08978 | 0.05191 | 0.981245 | 0.914136 | 1.053281 |
| ieu-a-1127 | ER+ Breast | cg0314461 | Inverse var | 2 | -0.00666 | 0.012797 | 0.602683 | -0.03174 | 0.018421 | 0.993361 | 0.968754 | 1.018592 |
| ieu-a-1127 | ER+ Breast | cg0401952 | Inverse var | 2 | -0.02036 | 0.039131 | 0.602793 | -0.09706 | 0.056333 | 0.979843 | 0.907502 | 1.05795 |
| ieu-a-1127 | ER+ Breast | cg0845863 | Wald ratio | 1 | -0.01328 | 0.025583 | 0.60377 | -0.06342 | 0.036865 | 0.986811 | 0.938549 | 1.037553 |
| ieu-a-1127 | ER+ Breast | cg2267809 | Wald ratio | 1 | 0.007578 | 0.014673 | 0.605517 | -0.02118 | 0.036337 | 1.007607 | 0.979042 | 1.037005 |
| ieu-a-1127 | ER+ Breast | cg1261648 | Wald ratio | 1 | -0.01593 | 0.031111 | 0.608718 | -0.0769 | 0.045052 | 0.9842 | 0.925979 | 1.046082 |
| ieu-a-1127 | ER+ Breast | cg1431623 | Wald ratio | 1 | -0.01734 | 0.034237 | 0.61251 | -0.08445 | 0.049764 | 0.982808 | 0.919021 | 1.051023 |
| ieu-a-1127 | ER+ Breast | cg0512245 | Wald ratio | 1 | 0.012678 | 0.02504 | 0.612626 | -0.0364 | 0.061756 | 1.012759 | 0.964255 | 1.063703 |
| ieu-a-1127 | ER+ Breast | cg1595027 | Wald ratio | 1 | -0.01058 | 0.020971 | 0.613819 | -0.05169 | 0.03052 | 0.989473 | 0.949628 | 1.030991 |
| ieu-a-1127 | ER+ Breast | cg1980239 | Wald ratio | 1 | -0.0088 | 0.017798 | 0.620949 | -0.04368 | 0.026083 | 0.991238 | 0.957256 | 1.026426 |
| ieu-a-1127 | ER+ Breast | cg0667046 | Wald ratio | 1 | 0.011205 | 0.022979 | 0.625831 | -0.03383 | 0.056243 | 1.011268 | 0.966732 | 1.057855 |
| ieu-a-1127 | ER+ Breast | cg0878796 | Wald ratio | 1 | 0.011556 | 0.023851 | 0.628005 | -0.03519 | 0.058303 | 1.011623 | 0.965421 | 1.060037 |
| ieu-a-1127 | ER+ Breast | cg2702599 | Wald ratio | 1 | -0.00657 | 0.013704 | 0.63142 | -0.03343 | 0.020285 | 0.993447 | 0.967119 | 1.020492 |

| ieu-a-1127 | ER+ Breast | cg0194029 | Wald ratio | 1 | -0.00735 | 0.015517 | 0.635725 | -0.03776 | 0.023062 | 0.992677 | 0.962942 | 1.02333 |
| --- | --- | --- | --- | --- | --- | --- | --- | --- | --- | --- | --- | --- |
| ieu-a-1127 | ER+ Breast | cg0441104 | Inverse var | 2 | -0.00764 | 0.016235 | 0.638065 | -0.03946 | 0.024183 | 0.992392 | 0.961311 | 1.024478 |
| ieu-a-1127 | ER+ Breast | cg1744925 | Wald ratio | 1 | 0.012058 | 0.025645 | 0.638213 | -0.03821 | 0.062323 | 1.012131 | 0.962514 | 1.064306 |
| ieu-a-1127 | ER+ Breast | cg2614656 | Wald ratio | 1 | 0.012343 | 0.027106 | 0.648852 | -0.04078 | 0.06547 | 1.012419 | 0.960036 | 1.06766 |
| ieu-a-1127 | ER+ Breast | cg1940636 | Wald ratio | 1 | 0.009179 | 0.020342 | 0.651831 | -0.03069 | 0.04905 | 1.009221 | 0.969774 | 1.050273 |
| ieu-a-1127 | ER+ Breast | cg1651640 | Wald ratio | 1 | 0.007201 | 0.016055 | 0.653771 | -0.02427 | 0.038668 | 1.007227 | 0.976026 | 1.039426 |
| ieu-a-1127 | ER+ Breast | cg1318517 | Wald ratio | 1 | -0.01586 | 0.035953 | 0.659085 | -0.08633 | 0.054607 | 0.984263 | 0.917291 | 1.056125 |
| ieu-a-1127 | ER+ Breast | cg2616182 | Wald ratio | 1 | 0.01381 | 0.03207 | 0.666743 | -0.04905 | 0.076667 | 1.013906 | 0.952136 | 1.079683 |
| ieu-a-1127 | ER+ Breast | cg0009944 | Wald ratio | 1 | 0.009838 | 0.022866 | 0.667026 | -0.03498 | 0.054656 | 1.009886 | 0.965625 | 1.056177 |
| ieu-a-1127 | ER+ Breast | cg0727863 | Wald ratio | 1 | -0.00946 | 0.022067 | 0.668235 | -0.05271 | 0.033794 | 0.990587 | 0.948657 | 1.034371 |
| ieu-a-1127 | ER+ Breast | cg2534794 | Wald ratio | 1 | -0.01179 | 0.027602 | 0.669404 | -0.06589 | 0.042315 | 0.988284 | 0.936238 | 1.043223 |
| ieu-a-1127 | ER+ Breast | cg0253270 | Wald ratio | 1 | -0.0075 | 0.017769 | 0.672863 | -0.04233 | 0.027325 | 0.992526 | 0.958554 | 1.027702 |
| ieu-a-1127 | ER+ Breast | cg2179125 | Wald ratio | 1 | 0.009132 | 0.021744 | 0.674485 | -0.03349 | 0.05175 | 1.009174 | 0.967069 | 1.053113 |
| ieu-a-1127 | ER+ Breast | cg1325742 | Inverse var | 2 | -0.01113 | 0.026732 | 0.677199 | -0.06352 | 0.041267 | 0.988933 | 0.938452 | 1.04213 |
| ieu-a-1127 | ER+ Breast | cg2610243 | Wald ratio | 1 | 0.008181 | 0.019929 | 0.68142 | -0.03088 | 0.047241 | 1.008215 | 0.969593 | 1.048375 |
| ieu-a-1127 | ER+ Breast | cg2368144 | Wald ratio | 1 | -0.01145 | 0.028024 | 0.682943 | -0.06637 | 0.043481 | 0.988619 | 0.93578 | 1.044441 |
| ieu-a-1127 | ER+ Breast | cg1569357 | Wald ratio | 1 | 0.00772 | 0.019059 | 0.685431 | -0.02964 | 0.045076 | 1.00775 | 0.970799 | 1.046107 |
| ieu-a-1127 | ER+ Breast | cg0709071 | Inverse var | 2 | 0.018538 | 0.045772 | 0.685472 | -0.07117 | 0.108251 | 1.018711 | 0.931299 | 1.114327 |
| ieu-a-1127 | ER+ Breast | cg2069842 | Inverse var | 2 | -0.00556 | 0.014049 | 0.692084 | -0.0331 | 0.021973 | 0.994452 | 0.967441 | 1.022216 |
| ieu-a-1127 | ER+ Breast | cg2204141 | Wald ratio | 1 | 0.006534 | 0.016515 | 0.692397 | -0.02584 | 0.038904 | 1.006555 | 0.974494 | 1.03967 |
| ieu-a-1127 | ER+ Breast | cg0066855 | Wald ratio | 1 | -0.00405 | 0.010473 | 0.699003 | -0.02458 | 0.016478 | 0.995958 | 0.975722 | 1.016615 |
| ieu-a-1127 | ER+ Breast | cg1578774 | Wald ratio | 1 | -0.00744 | 0.019236 | 0.699003 | -0.04514 | 0.030264 | 0.99259 | 0.955864 | 1.030727 |
| ieu-a-1127 | ER+ Breast | cg2436109 | Wald ratio | 1 | -0.00676 | 0.017623 | 0.701185 | -0.0413 | 0.027779 | 0.993261 | 0.959538 | 1.028168 |
| ieu-a-1127 | ER+ Breast | cg0136060 | Wald ratio | 1 | 0.015174 | 0.0396 | 0.701588 | -0.06244 | 0.092791 | 1.01529 | 0.939467 | 1.097232 |
| ieu-a-1127 | ER+ Breast | cg0305188 | Wald ratio | 1 | -0.00844 | 0.022158 | 0.703239 | -0.05187 | 0.034988 | 0.991594 | 0.949452 | 1.035607 |
| ieu-a-1127 | ER+ Breast | cg0144782 | Wald ratio | 1 | -0.00635 | 0.016796 | 0.70552 | -0.03927 | 0.026573 | 0.993673 | 0.961495 | 1.026929 |
| ieu-a-1127 | ER+ Breast | cg2040840 | Wald ratio | 1 | -0.00816 | 0.021763 | 0.70766 | -0.05082 | 0.034494 | 0.991872 | 0.950453 | 1.035096 |
| ieu-a-1127 | ER+ Breast | cg2404949 | Wald ratio | 1 | 0.007243 | 0.019659 | 0.712559 | -0.03129 | 0.045773 | 1.007269 | 0.969196 | 1.046837 |
| ieu-a-1127 | ER+ Breast | cg2672870 | Wald ratio | 1 | 0.005953 | 0.016237 | 0.713868 | -0.02587 | 0.037777 | 1.005971 | 0.974461 | 1.0385 |
| ieu-a-1127 | ER+ Breast | cg0587778 | Wald ratio | 1 | -0.00458 | 0.012508 | 0.714474 | -0.02909 | 0.019939 | 0.995434 | 0.971328 | 1.020139 |
| ieu-a-1127 | ER+ Breast | cg1698358 | Wald ratio | 1 | 0.009785 | 0.028227 | 0.728842 | -0.04554 | 0.065111 | 1.009834 | 0.955481 | 1.067278 |
| ieu-a-1127 | ER+ Breast | cg0518353 | Wald ratio | 1 | 0.014257 | 0.041242 | 0.729584 | -0.06658 | 0.095092 | 1.014359 | 0.93559 | 1.09976 |
| ieu-a-1127 | ER+ Breast | cg2169831 | Wald ratio | 1 | -0.00853 | 0.024961 | 0.732522 | -0.05745 | 0.040392 | 0.991505 | 0.944166 | 1.041219 |
| ieu-a-1127 | ER+ Breast | cg0237310 | Wald ratio | 1 | 0.005266 | 0.015799 | 0.738883 | -0.0257 | 0.036232 | 1.00528 | 0.974628 | 1.036896 |
| ieu-a-1127 | ER+ Breast | cg2316149 | Wald ratio | 1 | 0.011866 | 0.036531 | 0.745319 | -0.05973 | 0.083467 | 1.011937 | 0.942014 | 1.087049 |
| ieu-a-1127 | ER+ Breast | cg2612687 | Wald ratio | 1 | 0.003876 | 0.012059 | 0.747886 | -0.01976 | 0.027511 | 1.003883 | 0.980435 | 1.027893 |
| ieu-a-1127 | ER+ Breast | cg1595504 | Wald ratio | 1 | 0.005135 | 0.016291 | 0.752597 | -0.02679 | 0.037065 | 1.005148 | 0.973561 | 1.03776 |
| ieu-a-1127 | ER+ Breast | cg0963595 | Inverse var | 2 | -0.00536 | 0.017075 | 0.753639 | -0.03883 | 0.028108 | 0.994655 | 0.961918 | 1.028507 |

| ieu-a-1127 | ER+ Breast | cg0480656 | Wald ratio | 1 | -0.01237 | 0.039647 | 0.755131 | -0.09007 | 0.065342 | 0.987711 | 0.913865 | 1.067525 |
| --- | --- | --- | --- | --- | --- | --- | --- | --- | --- | --- | --- | --- |
| ieu-a-1127 | ER+ Breast | cg1945280 | Wald ratio | 1 | 0.007322 | 0.02349 | 0.755277 | -0.03872 | 0.053362 | 1.007348 | 0.962021 | 1.054811 |
| ieu-a-1127 | ER+ Breast | cg1788467 | Wald ratio | 1 | 0.005832 | 0.01879 | 0.756299 | -0.031 | 0.042661 | 1.005849 | 0.969478 | 1.043584 |
| ieu-a-1127 | ER+ Breast | cg2005937 | Wald ratio | 1 | 0.003195 | 0.010446 | 0.759694 | -0.01728 | 0.02367 | 1.0032 | 0.982869 | 1.023952 |
| ieu-a-1127 | ER+ Breast | cg1106620 | Wald ratio | 1 | -0.0056 | 0.018428 | 0.761282 | -0.04172 | 0.03052 | 0.994417 | 0.959141 | 1.030991 |
| ieu-a-1127 | ER+ Breast | cg0132847 | Wald ratio | 1 | -0.00955 | 0.031939 | 0.764964 | -0.07215 | 0.053052 | 0.990497 | 0.930391 | 1.054485 |
| ieu-a-1127 | ER+ Breast | cg0854855 | Wald ratio | 1 | 0.004306 | 0.014485 | 0.76624 | -0.02408 | 0.032698 | 1.004316 | 0.976203 | 1.033238 |
| ieu-a-1127 | ER+ Breast | cg1071731 | Inverse var | 2 | 0.004289 | 0.014821 | 0.772266 | -0.02476 | 0.033338 | 1.004299 | 0.975544 | 1.0339 |
| ieu-a-1127 | ER+ Breast | cg2312634 | Wald ratio | 1 | -0.00787 | 0.027711 | 0.776341 | -0.06219 | 0.046442 | 0.992158 | 0.939707 | 1.047537 |
| ieu-a-1127 | ER+ Breast | cg1813207 | Wald ratio | 1 | 0.005796 | 0.020548 | 0.777904 | -0.03448 | 0.04607 | 1.005812 | 0.966109 | 1.047147 |
| ieu-a-1127 | ER+ Breast | cg2120165 | Wald ratio | 1 | -0.0068 | 0.024253 | 0.779103 | -0.05434 | 0.040734 | 0.99322 | 0.947111 | 1.041575 |
| ieu-a-1127 | ER+ Breast | cg0961159 | Wald ratio | 1 | 0.002634 | 0.010195 | 0.79615 | -0.01735 | 0.022617 | 1.002637 | 0.9828 | 1.022875 |
| ieu-a-1127 | ER+ Breast | cg0920629 | Wald ratio | 1 | -0.00807 | 0.031464 | 0.797634 | -0.06974 | 0.053602 | 0.991965 | 0.932638 | 1.055065 |
| ieu-a-1127 | ER+ Breast | cg2671821 | Wald ratio | 1 | 0.004809 | 0.019236 | 0.802587 | -0.03289 | 0.042511 | 1.00482 | 0.967642 | 1.043427 |
| ieu-a-1127 | ER+ Breast | cg0360943 | Wald ratio | 1 | -0.00774 | 0.03208 | 0.809261 | -0.07062 | 0.055133 | 0.992287 | 0.931816 | 1.056681 |
| ieu-a-1127 | ER+ Breast | cg2091220 | Wald ratio | 1 | 0.00545 | 0.022949 | 0.812269 | -0.03953 | 0.05043 | 1.005465 | 0.961242 | 1.051724 |
| ieu-a-1127 | ER+ Breast | cg1280306 | Wald ratio | 1 | 0.00592 | 0.025654 | 0.817494 | -0.04436 | 0.056201 | 1.005938 | 0.956608 | 1.057811 |
| ieu-a-1127 | ER+ Breast | cg2213278 | Wald ratio | 1 | 0.005515 | 0.023897 | 0.817494 | -0.04132 | 0.052353 | 1.00553 | 0.959519 | 1.053748 |
| ieu-a-1127 | ER+ Breast | cg0466646 | Wald ratio | 1 | 0.002396 | 0.010613 | 0.821352 | -0.0184 | 0.023198 | 1.002399 | 0.981764 | 1.023469 |
| ieu-a-1127 | ER+ Breast | cg2659998 | Wald ratio | 1 | -0.00518 | 0.023008 | 0.821895 | -0.05027 | 0.039916 | 0.994834 | 0.950968 | 1.040724 |
| ieu-a-1127 | ER+ Breast | cg1109502 | Wald ratio | 1 | -0.00446 | 0.019794 | 0.821895 | -0.04325 | 0.034341 | 0.995554 | 0.957669 | 1.034938 |
| ieu-a-1127 | ER+ Breast | cg0348093 | Wald ratio | 1 | 0.004814 | 0.021396 | 0.821979 | -0.03712 | 0.04675 | 1.004826 | 0.963558 | 1.047861 |
| ieu-a-1127 | ER+ Breast | cg0206869 | Wald ratio | 1 | 0.008854 | 0.040625 | 0.827469 | -0.07077 | 0.088479 | 1.008893 | 0.931675 | 1.092511 |
| ieu-a-1127 | ER+ Breast | cg2135671 | Wald ratio | 1 | -0.00365 | 0.017119 | 0.831067 | -0.03721 | 0.029902 | 0.996355 | 0.963478 | 1.030353 |
| ieu-a-1127 | ER+ Breast | cg1513045 | Wald ratio | 1 | 0.006397 | 0.030917 | 0.836091 | -0.0542 | 0.066994 | 1.006417 | 0.947242 | 1.069289 |
| ieu-a-1127 | ER+ Breast | cg2449022 | Wald ratio | 1 | -0.00865 | 0.041858 | 0.83627 | -0.09069 | 0.073391 | 0.991387 | 0.913298 | 1.076152 |
| ieu-a-1127 | ER+ Breast | cg2381325 | Wald ratio | 1 | 0.006386 | 0.031931 | 0.841481 | -0.0562 | 0.068971 | 1.006407 | 0.945352 | 1.071405 |
| ieu-a-1127 | ER+ Breast | cg0830553 | Wald ratio | 1 | -0.00547 | 0.027329 | 0.841481 | -0.05903 | 0.048099 | 0.994549 | 0.942678 | 1.049274 |
| ieu-a-1127 | ER+ Breast | cg2761893 | Wald ratio | 1 | 0.005673 | 0.028633 | 0.842956 | -0.05045 | 0.061794 | 1.005689 | 0.950803 | 1.063743 |
| ieu-a-1127 | ER+ Breast | cg2191388 | Wald ratio | 1 | -0.00346 | 0.017739 | 0.845459 | -0.03823 | 0.031311 | 0.996548 | 0.962495 | 1.031806 |
| ieu-a-1127 | ER+ Breast | cg2690099 | Wald ratio | 1 | -0.00545 | 0.028557 | 0.848597 | -0.06142 | 0.050519 | 0.994563 | 0.940426 | 1.051817 |
| ieu-a-1127 | ER+ Breast | cg1758061 | Wald ratio | 1 | -0.00262 | 0.013867 | 0.85018 | -0.0298 | 0.024561 | 0.997384 | 0.97064 | 1.024865 |
| ieu-a-1127 | ER+ Breast | cg1488007 | Wald ratio | 1 | -0.00675 | 0.036522 | 0.8534 | -0.07833 | 0.064835 | 0.993274 | 0.924657 | 1.066983 |
| ieu-a-1127 | ER+ Breast | cg0619775 | Wald ratio | 1 | 0.003081 | 0.016945 | 0.855725 | -0.03013 | 0.036294 | 1.003086 | 0.970317 | 1.036961 |
| ieu-a-1127 | ER+ Breast | cg2285850 | Wald ratio | 1 | -0.00376 | 0.020764 | 0.856117 | -0.04446 | 0.036933 | 0.996242 | 0.956511 | 1.037624 |
| ieu-a-1127 | ER+ Breast | cg1083528 | Wald ratio | 1 | -0.00208 | 0.011562 | 0.857555 | -0.02474 | 0.020587 | 0.997927 | 0.975566 | 1.0208 |
| ieu-a-1127 | ER+ Breast | cg0002286 | Wald ratio | 1 | 0.001462 | 0.008352 | 0.86108 | -0.01491 | 0.017833 | 1.001463 | 0.985201 | 1.017992 |
| ieu-a-1127 | ER+ Breast | cg1641615 | Wald ratio | 1 | 0.002889 | 0.016561 | 0.861536 | -0.02957 | 0.035348 | 1.002893 | 0.970862 | 1.03598 |

| ieu-a-1127 | ER+ Breast | cg0718064 | Wald ratio | 1 | -0.00443 | 0.02577 | 0.863611 | -0.05494 | 0.046082 | 0.995583 | 0.946546 | 1.04716 |
| --- | --- | --- | --- | --- | --- | --- | --- | --- | --- | --- | --- | --- |
| ieu-a-1127 | ER+ Breast | cg0559366 | Wald ratio | 1 | 0.002542 | 0.014987 | 0.865332 | -0.02683 | 0.031916 | 1.002545 | 0.973524 | 1.032431 |
| ieu-a-1127 | ER+ Breast | cg0847279 | Wald ratio | 1 | 0.003597 | 0.021823 | 0.869074 | -0.03918 | 0.046369 | 1.003604 | 0.961582 | 1.047461 |
| ieu-a-1127 | ER+ Breast | cg0147137 | Inverse var | 2 | -0.00567 | 0.03565 | 0.873654 | -0.07554 | 0.064206 | 0.994347 | 0.927239 | 1.066312 |
| ieu-a-1127 | ER+ Breast | cg2564982 | Wald ratio | 1 | 0.006768 | 0.043147 | 0.875353 | -0.0778 | 0.091337 | 1.006791 | 0.925149 | 1.095638 |
| ieu-a-1127 | ER+ Breast | cg0803532 | Wald ratio | 1 | 0.005017 | 0.032034 | 0.875539 | -0.05777 | 0.067804 | 1.00503 | 0.943868 | 1.070155 |
| ieu-a-1127 | ER+ Breast | cg1095972 | Wald ratio | 1 | 0.004517 | 0.030491 | 0.882226 | -0.05525 | 0.06428 | 1.004527 | 0.946253 | 1.066391 |
| ieu-a-1127 | ER+ Breast | cg0738375 | Wald ratio | 1 | 0.005695 | 0.038669 | 0.882906 | -0.0701 | 0.081487 | 1.005712 | 0.932304 | 1.084899 |
| ieu-a-1127 | ER+ Breast | cg1013008 | Wald ratio | 1 | 0.005425 | 0.038264 | 0.887245 | -0.06957 | 0.080423 | 1.00544 | 0.932793 | 1.083745 |
| ieu-a-1127 | ER+ Breast | cg0886739 | Inverse var | 3 | 0.00222 | 0.015739 | 0.887828 | -0.02863 | 0.033069 | 1.002223 | 0.971777 | 1.033622 |
| ieu-a-1127 | ER+ Breast | cg1664605 | Wald ratio | 1 | -0.00287 | 0.020472 | 0.888383 | -0.043 | 0.037251 | 0.997131 | 0.957914 | 1.037954 |
| ieu-a-1127 | ER+ Breast | cg1085867 | Wald ratio | 1 | -0.00342 | 0.024765 | 0.890295 | -0.05196 | 0.045124 | 0.99659 | 0.949371 | 1.046157 |
| ieu-a-1127 | ER+ Breast | cg0965849 | Wald ratio | 1 | 0.001851 | 0.015631 | 0.905734 | -0.02879 | 0.032489 | 1.001853 | 0.971624 | 1.033022 |
| ieu-a-1127 | ER+ Breast | cg0686173 | Wald ratio | 1 | -0.0039 | 0.033931 | 0.908491 | -0.07041 | 0.062605 | 0.996107 | 0.932016 | 1.064607 |
| ieu-a-1127 | ER+ Breast | cg0068922 | Wald ratio | 1 | -0.00247 | 0.021476 | 0.908491 | -0.04456 | 0.039625 | 0.997535 | 0.956416 | 1.04042 |
| ieu-a-1127 | ER+ Breast | cg0454596 | Wald ratio | 1 | -0.00212 | 0.018448 | 0.908491 | -0.03828 | 0.034037 | 0.997882 | 0.962445 | 1.034623 |
| ieu-a-1127 | ER+ Breast | cg0591403 | Inverse var | 2 | -0.00179 | 0.016967 | 0.916192 | -0.03504 | 0.03147 | 0.998216 | 0.965565 | 1.031971 |
| ieu-a-1127 | ER+ Breast | cg0794110 | Wald ratio | 1 | -0.00306 | 0.030921 | 0.921217 | -0.06366 | 0.057547 | 0.996947 | 0.938321 | 1.059235 |
| ieu-a-1127 | ER+ Breast | cg0783931 | Wald ratio | 1 | -0.00268 | 0.028265 | 0.924524 | -0.05808 | 0.052722 | 0.997326 | 0.943576 | 1.054137 |
| ieu-a-1127 | ER+ Breast | cg1533130 | Wald ratio | 1 | -0.00281 | 0.030154 | 0.925885 | -0.06191 | 0.056297 | 0.997199 | 0.93997 | 1.057912 |
| ieu-a-1127 | ER+ Breast | cg1853322 | Wald ratio | 1 | -0.00155 | 0.01667 | 0.926076 | -0.03422 | 0.031126 | 0.998455 | 0.96636 | 1.031615 |
| ieu-a-1127 | ER+ Breast | cg0963915 | Wald ratio | 1 | -0.00159 | 0.017234 | 0.926351 | -0.03537 | 0.032185 | 0.998408 | 0.965247 | 1.032709 |
| ieu-a-1127 | ER+ Breast | cg1792731 | Wald ratio | 1 | 0.002221 | 0.024746 | 0.928491 | -0.04628 | 0.050723 | 1.002223 | 0.954773 | 1.052032 |
| ieu-a-1127 | ER+ Breast | cg0322200 | Wald ratio | 1 | 0.001959 | 0.022037 | 0.92917 | -0.04123 | 0.045152 | 1.001961 | 0.959604 | 1.046187 |
| ieu-a-1127 | ER+ Breast | cg2696327 | Wald ratio | 1 | 0.002002 | 0.027361 | 0.94167 | -0.05162 | 0.055629 | 1.002004 | 0.949685 | 1.057205 |
| ieu-a-1127 | ER+ Breast | cg2186960 | Wald ratio | 1 | 0.000828 | 0.011456 | 0.942372 | -0.02163 | 0.023282 | 1.000829 | 0.978606 | 1.023555 |
| ieu-a-1127 | ER+ Breast | cg0129432 | Wald ratio | 1 | 0.000745 | 0.010312 | 0.942372 | -0.01947 | 0.020957 | 1.000746 | 0.980722 | 1.021178 |
| ieu-a-1127 | ER+ Breast | cg1533924 | Wald ratio | 1 | 0.001851 | 0.027452 | 0.946251 | -0.05196 | 0.055657 | 1.001852 | 0.949371 | 1.057235 |
| ieu-a-1127 | ER+ Breast | cg1593707 | Wald ratio | 1 | 0.000779 | 0.01169 | 0.946847 | -0.02213 | 0.023691 | 1.00078 | 0.978111 | 1.023974 |
| ieu-a-1127 | ER+ Breast | cg2376251 | Wald ratio | 1 | 0.000682 | 0.010233 | 0.946847 | -0.01938 | 0.02074 | 1.000682 | 0.980811 | 1.020956 |
| ieu-a-1127 | ER+ Breast | cg2603858 | Wald ratio | 1 | 0.000746 | 0.011184 | 0.946847 | -0.02118 | 0.022666 | 1.000746 | 0.979048 | 1.022925 |
| ieu-a-1127 | ER+ Breast | cg0426370 | Inverse var | 2 | -0.00088 | 0.013315 | 0.947106 | -0.02698 | 0.025213 | 0.999117 | 0.973381 | 1.025534 |
| ieu-a-1127 | ER+ Breast | cg1508480 | Wald ratio | 1 | 0.000991 | 0.016145 | 0.951038 | -0.03065 | 0.032636 | 1.000992 | 0.969812 | 1.033174 |
| ieu-a-1127 | ER+ Breast | cg2166428 | Wald ratio | 1 | -0.00074 | 0.012242 | 0.951673 | -0.02474 | 0.023252 | 0.999258 | 0.975567 | 1.023525 |
| ieu-a-1127 | ER+ Breast | cg1038107 | Wald ratio | 1 | 0.000897 | 0.016332 | 0.956182 | -0.03111 | 0.032907 | 1.000898 | 0.969366 | 1.033455 |
| ieu-a-1127 | ER+ Breast | cg0423297 | Wald ratio | 1 | -0.00121 | 0.022367 | 0.956892 | -0.04505 | 0.042631 | 0.998792 | 0.955951 | 1.043553 |
| ieu-a-1127 | ER+ Breast | cg1844633 | Wald ratio | 1 | 0.001044 | 0.019838 | 0.958025 | -0.03784 | 0.039927 | 1.001045 | 0.962868 | 1.040735 |
| ieu-a-1127 | ER+ Breast | cg0647882 | Wald ratio | 1 | 0.000768 | 0.014843 | 0.958749 | -0.02833 | 0.029861 | 1.000768 | 0.972072 | 1.030311 |

| ieu-a-1127 | ER+ Breast | cg0688061 | Wald ratio | 1 | 0.001882 | 0.037647 | 0.960122 | -0.07191 | 0.075671 | 1.001884 | 0.930618 | 1.078607 |
| --- | --- | --- | --- | --- | --- | --- | --- | --- | --- | --- | --- | --- |
| ieu-a-1127 | ER+ Breast | cg2450379 | Wald ratio | 1 | -0.00088 | 0.01781 | 0.960614 | -0.03579 | 0.034028 | 0.999121 | 0.964846 | 1.034614 |
| ieu-a-1127 | ER+ Breast | cg1435708 | Wald ratio | 1 | -0.001 | 0.020483 | 0.960989 | -0.04115 | 0.039145 | 0.998999 | 0.959686 | 1.039921 |
| ieu-a-1127 | ER+ Breast | cg0058049 | Wald ratio | 1 | 0.000775 | 0.016276 | 0.96202 | -0.03113 | 0.032676 | 1.000775 | 0.969354 | 1.033216 |
| ieu-a-1127 | ER+ Breast | cg1261950 | Wald ratio | 1 | -0.00111 | 0.023677 | 0.962466 | -0.04752 | 0.045292 | 0.998886 | 0.953591 | 1.046334 |
| ieu-a-1127 | ER+ Breast | cg0554268 | Wald ratio | 1 | 0.000645 | 0.01386 | 0.962902 | -0.02652 | 0.02781 | 1.000645 | 0.973828 | 1.0282 |
| ieu-a-1127 | ER+ Breast | cg1228925 | Wald ratio | 1 | -0.00092 | 0.023445 | 0.968806 | -0.04687 | 0.045035 | 0.999084 | 0.954213 | 1.046064 |
| ieu-a-1127 | ER+ Breast | cg0017724 | Wald ratio | 1 | 0.000546 | 0.013963 | 0.968806 | -0.02682 | 0.027913 | 1.000546 | 0.973536 | 1.028306 |
| ieu-a-1127 | ER+ Breast | cg2290586 | Inverse var | 2 | -0.00061 | 0.015904 | 0.969419 | -0.03178 | 0.030562 | 0.99939 | 0.968718 | 1.031034 |
| ieu-a-1127 | ER+ Breast | cg1417422 | Wald ratio | 1 | -0.00066 | 0.017525 | 0.970086 | -0.03501 | 0.033691 | 0.999343 | 0.9656 | 1.034265 |
| ieu-a-1127 | ER+ Breast | cg0355433 | Wald ratio | 1 | -0.00098 | 0.027967 | 0.972172 | -0.05579 | 0.053841 | 0.999025 | 0.945736 | 1.055316 |
| ieu-a-1127 | ER+ Breast | cg0559910 | Wald ratio | 1 | -0.00057 | 0.022405 | 0.979544 | -0.04449 | 0.04334 | 0.999426 | 0.956486 | 1.044293 |
| ieu-a-1127 | ER+ Breast | cg0572022 | Wald ratio | 1 | -0.00054 | 0.026638 | 0.983718 | -0.05275 | 0.051666 | 0.999457 | 0.948614 | 1.053024 |
| ieu-a-1127 | ER+ Breast | cg2635196 | Inverse var | 2 | 0.000307 | 0.01512 | 0.983798 | -0.02933 | 0.029943 | 1.000307 | 0.971097 | 1.030395 |
| ieu-a-1127 | ER+ Breast | cg0378979 | Wald ratio | 1 | -0.00041 | 0.021717 | 0.984946 | -0.04297 | 0.042155 | 0.99959 | 0.957936 | 1.043056 |
| ieu-a-1127 | ER+ Breast | cg1058566 | Inverse var | 2 | 0.000343 | 0.020107 | 0.986392 | -0.03907 | 0.039752 | 1.000343 | 0.961687 | 1.040553 |
| ieu-a-1127 | ER+ Breast | cg0947924 | Inverse var | 2 | 0.000433 | 0.036956 | 0.990658 | -0.072 | 0.072867 | 1.000433 | 0.930529 | 1.075588 |
| ieu-a-1127 | ER+ Breast | cg0250874 | Wald ratio | 1 | -0.00024 | 0.022379 | 0.991328 | -0.04411 | 0.04362 | 0.999757 | 0.956852 | 1.044585 |
| ieu-a-1127 | ER+ Breast | cg0203255 | Wald ratio | 1 | 0 | 0.023188 | 1 | -0.04545 | 0.045449 | 1 | 0.955569 | 1.046497 |
| ieu-a-1127 | ER+ Breast | cg0944762 | Wald ratio | 1 | 0 | 0.027518 | 1 | -0.05394 | 0.053936 | 1 | 0.947493 | 1.055417 |
