# Supplementary material for: Smoking, DNA Methylation, and Breast Cancer: A Mendelian Randomization Study
Source: Front Oncol. 2021 Sep 28;11:745918. doi: 10.3389/fonc.2021.745918 (PMC8507148; doi:10.3389/fonc.2021.745918)
Supplement: Supplementary file 4 [file Table_3.docx]

Table S3. Two-sample MR results for smoking-related CpG sites on the risk of ER- breast cancer.

| id.outcome | outcome | exposure | method | nsnp | b | se | pval | lo_ci | up_ci | or | or_lci95 | or_uci95 |
| --- | --- | --- | --- | --- | --- | --- | --- | --- | --- | --- | --- | --- |
| ieu-a-1128 | ER- Breast | cg06321596 | Wald ratio | 1 | -0.03337 | 0.029432 | 0.256854 | -0.09106 | 0.024315 | 0.967179 | 0.912964 | 1.024613 |
| ieu-a-1128 | ER- Breast | cg14391586 | Wald ratio | 1 | -0.0431 | 0.020322 | 0.033948 | -0.08293 | -0.00327 | 0.957818 | 0.920416 | 0.99674 |
| ieu-a-1128 | ER- Breast | cg25741533 | Wald ratio | 1 | -0.02824 | 0.028483 | 0.321394 | -0.08407 | 0.027583 | 0.972152 | 0.919368 | 1.027966 |
| ieu-a-1128 | ER- Breast | cg15937073 | Wald ratio | 1 | -0.00966 | 0.017924 | 0.589797 | -0.04479 | 0.025468 | 0.990383 | 0.956194 | 1.025795 |
| ieu-a-1128 | ER- Breast | cg05830220 | IVW | 2 | -0.00673 | 0.026217 | 0.79739 | -0.05812 | 0.044655 | 0.993292 | 0.94354 | 1.045667 |
| ieu-a-1128 | ER- Breast | cg24249791 | Wald ratio | 1 | 0.007257 | 0.016188 | 0.653954 | -0.02447 | 0.038986 | 1.007283 | 0.975825 | 1.039756 |
| ieu-a-1128 | ER- Breast | cg11902777 | Wald ratio | 1 | -0.05792 | 0.037093 | 0.118429 | -0.13062 | 0.014785 | 0.943728 | 0.877551 | 1.014895 |
| ieu-a-1128 | ER- Breast | cg09006487 | Wald ratio | 1 | -0.04918 | 0.038827 | 0.205275 | -0.12528 | 0.02692 | 0.952009 | 0.882249 | 1.027285 |
| ieu-a-1128 | ER- Breast | cg13039251 | Wald ratio | 1 | 0.023199 | 0.035921 | 0.518387 | -0.04721 | 0.093605 | 1.02347 | 0.95389 | 1.098126 |
| ieu-a-1128 | ER- Breast | cg22642495 | Wald ratio | 1 | -0.04215 | 0.049174 | 0.391366 | -0.13853 | 0.054231 | 0.958727 | 0.870638 | 1.055729 |
| ieu-a-1128 | ER- Breast | cg15130459 | Wald ratio | 1 | 0.045949 | 0.048507 | 0.34351 | -0.04913 | 0.141023 | 1.047021 | 0.952061 | 1.151452 |
| ieu-a-1128 | ER- Breast | cg07502868 | Wald ratio | 1 | -0.00994 | 0.03702 | 0.788241 | -0.0825 | 0.062616 | 0.990106 | 0.920809 | 1.064618 |
| ieu-a-1128 | ER- Breast | cg19956914 | Wald ratio | 1 | 0.01106 | 0.041167 | 0.788194 | -0.06963 | 0.091748 | 1.011121 | 0.93274 | 1.096089 |
| ieu-a-1128 | ER- Breast | cg00383167 | Wald ratio | 1 | -0.01377 | 0.036364 | 0.704845 | -0.08505 | 0.057499 | 0.98632 | 0.918469 | 1.059184 |
| ieu-a-1128 | ER- Breast | cg06435765 | Wald ratio | 1 | -0.02998 | 0.041283 | 0.467768 | -0.11089 | 0.050939 | 0.970468 | 0.895036 | 1.052258 |
| ieu-a-1128 | ER- Breast | cg13985437 | Wald ratio | 1 | 0.017823 | 0.043989 | 0.685351 | -0.0684 | 0.104042 | 1.017983 | 0.933891 | 1.109648 |
| ieu-a-1128 | ER- Breast | cg24033122 | Wald ratio | 1 | 0.039476 | 0.016701 | 0.018097 | 0.006741 | 0.07221 | 1.040265 | 1.006764 | 1.074881 |
| ieu-a-1128 | ER- Breast | cg05593667 | Wald ratio | 1 | -0.00964 | 0.022962 | 0.674597 | -0.05465 | 0.035365 | 0.990406 | 0.94682 | 1.035998 |
| ieu-a-1128 | ER- Breast | cg17823346 | Wald ratio | 1 | 0.048933 | 0.020318 | 0.016026 | 0.009109 | 0.088757 | 1.05015 | 1.009151 | 1.092815 |
| ieu-a-1128 | ER- Breast | cg25313468 | IVW | 2 | 0.001897 | 0.025942 | 0.941695 | -0.04895 | 0.052743 | 1.001899 | 0.95223 | 1.054159 |
| ieu-a-1128 | ER- Breast | cg03864215 | Wald ratio | 1 | -0.01426 | 0.029794 | 0.6322 | -0.07266 | 0.044135 | 0.985841 | 0.929921 | 1.045124 |
| ieu-a-1128 | ER- Breast | cg26900995 | Wald ratio | 1 | 0.04621 | 0.043354 | 0.286483 | -0.03876 | 0.131184 | 1.047294 | 0.961977 | 1.140177 |
| ieu-a-1128 | ER- Breast | cg15693483 | Wald ratio | 1 | 0.004705 | 0.015417 | 0.760218 | -0.02551 | 0.034922 | 1.004716 | 0.974811 | 1.035539 |
| ieu-a-1128 | ER- Breast | cg05914034 | IVW | 2 | 0.011839 | 0.025908 | 0.647704 | -0.03894 | 0.062618 | 1.011909 | 0.961808 | 1.06462 |
| ieu-a-1128 | ER- Breast | cg01314044 | Wald ratio | 1 | -0.05872 | 0.029487 | 0.046447 | -0.11651 | -0.00092 | 0.942973 | 0.890019 | 0.999077 |
| ieu-a-1128 | ER- Breast | cg13185177 | Wald ratio | 1 | -0.0007 | 0.054635 | 0.989705 | -0.10779 | 0.10638 | 0.999295 | 0.897816 | 1.112244 |
| ieu-a-1128 | ER- Breast | cg12803068 | Wald ratio | 1 | 0.020227 | 0.038727 | 0.601466 | -0.05568 | 0.096132 | 1.020433 | 0.945843 | 1.100905 |
| ieu-a-1128 | ER- Breast | cg21356710 | Wald ratio | 1 | 0.041771 | 0.026021 | 0.108436 | -0.00923 | 0.092773 | 1.042656 | 0.990812 | 1.097213 |
| ieu-a-1128 | ER- Breast | cg01268763 | Wald ratio | 1 | -0.04827 | 0.036614 | 0.187346 | -0.12004 | 0.023489 | 0.952872 | 0.886887 | 1.023767 |
| ieu-a-1128 | ER- Breast | cg10012512 | Wald ratio | 1 | 0.046875 | 0.03404 | 0.168497 | -0.01984 | 0.113594 | 1.047991 | 0.980352 | 1.120297 |
| ieu-a-1128 | ER- Breast | cg04232972 | Wald ratio | 1 | -0.02454 | 0.034216 | 0.47318 | -0.09161 | 0.04252 | 0.975755 | 0.912464 | 1.043437 |
| ieu-a-1128 | ER- Breast | cg01439670 | Wald ratio | 1 | 0.014997 | 0.037013 | 0.685351 | -0.05755 | 0.087543 | 1.01511 | 0.944075 | 1.091489 |
| ieu-a-1128 | ER- Breast | cg06197751 | Wald ratio | 1 | -0.03235 | 0.025968 | 0.212852 | -0.08325 | 0.018548 | 0.968167 | 0.920123 | 1.018721 |
| ieu-a-1128 | ER- Breast | cg10130088 | Wald ratio | 1 | -0.02827 | 0.057967 | 0.625773 | -0.14188 | 0.085346 | 0.972126 | 0.867721 | 1.089093 |
| ieu-a-1128 | ER- Breast | cg22053945 | Wald ratio | 1 | -0.03132 | 0.039795 | 0.431207 | -0.10932 | 0.046675 | 0.969162 | 0.896441 | 1.047781 |
| ieu-a-1128 | ER- Breast | cg19197419 | Wald ratio | 1 | -0.02371 | 0.03399 | 0.485503 | -0.09033 | 0.042913 | 0.976571 | 0.913631 | 1.043848 |
| ieu-a-1128 | ER- Breast | cg04411044 | IVW | 2 | -0.01861 | 0.02493 | 0.455353 | -0.06747 | 0.030252 | 0.981561 | 0.934752 | 1.030715 |

| ieu-a-1128 | ER- Breast | cg17927313 | Wald ratio | 1 | 0.036168 | 0.038388 | 0.346116 | -0.03907 | 0.111409 | 1.03683 | 0.96168 | 1.117852 |
| --- | --- | --- | --- | --- | --- | --- | --- | --- | --- | --- | --- | --- |
| ieu-a-1128 | ER- Breast | cg00806481 | Wald ratio | 1 | -0.00671 | 0.016452 | 0.683351 | -0.03896 | 0.025535 | 0.993312 | 0.961792 | 1.025864 |
| ieu-a-1128 | ER- Breast | cg02405476 | Wald ratio | 1 | -0.02105 | 0.032119 | 0.51217 | -0.08401 | 0.0419 | 0.979167 | 0.919426 | 1.04279 |
| ieu-a-1128 | ER- Breast | cg13142374 | Wald ratio | 1 | 0.002926 | 0.047549 | 0.95093 | -0.09027 | 0.096123 | 1.00293 | 0.913684 | 1.100894 |
| ieu-a-1128 | ER- Breast | cg26728709 | Wald ratio | 1 | -0.04817 | 0.024896 | 0.053017 | -0.09697 | 0.000628 | 0.952973 | 0.907587 | 1.000628 |
| ieu-a-1128 | ER- Breast | cg00073460 | Wald ratio | 1 | 0.013889 | 0.045726 | 0.761327 | -0.07574 | 0.103513 | 1.013986 | 0.927062 | 1.10906 |
| ieu-a-1128 | ER- Breast | cg03234777 | Wald ratio | 1 | 0.005407 | 0.027037 | 0.841481 | -0.04759 | 0.0584 | 1.005422 | 0.953529 | 1.060139 |
| ieu-a-1128 | ER- Breast | cg02998240 | Wald ratio | 1 | 0.009185 | 0.014798 | 0.534804 | -0.01982 | 0.038189 | 1.009227 | 0.980376 | 1.038927 |
| ieu-a-1128 | ER- Breast | cg07090714 | Inverse vari | 2 | -0.00452 | 0.026546 | 0.864938 | -0.05654 | 0.047514 | 0.995495 | 0.945024 | 1.048661 |
| ieu-a-1128 | ER- Breast | cg21717508 | Wald ratio | 1 | -0.00908 | 0.057361 | 0.874194 | -0.12151 | 0.103346 | 0.990959 | 0.885582 | 1.108875 |
| ieu-a-1128 | ER- Breast | cg00981651 | Wald ratio | 1 | 0.01903 | 0.023327 | 0.414621 | -0.02669 | 0.064751 | 1.019212 | 0.973662 | 1.066894 |
| ieu-a-1128 | ER- Breast | cg11323506 | Wald ratio | 1 | 0.008233 | 0.03293 | 0.802587 | -0.05631 | 0.072776 | 1.008267 | 0.945245 | 1.07549 |
| ieu-a-1128 | ER- Breast | cg03144619 | Inverse vari | 2 | -0.02765 | 0.019363 | 0.153264 | -0.0656 | 0.010299 | 0.972727 | 0.936502 | 1.010353 |
| ieu-a-1128 | ER- Breast | cg04806562 | Wald ratio | 1 | 0.04632 | 0.060451 | 0.443537 | -0.07216 | 0.164805 | 1.047409 | 0.930377 | 1.179163 |
| ieu-a-1128 | ER- Breast | cg16936953 | Wald ratio | 1 | -0.09285 | 0.037956 | 0.014439 | -0.16724 | -0.01845 | 0.911333 | 0.845996 | 0.981717 |
| ieu-a-1128 | ER- Breast | cg25809905 | Wald ratio | 1 | 0.005958 | 0.025025 | 0.811807 | -0.04309 | 0.055007 | 1.005976 | 0.957825 | 1.056548 |
| ieu-a-1128 | ER- Breast | cg11229399 | Wald ratio | 1 | 0.005706 | 0.025432 | 0.822478 | -0.04414 | 0.055553 | 1.005722 | 0.956819 | 1.057125 |
| ieu-a-1128 | ER- Breast | cg20698421 | Inverse vari | 2 | -0.01757 | 0.021337 | 0.410176 | -0.05939 | 0.024247 | 0.982581 | 0.942337 | 1.024544 |
| ieu-a-1128 | ER- Breast | cg02508743 | Wald ratio | 1 | 0.037704 | 0.034055 | 0.268232 | -0.02904 | 0.104451 | 1.038424 | 0.971374 | 1.110102 |
| ieu-a-1128 | ER- Breast | cg07180646 | Wald ratio | 1 | 0.0332 | 0.039366 | 0.39902 | -0.04396 | 0.110358 | 1.033758 | 0.956995 | 1.116678 |
| ieu-a-1128 | ER- Breast | cg18503679 | Wald ratio | 1 | 0.027794 | 0.04096 | 0.497409 | -0.05249 | 0.108075 | 1.028184 | 0.948867 | 1.114131 |
| ieu-a-1128 | ER- Breast | cg18432895 | Wald ratio | 1 | 0.041894 | 0.022189 | 0.059018 | -0.0016 | 0.085385 | 1.042784 | 0.998405 | 1.089136 |
| ieu-a-1128 | ER- Breast | cg18584067 | Wald ratio | 1 | -0.09561 | 0.041021 | 0.019769 | -0.17601 | -0.01521 | 0.908821 | 0.838611 | 0.984908 |
| ieu-a-1128 | ER- Breast | cg09938479 | Wald ratio | 1 | -0.02983 | 0.033838 | 0.378084 | -0.09615 | 0.036497 | 0.970615 | 0.908329 | 1.037171 |
| ieu-a-1128 | ER- Breast | cg02660097 | Inverse vari | 2 | 0.030708 | 0.016279 | 0.059241 | -0.0012 | 0.062614 | 1.031184 | 0.998803 | 1.064616 |
| ieu-a-1128 | ER- Breast | cg05204104 | Wald ratio | 1 | -0.00516 | 0.055077 | 0.925308 | -0.11312 | 0.102788 | 0.99485 | 0.893048 | 1.108257 |
| ieu-a-1128 | ER- Breast | cg14316231 | Wald ratio | 1 | 0.008893 | 0.052468 | 0.86541 | -0.09394 | 0.11173 | 1.008933 | 0.910334 | 1.118211 |
| ieu-a-1128 | ER- Breast | cg26403843 | Wald ratio | 1 | 0.023909 | 0.056874 | 0.674206 | -0.08756 | 0.135382 | 1.024197 | 0.91616 | 1.144974 |
| ieu-a-1128 | ER- Breast | cg00024404 | Wald ratio | 1 | 0.000596 | 0.035192 | 0.986477 | -0.06838 | 0.069574 | 1.000597 | 0.933905 | 1.072051 |
| ieu-a-1128 | ER- Breast | cg20059377 | Wald ratio | 1 | -0.00762 | 0.016099 | 0.636012 | -0.03917 | 0.023935 | 0.992409 | 0.961583 | 1.024224 |
| ieu-a-1128 | ER- Breast | cg27025995 | Wald ratio | 1 | -0.03352 | 0.020463 | 0.10142 | -0.07363 | 0.006589 | 0.967037 | 0.929019 | 1.006611 |
| ieu-a-1128 | ER- Breast | cg20322193 | Wald ratio | 1 | 0.009011 | 0.039839 | 0.821053 | -0.06907 | 0.087095 | 1.009052 | 0.933259 | 1.091 |
| ieu-a-1128 | ER- Breast | cg00668559 | Wald ratio | 1 | -0.0148 | 0.01592 | 0.352462 | -0.046 | 0.0164 | 0.985307 | 0.955037 | 1.016535 |
| ieu-a-1128 | ER- Breast | cg26718213 | Wald ratio | 1 | -0.01341 | 0.029107 | 0.644892 | -0.07046 | 0.043635 | 0.986675 | 0.931962 | 1.044601 |
| ieu-a-1128 | ER- Breast | cg16274678 | Inverse vari | 2 | 0.044174 | 0.022146 | 0.046076 | 0.000768 | 0.08758 | 1.045165 | 1.000769 | 1.09153 |
| ieu-a-1128 | ER- Breast | cg14295611 | Wald ratio | 1 | 0.002371 | 0.047807 | 0.960452 | -0.09133 | 0.096073 | 1.002373 | 0.912715 | 1.100839 |
| ieu-a-1128 | ER- Breast | cg22650458 | Wald ratio | 1 | -0.03863 | 0.02247 | 0.085613 | -0.08267 | 0.005415 | 0.962111 | 0.920658 | 1.00543 |
| ieu-a-1128 | ER- Breast | cg06193043 | Wald ratio | 1 | 0.010952 | 0.015081 | 0.467722 | -0.01861 | 0.04051 | 1.011012 | 0.981565 | 1.041342 |

| ieu-a-1128 | ER- Breast | cg21913886 | Wald ratio | 1 | -0.00436 | 0.026759 | 0.87058 | -0.05681 | 0.048088 | 0.99565 | 0.944776 | 1.049263 |
| --- | --- | --- | --- | --- | --- | --- | --- | --- | --- | --- | --- | --- |
| ieu-a-1128 | ER- Breast | cg01565703 | Wald ratio | 1 | 0.040102 | 0.041231 | 0.330751 | -0.04071 | 0.120915 | 1.040917 | 0.960106 | 1.128529 |
| ieu-a-1128 | ER- Breast | cg16516405 | Wald ratio | 1 | 0.013576 | 0.024436 | 0.578515 | -0.03432 | 0.061471 | 1.013668 | 0.966263 | 1.0634 |
| ieu-a-1128 | ER- Breast | cg11660018 | Wald ratio | 1 | -0.01078 | 0.041702 | 0.796018 | -0.09252 | 0.070956 | 0.989278 | 0.911634 | 1.073534 |
| ieu-a-1128 | ER- Breast | cg08097581 | Wald ratio | 1 | -0.00609 | 0.02114 | 0.77344 | -0.04752 | 0.035349 | 0.993933 | 0.953591 | 1.035981 |
| ieu-a-1128 | ER- Breast | cg25347941 | Wald ratio | 1 | 0.008374 | 0.042179 | 0.842631 | -0.0743 | 0.091044 | 1.008409 | 0.928396 | 1.095318 |
| ieu-a-1128 | ER- Breast | cg04209460 | Wald ratio | 1 | 0.010261 | 0.019668 | 0.601852 | -0.02829 | 0.04881 | 1.010314 | 0.972109 | 1.050021 |
| ieu-a-1128 | ER- Breast | cg16554099 | Inverse vari | 2 | -0.05261 | 0.028526 | 0.065152 | -0.10852 | 0.003303 | 0.948752 | 0.897163 | 1.003308 |
| ieu-a-1128 | ER- Breast | cg19717773 | Inverse vari | 2 | 0.022532 | 0.025858 | 0.383545 | -0.02815 | 0.073214 | 1.022788 | 0.972243 | 1.07596 |
| ieu-a-1128 | ER- Breast | cg06065019 | Wald ratio | 1 | 0.032695 | 0.025749 | 0.204167 | -0.01777 | 0.083162 | 1.033235 | 0.982385 | 1.086717 |
| ieu-a-1128 | ER- Breast | cg11601297 | Wald ratio | 1 | -0.0002 | 0.035354 | 0.995441 | -0.06949 | 0.069091 | 0.999798 | 0.932865 | 1.071534 |
| ieu-a-1128 | ER- Breast | cg03222009 | Wald ratio | 1 | 0.018609 | 0.03379 | 0.581822 | -0.04762 | 0.084838 | 1.018783 | 0.953496 | 1.088541 |
| ieu-a-1128 | ER- Breast | cg11530213 | Wald ratio | 1 | -0.0083 | 0.020304 | 0.682763 | -0.04809 | 0.031497 | 0.991736 | 0.953045 | 1.031998 |
| ieu-a-1128 | ER- Breast | cg20853880 | Wald ratio | 1 | 0.014884 | 0.03473 | 0.668235 | -0.05319 | 0.082956 | 1.014996 | 0.948203 | 1.086494 |
| ieu-a-1128 | ER- Breast | cg18708252 | Wald ratio | 1 | 0.01387 | 0.019764 | 0.482832 | -0.02487 | 0.052607 | 1.013966 | 0.975438 | 1.054016 |
| ieu-a-1128 | ER- Breast | cg04019522 | Inverse vari | 2 | -0.02023 | 0.047483 | 0.670049 | -0.1133 | 0.072835 | 0.979972 | 0.892884 | 1.075553 |
| ieu-a-1128 | ER- Breast | cg19022697 | Wald ratio | 1 | -0.03448 | 0.029839 | 0.247924 | -0.09296 | 0.024008 | 0.966112 | 0.91123 | 1.024299 |
| ieu-a-1128 | ER- Breast | cg20379671 | Wald ratio | 1 | -0.04864 | 0.02323 | 0.036264 | -0.09418 | -0.00311 | 0.952521 | 0.910123 | 0.996893 |
| ieu-a-1128 | ER- Breast | cg00022866 | Wald ratio | 1 | -0.0165 | 0.012529 | 0.18795 | -0.04105 | 0.00806 | 0.983639 | 0.959779 | 1.008093 |
| ieu-a-1128 | ER- Breast | cg15029032 | Wald ratio | 1 | 0.013924 | 0.022694 | 0.539528 | -0.03056 | 0.058405 | 1.014021 | 0.969905 | 1.060144 |
| ieu-a-1128 | ER- Breast | cg09635954 | Inverse vari | 2 | 0.025187 | 0.021618 | 0.24399 | -0.01719 | 0.067559 | 1.025507 | 0.982962 | 1.069894 |
| ieu-a-1128 | ER- Breast | cg15059065 | Wald ratio | 1 | 0.074997 | 0.038838 | 0.053479 | -0.00112 | 0.151119 | 1.077881 | 0.998876 | 1.163135 |
| ieu-a-1128 | ER- Breast | cg05877788 | Wald ratio | 1 | -0.02715 | 0.018914 | 0.151149 | -0.06422 | 0.009921 | 0.973215 | 0.937797 | 1.00997 |
| ieu-a-1128 | ER- Breast | cg00579402 | Wald ratio | 1 | 0.017509 | 0.021924 | 0.424516 | -0.02546 | 0.060481 | 1.017663 | 0.974859 | 1.062348 |
| ieu-a-1128 | ER- Breast | cg02782510 | Wald ratio | 1 | -0.00616 | 0.053501 | 0.908281 | -0.11103 | 0.098698 | 0.993855 | 0.894916 | 1.103733 |
| ieu-a-1128 | ER- Breast | cg13443605 | Wald ratio | 1 | 0.023075 | 0.04555 | 0.61245 | -0.0662 | 0.112352 | 1.023343 | 0.935941 | 1.118907 |
| ieu-a-1128 | ER- Breast | cg17551891 | Inverse vari | 2 | -0.01347 | 0.05462 | 0.80521 | -0.12052 | 0.093585 | 0.98662 | 0.886455 | 1.098104 |
| ieu-a-1128 | ER- Breast | cg24426691 | Wald ratio | 1 | 0.060848 | 0.030424 | 0.0455 | 0.001217 | 0.120479 | 1.062737 | 1.001218 | 1.128037 |
| ieu-a-1128 | ER- Breast | cg19757176 | Wald ratio | 1 | 0.033228 | 0.022218 | 0.134764 | -0.01032 | 0.076775 | 1.033787 | 0.989735 | 1.0798 |
| ieu-a-1128 | ER- Breast | cg14242895 | Wald ratio | 1 | 0.020128 | 0.044831 | 0.653446 | -0.06774 | 0.107996 | 1.020332 | 0.934503 | 1.114044 |
| ieu-a-1128 | ER- Breast | cg03051880 | Wald ratio | 1 | 0.020047 | 0.033764 | 0.552679 | -0.04613 | 0.086225 | 1.02025 | 0.954918 | 1.090052 |
| ieu-a-1128 | ER- Breast | cg22041417 | Wald ratio | 1 | -0.01361 | 0.025408 | 0.592156 | -0.06341 | 0.036189 | 0.986481 | 0.938557 | 1.036852 |
| ieu-a-1128 | ER- Breast | cg14624381 | Wald ratio | 1 | 0.032243 | 0.029206 | 0.269593 | -0.025 | 0.089486 | 1.032768 | 0.97531 | 1.093612 |
| ieu-a-1128 | ER- Breast | cg13257421 | Inverse vari | 2 | 0.033745 | 0.049683 | 0.497003 | -0.06363 | 0.131124 | 1.034321 | 0.938349 | 1.14011 |
| ieu-a-1128 | ER- Breast | cg07827420 | Wald ratio | 1 | 0.019327 | 0.045613 | 0.671764 | -0.07007 | 0.108728 | 1.019515 | 0.932325 | 1.114859 |
| ieu-a-1128 | ER- Breast | cg17449254 | Wald ratio | 1 | 0.03125 | 0.038893 | 0.42169 | -0.04498 | 0.10748 | 1.031743 | 0.956017 | 1.113468 |
| ieu-a-1128 | ER- Breast | cg19030554 | Inverse vari | 2 | -0.02223 | 0.017935 | 0.215176 | -0.05738 | 0.012923 | 0.978016 | 0.944234 | 1.013007 |
| ieu-a-1128 | ER- Breast | cg18810691 | Wald ratio | 1 | 0.017598 | 0.019858 | 0.375521 | -0.02132 | 0.056519 | 1.017753 | 0.978902 | 1.058147 |

| ieu-a-1128 | ER- Breast | cg21307484 | Wald ratio | 1 | 0.019101 | 0.039083 | 0.625039 | -0.0575 | 0.095704 | 1.019284 | 0.94412 | 1.100433 |
| --- | --- | --- | --- | --- | --- | --- | --- | --- | --- | --- | --- | --- |
| ieu-a-1128 | ER- Breast | cg01328473 | Wald ratio | 1 | -0.00922 | 0.047744 | 0.846878 | -0.1028 | 0.08436 | 0.990823 | 0.902308 | 1.08802 |
| ieu-a-1128 | ER- Breast | cg17533477 | Wald ratio | 1 | 0.052445 | 0.043977 | 0.233047 | -0.03375 | 0.13864 | 1.053844 | 0.966813 | 1.14871 |
| ieu-a-1128 | ER- Breast | cg07520810 | Wald ratio | 1 | 0.086087 | 0.047328 | 0.068917 | -0.00667 | 0.178849 | 1.089901 | 0.993347 | 1.195841 |
| ieu-a-1128 | ER- Breast | cg22858500 | Wald ratio | 1 | -0.0243 | 0.031603 | 0.441921 | -0.08624 | 0.037641 | 0.975992 | 0.917371 | 1.038358 |
| ieu-a-1128 | ER- Breast | cg02871659 | Wald ratio | 1 | -0.02457 | 0.020659 | 0.234365 | -0.06506 | 0.015924 | 0.975732 | 0.937013 | 1.016052 |
| ieu-a-1128 | ER- Breast | cg05720226 | Wald ratio | 1 | -0.00815 | 0.040228 | 0.839367 | -0.087 | 0.070693 | 0.991879 | 0.916675 | 1.073252 |
| ieu-a-1128 | ER- Breast | cg02672759 | Wald ratio | 1 | 0.060759 | 0.049186 | 0.216721 | -0.03565 | 0.157165 | 1.062643 | 0.964982 | 1.170188 |
| ieu-a-1128 | ER- Breast | cg22678092 | Wald ratio | 1 | -0.01387 | 0.022412 | 0.536111 | -0.05779 | 0.030061 | 0.986229 | 0.943844 | 1.030518 |
| ieu-a-1128 | ER- Breast | cg10403394 | Inverse vari | 2 | -0.01042 | 0.038544 | 0.786988 | -0.08596 | 0.065131 | 0.989639 | 0.917629 | 1.067299 |
| ieu-a-1128 | ER- Breast | cg00689360 | Inverse vari | 2 | -0.03343 | 0.025325 | 0.18684 | -0.08307 | 0.016209 | 0.967123 | 0.92029 | 1.016341 |
| ieu-a-1128 | ER- Breast | cg19696491 | Wald ratio | 1 | -0.02019 | 0.030553 | 0.508696 | -0.08007 | 0.039692 | 0.980011 | 0.923048 | 1.04049 |
| ieu-a-1128 | ER- Breast | cg22132788 | Wald ratio | 1 | 0.018842 | 0.036075 | 0.601466 | -0.05187 | 0.08955 | 1.019021 | 0.949456 | 1.093682 |
| ieu-a-1128 | ER- Breast | cg12289251 | Wald ratio | 1 | -0.02135 | 0.035887 | 0.551917 | -0.09169 | 0.04899 | 0.978877 | 0.91239 | 1.05021 |
| ieu-a-1128 | ER- Breast | cg16037981 | Wald ratio | 1 | -0.0173 | 0.036733 | 0.637588 | -0.0893 | 0.054693 | 0.982845 | 0.91457 | 1.056217 |
| ieu-a-1128 | ER- Breast | cg16983588 | Wald ratio | 1 | -0.01844 | 0.042906 | 0.667323 | -0.10254 | 0.065653 | 0.981727 | 0.902545 | 1.067856 |
| ieu-a-1128 | ER- Breast | cg00580497 | Wald ratio | 1 | 0.016663 | 0.024608 | 0.498301 | -0.03157 | 0.064894 | 1.016803 | 0.968926 | 1.067046 |
| ieu-a-1128 | ER- Breast | cg00893603 | Wald ratio | 1 | -0.00372 | 0.021058 | 0.859924 | -0.04499 | 0.037558 | 0.996291 | 0.956007 | 1.038272 |
| ieu-a-1128 | ER- Breast | cg06670463 | Wald ratio | 1 | 0.014053 | 0.035133 | 0.689157 | -0.05481 | 0.082914 | 1.014152 | 0.946668 | 1.086448 |
| ieu-a-1128 | ER- Breast | cg19406367 | Wald ratio | 1 | -0.01488 | 0.03101 | 0.631227 | -0.07566 | 0.045894 | 0.985226 | 0.927128 | 1.046964 |
| ieu-a-1128 | ER- Breast | cg19872095 | Wald ratio | 1 | 0.026871 | 0.073358 | 0.714141 | -0.11691 | 0.170652 | 1.027235 | 0.889665 | 1.186077 |
| ieu-a-1128 | ER- Breast | cg22586569 | Wald ratio | 1 | 0.019961 | 0.023062 | 0.386739 | -0.02524 | 0.065163 | 1.020162 | 0.975076 | 1.067333 |
| ieu-a-1128 | ER- Breast | cg20408402 | Wald ratio | 1 | -0.00381 | 0.034276 | 0.911528 | -0.07099 | 0.063373 | 0.996199 | 0.931471 | 1.065424 |
| ieu-a-1128 | ER- Breast | cg27409015 | Wald ratio | 1 | 0.016402 | 0.035273 | 0.641931 | -0.05273 | 0.085538 | 1.016537 | 0.948633 | 1.089303 |
| ieu-a-1128 | ER- Breast | cg26253500 | Wald ratio | 1 | -0.01934 | 0.037776 | 0.608651 | -0.09338 | 0.054699 | 0.980845 | 0.910846 | 1.056223 |
| ieu-a-1128 | ER- Breast | cg26585644 | Wald ratio | 1 | 0.047089 | 0.042432 | 0.267104 | -0.03608 | 0.130256 | 1.048216 | 0.964565 | 1.13912 |
| ieu-a-1128 | ER- Breast | cg00099441 | Wald ratio | 1 | -0.02606 | 0.034565 | 0.450942 | -0.0938 | 0.041691 | 0.97428 | 0.91046 | 1.042572 |
| ieu-a-1128 | ER- Breast | cg24049493 | Wald ratio | 1 | -0.00207 | 0.029747 | 0.94454 | -0.06037 | 0.056234 | 0.997933 | 0.941414 | 1.057845 |
| ieu-a-1128 | ER- Breast | cg14459011 | Wald ratio | 1 | 0.050694 | 0.050427 | 0.314757 | -0.04814 | 0.14953 | 1.052001 | 0.952997 | 1.161289 |
| ieu-a-1128 | ER- Breast | cg23762517 | Wald ratio | 1 | -0.00846 | 0.015691 | 0.589797 | -0.03921 | 0.022295 | 0.991576 | 0.961545 | 1.022545 |
| ieu-a-1128 | ER- Breast | cg07602659 | Inverse vari | 2 | 0.040173 | 0.028608 | 0.160243 | -0.0159 | 0.096245 | 1.040991 | 0.984227 | 1.101028 |
| ieu-a-1128 | ER- Breast | cg27526649 | Wald ratio | 1 | -0.03444 | 0.020869 | 0.09886 | -0.07534 | 0.006461 | 0.966145 | 0.927424 | 1.006482 |
| ieu-a-1128 | ER- Breast | cg18151030 | Wald ratio | 1 | 0.016133 | 0.034748 | 0.642443 | -0.05197 | 0.084239 | 1.016264 | 0.949354 | 1.087889 |
| ieu-a-1128 | ER- Breast | cg05122453 | Wald ratio | 1 | 0.00412 | 0.038352 | 0.914441 | -0.07105 | 0.07929 | 1.004129 | 0.931416 | 1.082518 |
| ieu-a-1128 | ER- Breast | cg21201401 | Wald ratio | 1 | -0.01807 | 0.057143 | 0.751869 | -0.13007 | 0.093933 | 0.982095 | 0.878036 | 1.098486 |
| ieu-a-1128 | ER- Breast | cg25839482 | Wald ratio | 1 | -0.08658 | 0.023435 | 0.00022 | -0.13252 | -0.04065 | 0.917059 | 0.87589 | 0.960164 |
| ieu-a-1128 | ER- Breast | cg23884241 | Wald ratio | 1 | -0.00497 | 0.023849 | 0.834969 | -0.05171 | 0.041775 | 0.995044 | 0.949602 | 1.04266 |
| ieu-a-1128 | ER- Breast | cg12275060 | Wald ratio | 1 | 0.014871 | 0.033109 | 0.653322 | -0.05002 | 0.079764 | 1.014982 | 0.951208 | 1.083032 |

| ieu-a-1128 | ER- Breast | cg03373393 | Inverse vari | 2 | -0.04443 | 0.0314 | 0.1571 | -0.10597 | 0.017116 | 0.956545 | 0.899452 | 1.017263 |
| --- | --- | --- | --- | --- | --- | --- | --- | --- | --- | --- | --- | --- |
| ieu-a-1128 | ER- Breast | cg18033416 | Wald ratio | 1 | -0.06995 | 0.0488 | 0.151763 | -0.1656 | 0.025702 | 0.932443 | 0.847389 | 1.026035 |
| ieu-a-1128 | ER- Breast | cg06861736 | Wald ratio | 1 | -0.03666 | 0.051092 | 0.47303 | -0.1368 | 0.063479 | 0.964002 | 0.872143 | 1.065537 |
| ieu-a-1128 | ER- Breast | cg22563815 | Wald ratio | 1 | -0.02149 | 0.032523 | 0.508696 | -0.08524 | 0.042251 | 0.978736 | 0.918294 | 1.043156 |
| ieu-a-1128 | ER- Breast | cg26146569 | Wald ratio | 1 | 0.009681 | 0.041142 | 0.81398 | -0.07096 | 0.090319 | 1.009728 | 0.931501 | 1.094524 |
| ieu-a-1128 | ER- Breast | cg25625514 | Wald ratio | 1 | 0.012029 | 0.01953 | 0.537933 | -0.02625 | 0.050309 | 1.012102 | 0.974092 | 1.051595 |
| ieu-a-1128 | ER- Breast | cg24497361 | Wald ratio | 1 | -0.02989 | 0.039636 | 0.45079 | -0.10758 | 0.047797 | 0.970553 | 0.898008 | 1.048958 |
| ieu-a-1128 | ER- Breast | cg00689225 | Wald ratio | 1 | 0.021476 | 0.033819 | 0.525405 | -0.04481 | 0.087761 | 1.021708 | 0.95618 | 1.091727 |
| ieu-a-1128 | ER- Breast | cg26574777 | Wald ratio | 1 | -0.0008 | 0.030198 | 0.97882 | -0.05999 | 0.058386 | 0.999199 | 0.941775 | 1.060124 |
| ieu-a-1128 | ER- Breast | cg23161492 | Wald ratio | 1 | -0.0396 | 0.05653 | 0.483633 | -0.1504 | 0.071201 | 0.961176 | 0.860368 | 1.073797 |
| ieu-a-1128 | ER- Breast | cg17884674 | Wald ratio | 1 | -0.01577 | 0.028726 | 0.583093 | -0.07207 | 0.040536 | 0.984357 | 0.930467 | 1.041368 |
| ieu-a-1128 | ER- Breast | cg14841514 | Wald ratio | 1 | 0.005471 | 0.044158 | 0.901399 | -0.08108 | 0.09202 | 1.005486 | 0.922121 | 1.096387 |
| ieu-a-1128 | ER- Breast | cg08384239 | Wald ratio | 1 | -0.00853 | 0.022681 | 0.706775 | -0.05299 | 0.035922 | 0.991504 | 0.948393 | 1.036575 |
| ieu-a-1128 | ER- Breast | cg26937798 | Wald ratio | 1 | 0.058911 | 0.042979 | 0.170472 | -0.02533 | 0.143149 | 1.060681 | 0.97499 | 1.153902 |
| ieu-a-1128 | ER- Breast | cg01447828 | Wald ratio | 1 | 0.014474 | 0.026316 | 0.582319 | -0.03711 | 0.066053 | 1.014579 | 0.963575 | 1.068283 |
| ieu-a-1128 | ER- Breast | cg04816394 | Wald ratio | 1 | -0.00437 | 0.032866 | 0.894298 | -0.06878 | 0.060051 | 0.995643 | 0.933528 | 1.06189 |
| ieu-a-1128 | ER- Breast | cg10750959 | Wald ratio | 1 | 0.04584 | 0.047963 | 0.339197 | -0.04817 | 0.139847 | 1.046907 | 0.952975 | 1.150098 |
| ieu-a-1128 | ER- Breast | cg03480935 | Wald ratio | 1 | 0.048676 | 0.032362 | 0.132547 | -0.01475 | 0.112105 | 1.04988 | 0.985356 | 1.11863 |
| ieu-a-1128 | ER- Breast | cg26599989 | Wald ratio | 1 | 0.017729 | 0.035458 | 0.617075 | -0.05177 | 0.087227 | 1.017887 | 0.949548 | 1.091144 |
| ieu-a-1128 | ER- Breast | cg03519967 | Wald ratio | 1 | 0.017327 | 0.041151 | 0.673717 | -0.06333 | 0.097983 | 1.017478 | 0.938634 | 1.102944 |
| ieu-a-1128 | ER- Breast | cg18585107 | Inverse vari | 2 | 0.021088 | 0.060745 | 0.728474 | -0.09797 | 0.140147 | 1.021312 | 0.906675 | 1.150443 |
| ieu-a-1128 | ER- Breast | cg15950273 | Wald ratio | 1 | 0.07 | 0.031456 | 0.026061 | 0.008346 | 0.131654 | 1.072508 | 1.008381 | 1.140714 |
| ieu-a-1128 | ER- Breast | cg26161820 | Wald ratio | 1 | -0.0221 | 0.048335 | 0.647568 | -0.11683 | 0.072641 | 0.978146 | 0.889734 | 1.075344 |
| ieu-a-1128 | ER- Breast | cg07277038 | Inverse vari | 3 | -0.05991 | 0.051977 | 0.249095 | -0.16178 | 0.041968 | 0.941853 | 0.850628 | 1.042862 |
| ieu-a-1128 | ER- Breast | cg26076054 | Inverse vari | 2 | 0.066156 | 0.034309 | 0.053825 | -0.00109 | 0.133401 | 1.068393 | 0.998911 | 1.142708 |
| ieu-a-1128 | ER- Breast | cg15787744 | Wald ratio | 1 | -0.02719 | 0.029238 | 0.352462 | -0.08449 | 0.030121 | 0.97318 | 0.918978 | 1.030579 |
| ieu-a-1128 | ER- Breast | cg01643605 | Wald ratio | 1 | 0.069898 | 0.042501 | 0.100047 | -0.0134 | 0.1532 | 1.072399 | 0.986686 | 1.165558 |
| ieu-a-1128 | ER- Breast | cg12421513 | Wald ratio | 1 | 0.063035 | 0.034884 | 0.07076 | -0.00534 | 0.131408 | 1.065065 | 0.994678 | 1.140433 |
| ieu-a-1128 | ER- Breast | cg14330293 | Wald ratio | 1 | 0.026854 | 0.054135 | 0.61985 | -0.07925 | 0.132958 | 1.027218 | 0.923809 | 1.142202 |
| ieu-a-1128 | ER- Breast | cg21869609 | Wald ratio | 1 | 0.00207 | 0.017253 | 0.904483 | -0.03175 | 0.035887 | 1.002073 | 0.968753 | 1.036539 |
| ieu-a-1128 | ER- Breast | cg11461808 | Wald ratio | 1 | 0.018937 | 0.034765 | 0.585949 | -0.0492 | 0.087077 | 1.019118 | 0.951988 | 1.090981 |
| ieu-a-1128 | ER- Breast | cg01561259 | Wald ratio | 1 | 0.008375 | 0.049832 | 0.86653 | -0.0893 | 0.106047 | 1.00841 | 0.914574 | 1.111874 |
| ieu-a-1128 | ER- Breast | cg10453071 | Inverse vari | 2 | 0.004233 | 0.033916 | 0.900671 | -0.06224 | 0.07071 | 1.004242 | 0.939654 | 1.073269 |
| ieu-a-1128 | ER- Breast | cg23222488 | Wald ratio | 1 | -0.05376 | 0.058799 | 0.360567 | -0.169 | 0.061487 | 0.947661 | 0.844505 | 1.063416 |
| ieu-a-1128 | ER- Breast | cg23813257 | Wald ratio | 1 | -0.00977 | 0.049211 | 0.842675 | -0.10622 | 0.086687 | 0.99028 | 0.899226 | 1.090555 |
| ieu-a-1128 | ER- Breast | cg23940612 | Wald ratio | 1 | 0.00465 | 0.021577 | 0.829364 | -0.03764 | 0.046942 | 1.004661 | 0.963058 | 1.048061 |
| ieu-a-1128 | ER- Breast | cg12504098 | Inverse vari | 2 | 0.067093 | 0.03396 | 0.048192 | 0.000532 | 0.133654 | 1.069395 | 1.000532 | 1.142997 |
| ieu-a-1128 | ER- Breast | cg02373104 | Wald ratio | 1 | 0.002926 | 0.023991 | 0.902938 | -0.0441 | 0.049947 | 1.00293 | 0.956862 | 1.051216 |

| ieu-a-1128 | ER- Breast | cg18946533 | Wald ratio | 1 | -0.07811 | 0.050892 | 0.124847 | -0.17785 | 0.021642 | 0.924867 | 0.837065 | 1.021878 |
| --- | --- | --- | --- | --- | --- | --- | --- | --- | --- | --- | --- | --- |
| ieu-a-1128 | ER- Breast | cg02068690 | Wald ratio | 1 | 0.1 | 0.061979 | 0.106648 | -0.02148 | 0.221479 | 1.105171 | 0.97875 | 1.247921 |
| ieu-a-1128 | ER- Breast | cg18369034 | Inverse vari | 2 | 0.05751 | 0.02909 | 0.048046 | 0.000493 | 0.114527 | 1.059196 | 1.000493 | 1.121343 |
| ieu-a-1128 | ER- Breast | cg04144533 | Wald ratio | 1 | 0.026563 | 0.028395 | 0.349539 | -0.02909 | 0.082217 | 1.026919 | 0.971328 | 1.085691 |
| ieu-a-1128 | ER- Breast | cg25649826 | Wald ratio | 1 | -0.01946 | 0.064298 | 0.762171 | -0.14548 | 0.106565 | 0.98073 | 0.864605 | 1.11245 |
| ieu-a-1128 | ER- Breast | cg07839313 | Wald ratio | 1 | -0.05683 | 0.042844 | 0.184711 | -0.1408 | 0.027147 | 0.944756 | 0.86866 | 1.027519 |
| ieu-a-1128 | ER- Breast | cg14357089 | Wald ratio | 1 | -0.02327 | 0.03117 | 0.455409 | -0.08436 | 0.037827 | 0.977002 | 0.919101 | 1.038552 |
| ieu-a-1128 | ER- Breast | cg09701700 | Wald ratio | 1 | -0.01629 | 0.023155 | 0.481813 | -0.06167 | 0.029097 | 0.983845 | 0.940191 | 1.029525 |
| ieu-a-1128 | ER- Breast | cg15331301 | Wald ratio | 1 | 0.034362 | 0.045582 | 0.450942 | -0.05498 | 0.123703 | 1.034959 | 0.946505 | 1.131679 |
| ieu-a-1128 | ER- Breast | cg02462416 | Wald ratio | 1 | -0.01429 | 0.019971 | 0.474406 | -0.05343 | 0.024857 | 0.985816 | 0.947974 | 1.025169 |
| ieu-a-1128 | ER- Breast | cg05379350 | Wald ratio | 1 | -0.09355 | 0.041345 | 0.023653 | -0.17459 | -0.01252 | 0.91069 | 0.839801 | 0.987562 |
| ieu-a-1128 | ER- Breast | cg09479241 | Inverse vari | 2 | 0.030916 | 0.025878 | 0.232218 | -0.01981 | 0.081637 | 1.031399 | 0.980389 | 1.085062 |
| ieu-a-1128 | ER- Breast | cg22905866 | Inverse vari | 2 | 0.009252 | 0.024155 | 0.701693 | -0.03809 | 0.056597 | 1.009295 | 0.962624 | 1.058229 |
| ieu-a-1128 | ER- Breast | cg15951188 | Wald ratio | 1 | -0.04865 | 0.026015 | 0.061495 | -0.09964 | 0.002343 | 0.952518 | 0.905167 | 1.002346 |
| ieu-a-1128 | ER- Breast | cg03785755 | Wald ratio | 1 | 0.011759 | 0.038877 | 0.762294 | -0.06444 | 0.087958 | 1.011828 | 0.937593 | 1.091942 |
| ieu-a-1128 | ER- Breast | cg00177243 | Wald ratio | 1 | 0.012715 | 0.021373 | 0.551917 | -0.02918 | 0.054605 | 1.012796 | 0.971245 | 1.056124 |
| ieu-a-1128 | ER- Breast | cg20912205 | Wald ratio | 1 | -0.07229 | 0.034997 | 0.038869 | -0.14088 | -0.00369 | 0.930262 | 0.86859 | 0.996312 |
| ieu-a-1128 | ER- Breast | cg03333699 | Wald ratio | 1 | 0.006031 | 0.036473 | 0.868665 | -0.06546 | 0.077519 | 1.006049 | 0.93664 | 1.080602 |
| ieu-a-1128 | ER- Breast | cg01435643 | Wald ratio | 1 | -0.02388 | 0.033128 | 0.470952 | -0.08881 | 0.041048 | 0.9764 | 0.915016 | 1.041902 |
| ieu-a-1128 | ER- Breast | cg06283478 | Wald ratio | 1 | -0.04067 | 0.046723 | 0.384025 | -0.13225 | 0.050904 | 0.960144 | 0.876123 | 1.052222 |
| ieu-a-1128 | ER- Breast | cg07506795 | Wald ratio | 1 | 0.004428 | 0.026407 | 0.866847 | -0.04733 | 0.056186 | 1.004437 | 0.953772 | 1.057794 |
| ieu-a-1128 | ER- Breast | cg20469837 | Wald ratio | 1 | 0.020509 | 0.035708 | 0.565725 | -0.04948 | 0.090496 | 1.020721 | 0.951726 | 1.094717 |
| ieu-a-1128 | ER- Breast | cg03188382 | Wald ratio | 1 | -0.06625 | 0.040118 | 0.098662 | -0.14488 | 0.012381 | 0.935897 | 0.865126 | 1.012458 |
| ieu-a-1128 | ER- Breast | cg10180092 | Inverse vari | 2 | -0.02452 | 0.018508 | 0.185152 | -0.0608 | 0.011752 | 0.975774 | 0.94101 | 1.011821 |
| ieu-a-1128 | ER- Breast | cg07450086 | Wald ratio | 1 | -0.02686 | 0.02402 | 0.263387 | -0.07394 | 0.020215 | 0.973493 | 0.928723 | 1.020421 |
| ieu-a-1128 | ER- Breast | cg03991871 | Wald ratio | 1 | 0.041888 | 0.032468 | 0.197008 | -0.02175 | 0.105526 | 1.042778 | 0.978485 | 1.111295 |
| ieu-a-1128 | ER- Breast | cg08787968 | Wald ratio | 1 | -0.01893 | 0.035899 | 0.597918 | -0.08929 | 0.051429 | 0.981245 | 0.914576 | 1.052774 |
| ieu-a-1128 | ER- Breast | cg09206294 | Wald ratio | 1 | 0.007261 | 0.048003 | 0.87977 | -0.08683 | 0.101347 | 1.007287 | 0.916837 | 1.106661 |
| ieu-a-1128 | ER- Breast | cg02704502 | Wald ratio | 1 | -0.01561 | 0.021815 | 0.474406 | -0.05836 | 0.027153 | 0.984516 | 0.943307 | 1.027525 |
| ieu-a-1128 | ER- Breast | cg26351966 | Inverse vari | 2 | 0.030501 | 0.02311 | 0.186888 | -0.01479 | 0.075797 | 1.030971 | 0.985315 | 1.078743 |
| ieu-a-1128 | ER- Breast | cg24490227 | Wald ratio | 1 | 0.064183 | 0.062229 | 0.302358 | -0.05779 | 0.186152 | 1.066287 | 0.943851 | 1.204605 |
| ieu-a-1128 | ER- Breast | cg11130692 | Wald ratio | 1 | -0.00886 | 0.055977 | 0.874305 | -0.11857 | 0.10086 | 0.991184 | 0.888189 | 1.106122 |
| ieu-a-1128 | ER- Breast | cg07941108 | Wald ratio | 1 | -0.03262 | 0.046211 | 0.480261 | -0.12319 | 0.057954 | 0.967907 | 0.884092 | 1.059667 |
| ieu-a-1128 | ER- Breast | cg10959726 | Wald ratio | 1 | -0.01016 | 0.046302 | 0.826251 | -0.10091 | 0.080587 | 0.989888 | 0.90401 | 1.083923 |
| ieu-a-1128 | ER- Breast | cg21201657 | Wald ratio | 1 | -0.02041 | 0.036971 | 0.580948 | -0.09287 | 0.052056 | 0.979799 | 0.91131 | 1.053434 |
| ieu-a-1128 | ER- Breast | cg08548559 | Wald ratio | 1 | 0.047567 | 0.022707 | 0.036186 | 0.003061 | 0.092072 | 1.048716 | 1.003066 | 1.096443 |
| ieu-a-1128 | ER- Breast | cg17009069 | Wald ratio | 1 | 0.024466 | 0.043883 | 0.577171 | -0.06155 | 0.110478 | 1.024768 | 0.94031 | 1.116811 |
| ieu-a-1128 | ER- Breast | cg14391923 | Inverse vari | 2 | -0.02071 | 0.019655 | 0.291931 | -0.05924 | 0.01781 | 0.979498 | 0.942481 | 1.017969 |

| ieu-a-1128 | ER- Breast | cg07421287 | Wald ratio | 1 | 0.037477 | 0.036563 | 0.305363 | -0.03419 | 0.109141 | 1.038188 | 0.966391 | 1.115319 |
| --- | --- | --- | --- | --- | --- | --- | --- | --- | --- | --- | --- | --- |
| ieu-a-1128 | ER- Breast | cg18446336 | Wald ratio | 1 | 0.041242 | 0.030279 | 0.173176 | -0.0181 | 0.10059 | 1.042105 | 0.982058 | 1.105823 |
| ieu-a-1128 | ER- Breast | cg03609435 | Wald ratio | 1 | 0.038348 | 0.048304 | 0.427258 | -0.05633 | 0.133024 | 1.039093 | 0.94523 | 1.142277 |
| ieu-a-1128 | ER- Breast | cg10255761 | Wald ratio | 1 | -0.04333 | 0.040258 | 0.281777 | -0.12224 | 0.035575 | 0.957594 | 0.884938 | 1.036215 |
| ieu-a-1128 | ER- Breast | cg03789791 | Wald ratio | 1 | -0.01578 | 0.03278 | 0.630339 | -0.08002 | 0.048474 | 0.984348 | 0.923094 | 1.049668 |
| ieu-a-1128 | ER- Breast | cg20124610 | Wald ratio | 1 | 0.016273 | 0.039914 | 0.6835 | -0.06196 | 0.094504 | 1.016406 | 0.939922 | 1.099114 |
| ieu-a-1128 | ER- Breast | cg17580614 | Wald ratio | 1 | 0.011864 | 0.021109 | 0.574086 | -0.02951 | 0.053239 | 1.011935 | 0.970921 | 1.054682 |
| ieu-a-1128 | ER- Breast | cg25284397 | Wald ratio | 1 | -0.02259 | 0.043669 | 0.604895 | -0.10818 | 0.062998 | 0.97766 | 0.897462 | 1.065025 |
| ieu-a-1128 | ER- Breast | cg02532700 | Wald ratio | 1 | -0.00553 | 0.026654 | 0.835692 | -0.05777 | 0.046713 | 0.994487 | 0.943868 | 1.047821 |
| ieu-a-1128 | ER- Breast | cg10590964 | Wald ratio | 1 | -0.00837 | 0.032251 | 0.795217 | -0.07158 | 0.054841 | 0.991665 | 0.93092 | 1.056373 |
| ieu-a-1128 | ER- Breast | cg10717312 | Inverse vari | 2 | -0.01182 | 0.022393 | 0.597625 | -0.05571 | 0.032071 | 0.98825 | 0.945813 | 1.032591 |
| ieu-a-1128 | ER- Breast | cg18617091 | Wald ratio | 1 | -0.0168 | 0.025034 | 0.502131 | -0.06587 | 0.032265 | 0.983339 | 0.936255 | 1.032791 |
| ieu-a-1128 | ER- Breast | cg09069072 | Wald ratio | 1 | 0.02042 | 0.017584 | 0.245524 | -0.01404 | 0.054884 | 1.02063 | 0.986054 | 1.056418 |
| ieu-a-1128 | ER- Breast | cg26132737 | Wald ratio | 1 | 0.003182 | 0.05118 | 0.950423 | -0.09713 | 0.103495 | 1.003187 | 0.907437 | 1.10904 |
| ieu-a-1128 | ER- Breast | cg27618939 | Wald ratio | 1 | 0.119665 | 0.04295 | 0.005334 | 0.035484 | 0.203847 | 1.127119 | 1.036121 | 1.22611 |
| ieu-a-1128 | ER- Breast | cg11827514 | Wald ratio | 1 | 0.001131 | 0.020355 | 0.955696 | -0.03877 | 0.041027 | 1.001131 | 0.961976 | 1.041881 |
| ieu-a-1128 | ER- Breast | cg01062937 | Wald ratio | 1 | -0.11311 | 0.073086 | 0.121714 | -0.25636 | 0.030139 | 0.893053 | 0.773865 | 1.030598 |
| ieu-a-1128 | ER- Breast | cg15578140 | Wald ratio | 1 | -0.04035 | 0.044754 | 0.367248 | -0.12807 | 0.047366 | 0.960451 | 0.879791 | 1.048506 |
| ieu-a-1128 | ER- Breast | cg04396998 | Wald ratio | 1 | 0.012091 | 0.024771 | 0.625482 | -0.03646 | 0.060643 | 1.012164 | 0.964195 | 1.062519 |
| ieu-a-1128 | ER- Breast | cg15089077 | Wald ratio | 1 | -0.02548 | 0.022057 | 0.247924 | -0.06872 | 0.017747 | 0.974838 | 0.933592 | 1.017905 |
| ieu-a-1128 | ER- Breast | cg18824446 | Wald ratio | 1 | -0.05733 | 0.018636 | 0.002096 | -0.09385 | -0.0208 | 0.944284 | 0.910416 | 0.979413 |
| ieu-a-1128 | ER- Breast | cg23395310 | Inverse vari | 2 | 0.007504 | 0.035836 | 0.834145 | -0.06274 | 0.077743 | 1.007532 | 0.939192 | 1.080845 |
| ieu-a-1128 | ER- Breast | cg16416158 | Wald ratio | 1 | 0.032929 | 0.025226 | 0.191776 | -0.01651 | 0.082372 | 1.033477 | 0.983621 | 1.08586 |
| ieu-a-1128 | ER- Breast | cg18262201 | Wald ratio | 1 | -0.03187 | 0.034065 | 0.349442 | -0.09864 | 0.034893 | 0.968629 | 0.906069 | 1.035509 |
| ieu-a-1128 | ER- Breast | cg13452162 | Wald ratio | 1 | 0.088412 | 0.037259 | 0.01765 | 0.015384 | 0.16144 | 1.092438 | 1.015503 | 1.175202 |
| ieu-a-1128 | ER- Breast | cg17127702 | Inverse vari | 2 | -0.00897 | 0.015963 | 0.574258 | -0.04026 | 0.02232 | 0.991072 | 0.960544 | 1.022571 |
| ieu-a-1128 | ER- Breast | cg20533899 | Wald ratio | 1 | -0.01305 | 0.040117 | 0.745027 | -0.09168 | 0.065584 | 0.987038 | 0.9124 | 1.067782 |
| ieu-a-1128 | ER- Breast | cg11212234 | Wald ratio | 1 | 0.001443 | 0.020208 | 0.943057 | -0.03816 | 0.041051 | 1.001444 | 0.962555 | 1.041905 |
| ieu-a-1128 | ER- Breast | cg10381071 | Wald ratio | 1 | -0.00233 | 0.024946 | 0.925486 | -0.05123 | 0.046561 | 0.99767 | 0.950062 | 1.047662 |
| ieu-a-1128 | ER- Breast | cg15059804 | Wald ratio | 1 | 0.018155 | 0.020697 | 0.380382 | -0.02241 | 0.058722 | 1.018321 | 0.977838 | 1.06048 |
| ieu-a-1128 | ER- Breast | cg08035323 | Wald ratio | 1 | 0.041683 | 0.04863 | 0.391366 | -0.05363 | 0.136997 | 1.042564 | 0.947781 | 1.146825 |
| ieu-a-1128 | ER- Breast | cg09658497 | Wald ratio | 1 | 0.036405 | 0.023858 | 0.127044 | -0.01036 | 0.083167 | 1.037076 | 0.989696 | 1.086724 |
| ieu-a-1128 | ER- Breast | cg17098103 | Wald ratio | 1 | -0.02326 | 0.032898 | 0.479631 | -0.08774 | 0.041225 | 0.977013 | 0.916002 | 1.042087 |
| ieu-a-1128 | ER- Breast | cg19802390 | Wald ratio | 1 | 0.029141 | 0.026794 | 0.276776 | -0.02338 | 0.081659 | 1.02957 | 0.976895 | 1.085085 |
| ieu-a-1128 | ER- Breast | cg16786458 | Inverse vari | 2 | 0.040647 | 0.02549 | 0.110797 | -0.00931 | 0.090606 | 1.041484 | 0.99073 | 1.094838 |
| ieu-a-1128 | ER- Breast | cg14656441 | Wald ratio | 1 | 0.01675 | 0.03075 | 0.585949 | -0.04352 | 0.07702 | 1.016891 | 0.957413 | 1.080064 |
| ieu-a-1128 | ER- Breast | cg26963277 | Wald ratio | 1 | -0.03871 | 0.041708 | 0.353408 | -0.12045 | 0.043043 | 0.962034 | 0.886518 | 1.043983 |
| ieu-a-1128 | ER- Breast | cg01940297 | Wald ratio | 1 | 0.008575 | 0.023683 | 0.7173 | -0.03784 | 0.054994 | 1.008612 | 0.962863 | 1.056534 |

| ieu-a-1128 | ER- Breast | cg21664281 | Wald ratio | 1 | 0.009645 | 0.018548 | 0.603064 | -0.02671 | 0.046 | 1.009692 | 0.973644 | 1.047074 |
| --- | --- | --- | --- | --- | --- | --- | --- | --- | --- | --- | --- | --- |
| ieu-a-1128 | ER- Breast | cg13782866 | Wald ratio | 1 | -0.01594 | 0.021743 | 0.463355 | -0.05856 | 0.026671 | 0.984182 | 0.94312 | 1.02703 |
| ieu-a-1128 | ER- Breast | cg10858677 | Wald ratio | 1 | 0.023057 | 0.037575 | 0.539456 | -0.05059 | 0.096704 | 1.023325 | 0.950669 | 1.101534 |
| ieu-a-1128 | ER- Breast | cg26908328 | Wald ratio | 1 | 0.000612 | 0.036108 | 0.986477 | -0.07016 | 0.071383 | 1.000612 | 0.932245 | 1.073993 |
| ieu-a-1128 | ER- Breast | cg23681440 | Wald ratio | 1 | -0.00632 | 0.042629 | 0.882226 | -0.08987 | 0.077237 | 0.993705 | 0.914052 | 1.080298 |
| ieu-a-1128 | ER- Breast | cg00178850 | Wald ratio | 1 | 0.066265 | 0.041907 | 0.113821 | -0.01587 | 0.148402 | 1.06851 | 0.984253 | 1.159979 |
| ieu-a-1128 | ER- Breast | cg00205605 | Wald ratio | 1 | 0.001403 | 0.018547 | 0.939713 | -0.03495 | 0.037756 | 1.001404 | 0.965654 | 1.038477 |
| ieu-a-1128 | ER- Breast | cg04019636 | Wald ratio | 1 | 0.016922 | 0.030936 | 0.584373 | -0.04371 | 0.077557 | 1.017066 | 0.957229 | 1.080644 |
| ieu-a-1128 | ER- Breast | cg19452802 | Wald ratio | 1 | -0.00336 | 0.035998 | 0.925729 | -0.07391 | 0.0672 | 0.99665 | 0.928754 | 1.069509 |
| ieu-a-1128 | ER- Breast | cg01937809 | Wald ratio | 1 | 0.024811 | 0.067734 | 0.714141 | -0.10795 | 0.157569 | 1.025121 | 0.897675 | 1.170661 |
| ieu-a-1128 | ER- Breast | cg00153942 | Wald ratio | 1 | -0.02866 | 0.032516 | 0.378125 | -0.09239 | 0.035073 | 0.971749 | 0.91175 | 1.035695 |
| ieu-a-1128 | ER- Breast | cg25064552 | Wald ratio | 1 | 0.008354 | 0.038057 | 0.826251 | -0.06624 | 0.082946 | 1.008389 | 0.935908 | 1.086483 |
| ieu-a-1128 | ER- Breast | cg01289343 | Wald ratio | 1 | -0.02196 | 0.023776 | 0.355628 | -0.06856 | 0.024638 | 0.978277 | 0.933734 | 1.024944 |
| ieu-a-1128 | ER- Breast | cg06522681 | Wald ratio | 1 | 0.011719 | 0.02753 | 0.670344 | -0.04224 | 0.065677 | 1.011788 | 0.95864 | 1.067882 |
| ieu-a-1128 | ER- Breast | cg11527913 | Wald ratio | 1 | 0.015995 | 0.039877 | 0.688347 | -0.06216 | 0.094154 | 1.016123 | 0.939728 | 1.098729 |
| ieu-a-1128 | ER- Breast | cg10585661 | Inverse vari | 2 | -0.01577 | 0.021524 | 0.463839 | -0.05796 | 0.02642 | 0.984356 | 0.943692 | 1.026772 |
| ieu-a-1128 | ER- Breast | cg01360605 | Wald ratio | 1 | 0.020725 | 0.059215 | 0.726339 | -0.09534 | 0.136788 | 1.020942 | 0.909067 | 1.146585 |
| ieu-a-1128 | ER- Breast | cg04180046 | Wald ratio | 1 | 0.014278 | 0.036461 | 0.695348 | -0.05719 | 0.085742 | 1.014381 | 0.944419 | 1.089525 |
| ieu-a-1128 | ER- Breast | cg11405655 | Wald ratio | 1 | 0.028767 | 0.036217 | 0.427029 | -0.04222 | 0.099752 | 1.029184 | 0.95866 | 1.104896 |
| ieu-a-1128 | ER- Breast | cg19935065 | Wald ratio | 1 | -0.01645 | 0.043075 | 0.702503 | -0.10088 | 0.067975 | 0.983682 | 0.904042 | 1.070338 |
| ieu-a-1128 | ER- Breast | cg06880612 | Wald ratio | 1 | -0.01882 | 0.056941 | 0.740963 | -0.13043 | 0.092781 | 0.981353 | 0.877719 | 1.097222 |
| ieu-a-1128 | ER- Breast | cg07383757 | Wald ratio | 1 | 0.026679 | 0.057554 | 0.642976 | -0.08613 | 0.139484 | 1.027038 | 0.917478 | 1.149681 |
| ieu-a-1128 | ER- Breast | cg24718197 | Wald ratio | 1 | -0.05269 | 0.038717 | 0.173531 | -0.12858 | 0.023194 | 0.948672 | 0.879346 | 1.023465 |
| ieu-a-1128 | ER- Breast | cg26038582 | Wald ratio | 1 | -0.00925 | 0.017149 | 0.589797 | -0.04286 | 0.024366 | 0.990797 | 0.958048 | 1.024666 |
| ieu-a-1128 | ER- Breast | cg06459104 | Inverse vari | 2 | 0.003999 | 0.01653 | 0.808835 | -0.0284 | 0.036398 | 1.004007 | 0.972 | 1.037069 |
| ieu-a-1128 | ER- Breast | cg00835193 | Wald ratio | 1 | -0.02266 | 0.033082 | 0.493319 | -0.0875 | 0.042178 | 0.977593 | 0.916217 | 1.04308 |
| ieu-a-1128 | ER- Breast | cg07202214 | Wald ratio | 1 | 0.017324 | 0.042757 | 0.685351 | -0.06648 | 0.101128 | 1.017475 | 0.935682 | 1.106418 |
| ieu-a-1128 | ER- Breast | cg14120896 | Wald ratio | 1 | -0.02862 | 0.042717 | 0.502858 | -0.11235 | 0.055105 | 0.971785 | 0.893736 | 1.056651 |
| ieu-a-1128 | ER- Breast | cg19758448 | Wald ratio | 1 | -0.03568 | 0.021477 | 0.096682 | -0.07777 | 0.006418 | 0.964953 | 0.925177 | 1.006439 |
| ieu-a-1128 | ER- Breast | cg26055950 | Wald ratio | 1 | -0.03973 | 0.032812 | 0.225919 | -0.10405 | 0.024578 | 0.961046 | 0.901185 | 1.024883 |
| ieu-a-1128 | ER- Breast | cg23126342 | Wald ratio | 1 | -0.01952 | 0.042512 | 0.646048 | -0.10285 | 0.063799 | 0.980665 | 0.902265 | 1.065878 |
| ieu-a-1128 | ER- Breast | cg24539517 | Wald ratio | 1 | -0.00317 | 0.022571 | 0.888166 | -0.04741 | 0.041065 | 0.996831 | 0.953693 | 1.04192 |
| ieu-a-1128 | ER- Breast | cg02629070 | Wald ratio | 1 | -0.03487 | 0.038299 | 0.362641 | -0.10993 | 0.040201 | 0.965735 | 0.895896 | 1.04102 |
| ieu-a-1128 | ER- Breast | cg15626881 | Wald ratio | 1 | -0.06041 | 0.043852 | 0.168336 | -0.14636 | 0.025541 | 0.941379 | 0.863846 | 1.02587 |
| ieu-a-1128 | ER- Breast | cg09099830 | Wald ratio | 1 | 0.073434 | 0.037643 | 0.051079 | -0.00035 | 0.147214 | 1.076198 | 0.999654 | 1.158602 |
| ieu-a-1128 | ER- Breast | cg12616487 | Wald ratio | 1 | -0.13815 | 0.047037 | 0.003314 | -0.23034 | -0.04596 | 0.87097 | 0.794263 | 0.955084 |
| ieu-a-1128 | ER- Breast | cg14174221 | Wald ratio | 1 | 0.01402 | 0.026944 | 0.602837 | -0.03879 | 0.06683 | 1.014118 | 0.961952 | 1.069114 |
| ieu-a-1128 | ER- Breast | cg03970900 | Wald ratio | 1 | 0.012439 | 0.026565 | 0.639603 | -0.03963 | 0.064508 | 1.012517 | 0.961146 | 1.066634 |

| ieu-a-1128 | ER- Breast | cg15903032 | Wald ratio | 1 | -0.01926 | 0.041048 | 0.638905 | -0.09972 | 0.061194 | 0.980923 | 0.905095 | 1.063105 |
| --- | --- | --- | --- | --- | --- | --- | --- | --- | --- | --- | --- | --- |
| ieu-a-1128 | ER- Breast | cg01020987 | Wald ratio | 1 | -0.01891 | 0.028362 | 0.504985 | -0.0745 | 0.036681 | 0.98127 | 0.92821 | 1.037363 |
| ieu-a-1128 | ER- Breast | cg06951627 | Wald ratio | 1 | 0.011725 | 0.038765 | 0.762294 | -0.06425 | 0.087705 | 1.011794 | 0.937766 | 1.091666 |
| ieu-a-1128 | ER- Breast | cg02032558 | Wald ratio | 1 | 0.024292 | 0.034703 | 0.483927 | -0.04373 | 0.092311 | 1.02459 | 0.957216 | 1.096706 |
| ieu-a-1128 | ER- Breast | cg04337534 | Wald ratio | 1 | -0.11014 | 0.052777 | 0.036892 | -0.21358 | -0.0067 | 0.895707 | 0.807684 | 0.993322 |
| ieu-a-1128 | ER- Breast | cg15474579 | Wald ratio | 1 | -0.06738 | 0.032907 | 0.040597 | -0.13188 | -0.00288 | 0.934839 | 0.876448 | 0.997121 |
| ieu-a-1128 | ER- Breast | cg06382664 | Wald ratio | 1 | 0.0037 | 0.034287 | 0.914064 | -0.0635 | 0.070903 | 1.003707 | 0.938472 | 1.073477 |
| ieu-a-1128 | ER- Breast | cg13561409 | Wald ratio | 1 | 0.046124 | 0.037431 | 0.217853 | -0.02724 | 0.119488 | 1.047204 | 0.973128 | 1.12692 |
| ieu-a-1128 | ER- Breast | cg09935388 | Wald ratio | 1 | 0.015845 | 0.060427 | 0.79315 | -0.10259 | 0.134282 | 1.015972 | 0.902495 | 1.143716 |
| ieu-a-1128 | ER- Breast | cg15084803 | Wald ratio | 1 | 0.008214 | 0.024642 | 0.738883 | -0.04008 | 0.056513 | 1.008248 | 0.960708 | 1.058141 |
| ieu-a-1128 | ER- Breast | cg05886626 | Wald ratio | 1 | -0.07192 | 0.037671 | 0.05625 | -0.14575 | 0.001918 | 0.930607 | 0.864371 | 1.00192 |
| ieu-a-1128 | ER- Breast | cg17390562 | Wald ratio | 1 | -0.00294 | 0.033265 | 0.929483 | -0.06814 | 0.062255 | 0.997061 | 0.934127 | 1.064234 |
| ieu-a-1128 | ER- Breast | cg16646054 | Wald ratio | 1 | -0.01221 | 0.032204 | 0.704553 | -0.07533 | 0.050909 | 0.987863 | 0.927436 | 1.052227 |
| ieu-a-1128 | ER- Breast | cg21187770 | Wald ratio | 1 | -0.03194 | 0.029929 | 0.285852 | -0.0906 | 0.026719 | 0.968563 | 0.913381 | 1.027079 |
| ieu-a-1128 | ER- Breast | cg08458637 | Wald ratio | 1 | -0.03238 | 0.03886 | 0.404657 | -0.10855 | 0.043782 | 0.968135 | 0.897135 | 1.044755 |
| ieu-a-1128 | ER- Breast | cg00498211 | Inverse vari | 2 | -0.01065 | 0.021354 | 0.617922 | -0.0525 | 0.031202 | 0.989405 | 0.94885 | 1.031694 |
| ieu-a-1128 | ER- Breast | cg00574379 | Wald ratio | 1 | -0.0231 | 0.020673 | 0.263891 | -0.06362 | 0.017422 | 0.977168 | 0.938366 | 1.017575 |
| ieu-a-1128 | ER- Breast | cg23771366 | Wald ratio | 1 | 0.019132 | 0.039123 | 0.624834 | -0.05755 | 0.095813 | 1.019316 | 0.944075 | 1.100553 |
| ieu-a-1128 | ER- Breast | cg04263702 | Inverse vari | 2 | 0.01014 | 0.020338 | 0.618073 | -0.02972 | 0.050003 | 1.010192 | 0.970715 | 1.051275 |
| ieu-a-1128 | ER- Breast | cg20375836 | Wald ratio | 1 | 0.003142 | 0.039271 | 0.936237 | -0.07383 | 0.080113 | 1.003147 | 0.92883 | 1.08341 |
| ieu-a-1128 | ER- Breast | cg18146737 | Wald ratio | 1 | 0.012744 | 0.048601 | 0.79315 | -0.08251 | 0.108003 | 1.012826 | 0.920798 | 1.114051 |
| ieu-a-1128 | ER- Breast | cg18533225 | Wald ratio | 1 | -0.00361 | 0.025262 | 0.886403 | -0.05312 | 0.045905 | 0.996398 | 0.948264 | 1.046975 |
| ieu-a-1128 | ER- Breast | cg14016875 | Wald ratio | 1 | 0.011957 | 0.030558 | 0.695572 | -0.04794 | 0.071851 | 1.012029 | 0.953195 | 1.074495 |
| ieu-a-1128 | ER- Breast | cg11066209 | Wald ratio | 1 | -0.04642 | 0.027992 | 0.09725 | -0.10128 | 0.008444 | 0.954641 | 0.903677 | 1.00848 |
| ieu-a-1128 | ER- Breast | cg01185345 | Wald ratio | 1 | 0.029336 | 0.033326 | 0.378707 | -0.03598 | 0.094654 | 1.02977 | 0.964657 | 1.099278 |
| ieu-a-1128 | ER- Breast | cg24503796 | Wald ratio | 1 | 0.024846 | 0.026825 | 0.354327 | -0.02773 | 0.077423 | 1.025157 | 0.97265 | 1.080499 |
| ieu-a-1128 | ER- Breast | cg10835286 | Wald ratio | 1 | 0.024904 | 0.01764 | 0.158019 | -0.00967 | 0.059478 | 1.025216 | 0.990376 | 1.061283 |
| ieu-a-1128 | ER- Breast | cg07572233 | Wald ratio | 1 | -0.05701 | 0.039209 | 0.145911 | -0.13386 | 0.019835 | 0.94458 | 0.87471 | 1.020033 |
| ieu-a-1128 | ER- Breast | cg07278634 | Wald ratio | 1 | -0.05359 | 0.033626 | 0.110992 | -0.1195 | 0.012315 | 0.94782 | 0.887366 | 1.012392 |
| ieu-a-1128 | ER- Breast | cg01651915 | Wald ratio | 1 | 0.011166 | 0.040012 | 0.780191 | -0.06726 | 0.089591 | 1.011229 | 0.934954 | 1.093726 |
| ieu-a-1128 | ER- Breast | cg12619504 | Wald ratio | 1 | 0.009401 | 0.035864 | 0.793217 | -0.06089 | 0.079694 | 1.009445 | 0.940925 | 1.082955 |
| ieu-a-1128 | ER- Breast | cg09639152 | Wald ratio | 1 | 0.057929 | 0.027516 | 0.035268 | 0.003997 | 0.111861 | 1.05964 | 1.004005 | 1.118357 |
| ieu-a-1128 | ER- Breast | cg08479476 | Wald ratio | 1 | 0.060556 | 0.027387 | 0.027025 | 0.006878 | 0.114235 | 1.062428 | 1.006902 | 1.121015 |
| ieu-a-1128 | ER- Breast | cg14550518 | Wald ratio | 1 | 0.025435 | 0.037483 | 0.497409 | -0.04803 | 0.098902 | 1.025761 | 0.953103 | 1.103958 |
| ieu-a-1128 | ER- Breast | cg21698310 | Wald ratio | 1 | -0.00664 | 0.039494 | 0.866583 | -0.08404 | 0.070774 | 0.993387 | 0.919391 | 1.073339 |
| ieu-a-1128 | ER- Breast | cg01294327 | Wald ratio | 1 | 0.001864 | 0.01553 | 0.904483 | -0.02857 | 0.032302 | 1.001865 | 0.971829 | 1.03283 |
| ieu-a-1128 | ER- Breast | cg01498900 | Wald ratio | 1 | -0.03717 | 0.026817 | 0.165758 | -0.08973 | 0.015394 | 0.963515 | 0.914178 | 1.015513 |
| ieu-a-1128 | ER- Breast | cg17586094 | Wald ratio | 1 | -0.01455 | 0.02689 | 0.588519 | -0.06725 | 0.038157 | 0.985558 | 0.93496 | 1.038895 |

| ieu-a-1128 | ER- Breast | cg06478823 | Wald ratio | 1 | 0.039028 | 0.022265 | 0.079624 | -0.00461 | 0.082667 | 1.039799 | 0.995399 | 1.08618 |
| --- | --- | --- | --- | --- | --- | --- | --- | --- | --- | --- | --- | --- |
| ieu-a-1128 | ER- Breast | cg14975410 | Wald ratio | 1 | 0.025127 | 0.032624 | 0.441189 | -0.03882 | 0.08907 | 1.025445 | 0.961927 | 1.093157 |
| ieu-a-1128 | ER- Breast | cg05599106 | Wald ratio | 1 | 0.011681 | 0.033895 | 0.73037 | -0.05475 | 0.078116 | 1.01175 | 0.946719 | 1.081248 |
| ieu-a-1128 | ER- Breast | cg04506190 | Wald ratio | 1 | -0.04422 | 0.039094 | 0.258041 | -0.12084 | 0.032408 | 0.956747 | 0.886175 | 1.032939 |
| ieu-a-1128 | ER- Breast | cg05214460 | Wald ratio | 1 | -0.03787 | 0.031561 | 0.230139 | -0.09973 | 0.023986 | 0.962835 | 0.90508 | 1.024276 |
| ieu-a-1128 | ER- Breast | cg21929761 | Wald ratio | 1 | 0.044817 | 0.035711 | 0.209474 | -0.02518 | 0.11481 | 1.045837 | 0.975139 | 1.121661 |
| ieu-a-1128 | ER- Breast | cg26703507 | Wald ratio | 1 | -0.0697 | 0.028079 | 0.013048 | -0.12474 | -0.01467 | 0.932669 | 0.882727 | 0.985437 |
| ieu-a-1128 | ER- Breast | cg10843276 | Wald ratio | 1 | 0.006341 | 0.022283 | 0.775987 | -0.03733 | 0.050014 | 1.006361 | 0.963355 | 1.051286 |
| ieu-a-1128 | ER- Breast | cg14194983 | Wald ratio | 1 | 0.011996 | 0.016519 | 0.467722 | -0.02038 | 0.044374 | 1.012068 | 0.979825 | 1.045373 |
| ieu-a-1128 | ER- Breast | cg24361098 | Wald ratio | 1 | -0.03852 | 0.026639 | 0.148134 | -0.09074 | 0.013689 | 0.962208 | 0.913257 | 1.013783 |
| ieu-a-1128 | ER- Breast | cg12828729 | Wald ratio | 1 | -0.0477 | 0.031442 | 0.129248 | -0.10933 | 0.013926 | 0.95342 | 0.896439 | 1.014024 |
| ieu-a-1128 | ER- Breast | cg02279625 | Wald ratio | 1 | -0.05249 | 0.034201 | 0.124847 | -0.11952 | 0.014544 | 0.948864 | 0.887342 | 1.014651 |
| ieu-a-1128 | ER- Breast | cg15693572 | Wald ratio | 1 | -0.01037 | 0.029192 | 0.722311 | -0.06759 | 0.046842 | 0.98968 | 0.934644 | 1.047956 |
| ieu-a-1128 | ER- Breast | cg16822035 | Wald ratio | 1 | -0.08182 | 0.049174 | 0.096126 | -0.1782 | 0.014559 | 0.921436 | 0.836773 | 1.014665 |
| ieu-a-1128 | ER- Breast | cg00995520 | Wald ratio | 1 | 0.049102 | 0.047904 | 0.305363 | -0.04479 | 0.142994 | 1.050327 | 0.956198 | 1.153723 |
| ieu-a-1128 | ER- Breast | cg03554335 | Wald ratio | 1 | 0.053659 | 0.042276 | 0.204359 | -0.0292 | 0.13652 | 1.055124 | 0.971219 | 1.146278 |
| ieu-a-1128 | ER- Breast | cg15339249 | Wald ratio | 1 | -0.02992 | 0.041949 | 0.4757 | -0.11214 | 0.052301 | 0.970523 | 0.893919 | 1.053693 |
| ieu-a-1128 | ER- Breast | cg18132076 | Wald ratio | 1 | -0.03161 | 0.031349 | 0.313261 | -0.09306 | 0.029831 | 0.968882 | 0.911143 | 1.030281 |
| ieu-a-1128 | ER- Breast | cg06968912 | Wald ratio | 1 | -0.03697 | 0.065078 | 0.569984 | -0.16452 | 0.090584 | 0.963706 | 0.848299 | 1.094813 |
| ieu-a-1128 | ER- Breast | cg09447622 | Wald ratio | 1 | -0.00545 | 0.041796 | 0.896222 | -0.08737 | 0.076469 | 0.994563 | 0.916335 | 1.079469 |
| ieu-a-1128 | ER- Breast | cg02186444 | Wald ratio | 1 | -0.02771 | 0.035672 | 0.437242 | -0.09763 | 0.042205 | 0.972668 | 0.906985 | 1.043108 |
| ieu-a-1128 | ER- Breast | cg14222656 | Wald ratio | 1 | -0.03478 | 0.032609 | 0.286122 | -0.0987 | 0.02913 | 0.965815 | 0.906018 | 1.029559 |
| ieu-a-1128 | ER- Breast | cg08867399 | Inverse vari | 3 | 0.022723 | 0.024009 | 0.343941 | -0.02434 | 0.069781 | 1.022983 | 0.975958 | 1.072273 |
| ieu-a-1128 | ER- Breast | cg13809441 | Wald ratio | 1 | 0.010923 | 0.024202 | 0.651754 | -0.03651 | 0.058359 | 1.010983 | 0.964145 | 1.060096 |
| ieu-a-1128 | ER- Breast | cg19804488 | Wald ratio | 1 | -0.04692 | 0.039881 | 0.239407 | -0.12508 | 0.031248 | 0.954165 | 0.882423 | 1.031741 |
| ieu-a-1128 | ER- Breast | cg00756943 | Inverse vari | 2 | 0.042221 | 0.028894 | 0.143952 | -0.01441 | 0.098853 | 1.043125 | 0.985692 | 1.103904 |
| ieu-a-1128 | ER- Breast | cg15955046 | Wald ratio | 1 | 0.03081 | 0.024259 | 0.204059 | -0.01674 | 0.078357 | 1.03129 | 0.983403 | 1.081509 |
| ieu-a-1128 | ER- Breast | cg08305533 | Wald ratio | 1 | 0.049667 | 0.042063 | 0.237686 | -0.03278 | 0.13211 | 1.050921 | 0.967756 | 1.141234 |
| ieu-a-1128 | ER- Breast | cg21791252 | Wald ratio | 1 | 0.062187 | 0.033051 | 0.059893 | -0.00259 | 0.126967 | 1.064162 | 0.997411 | 1.135379 |
| ieu-a-1128 | ER- Breast | cg07029024 | Inverse vari | 2 | -0.0284 | 0.033411 | 0.395247 | -0.09389 | 0.037082 | 0.971995 | 0.910383 | 1.037778 |
| ieu-a-1128 | ER- Breast | cg09611599 | Wald ratio | 1 | 0.020136 | 0.015718 | 0.200165 | -0.01067 | 0.050943 | 1.02034 | 0.989386 | 1.052263 |
| ieu-a-1128 | ER- Breast | cg08472795 | Wald ratio | 1 | -0.03261 | 0.033333 | 0.327868 | -0.09795 | 0.032719 | 0.967912 | 0.906697 | 1.033261 |
| ieu-a-1128 | ER- Breast | cg10696445 | Wald ratio | 1 | -0.06812 | 0.036348 | 0.060928 | -0.13936 | 0.003125 | 0.934151 | 0.869916 | 1.00313 |
| ieu-a-1128 | ER- Breast | cg04017131 | Inverse vari | 2 | -0.02348 | 0.054336 | 0.665696 | -0.12998 | 0.083022 | 0.976797 | 0.878117 | 1.086566 |
| ieu-a-1128 | ER- Breast | cg05661533 | Wald ratio | 1 | -0.10748 | 0.064978 | 0.09811 | -0.23483 | 0.019877 | 0.898096 | 0.790702 | 1.020076 |
| ieu-a-1128 | ER- Breast | cg13057898 | Wald ratio | 1 | 0.035692 | 0.022049 | 0.105511 | -0.00753 | 0.078909 | 1.036336 | 0.992503 | 1.082105 |
| ieu-a-1128 | ER- Breast | cg11105358 | Wald ratio | 1 | 0.010669 | 0.017874 | 0.550575 | -0.02436 | 0.045703 | 1.010726 | 0.97593 | 1.046764 |
| ieu-a-1128 | ER- Breast | cg07751331 | Wald ratio | 1 | -0.02182 | 0.030626 | 0.476188 | -0.08185 | 0.038207 | 0.978417 | 0.921414 | 1.038946 |

| ieu-a-1128 | ER- Breast | cg04545963 | Wald ratio | 1 | 0.018448 | 0.02905 | 0.525405 | -0.03849 | 0.075386 | 1.018619 | 0.962241 | 1.0783 |
| --- | --- | --- | --- | --- | --- | --- | --- | --- | --- | --- | --- | --- |
| ieu-a-1128 | ER- Breast | cg23716141 | Inverse vari | 2 | 0.006278 | 0.022685 | 0.781979 | -0.03818 | 0.05074 | 1.006298 | 0.962535 | 1.052049 |
| ieu-a-1128 | ER- Breast | cg07207043 | Wald ratio | 1 | -0.02646 | 0.016107 | 0.100412 | -0.05803 | 0.005108 | 0.973885 | 0.943619 | 1.005121 |
| ieu-a-1128 | ER- Breast | cg24924577 | Wald ratio | 1 | -0.02972 | 0.036034 | 0.409452 | -0.10035 | 0.040904 | 0.970714 | 0.904521 | 1.041752 |
| ieu-a-1128 | ER- Breast | cg26126879 | Wald ratio | 1 | 0.027132 | 0.018375 | 0.139793 | -0.00888 | 0.063147 | 1.027503 | 0.991156 | 1.065183 |
| ieu-a-1128 | ER- Breast | cg05542681 | Wald ratio | 1 | 0.010475 | 0.021273 | 0.622419 | -0.03122 | 0.052171 | 1.01053 | 0.969262 | 1.053556 |
| ieu-a-1128 | ER- Breast | cg09307264 | Wald ratio | 1 | -0.0519 | 0.026958 | 0.054183 | -0.10474 | 0.000933 | 0.94942 | 0.900556 | 1.000934 |
| ieu-a-1128 | ER- Breast | cg12806681 | Wald ratio | 1 | 0.017796 | 0.034959 | 0.610721 | -0.05072 | 0.086316 | 1.017955 | 0.950541 | 1.090151 |
| ieu-a-1128 | ER- Breast | cg14880079 | Wald ratio | 1 | -0.02501 | 0.055181 | 0.650378 | -0.13316 | 0.083144 | 0.9753 | 0.875322 | 1.086698 |
| ieu-a-1128 | ER- Breast | cg05183538 | Wald ratio | 1 | -0.14358 | 0.063136 | 0.022954 | -0.26733 | -0.01984 | 0.866248 | 0.765419 | 0.980358 |
| ieu-a-1128 | ER- Breast | cg25189904 | Wald ratio | 1 | 0.026048 | 0.033126 | 0.431677 | -0.03888 | 0.090974 | 1.02639 | 0.961867 | 1.09524 |
| ieu-a-1128 | ER- Breast | cg22158051 | Wald ratio | 1 | 0.016272 | 0.02654 | 0.539813 | -0.03575 | 0.068291 | 1.016405 | 0.964884 | 1.070676 |
| ieu-a-1128 | ER- Breast | cg26102435 | Wald ratio | 1 | -0.02329 | 0.030208 | 0.440806 | -0.08249 | 0.035922 | 0.976984 | 0.920819 | 1.036575 |
| ieu-a-1128 | ER- Breast | cg11014740 | Wald ratio | 1 | 0.003261 | 0.044022 | 0.940951 | -0.08302 | 0.089543 | 1.003266 | 0.920331 | 1.093675 |
| ieu-a-1128 | ER- Breast | cg04666465 | Wald ratio | 1 | -0.01255 | 0.015976 | 0.432035 | -0.04387 | 0.018761 | 0.987526 | 0.957082 | 1.018938 |
| ieu-a-1128 | ER- Breast | cg01471372 | Inverse vari | 2 | -0.02279 | 0.030996 | 0.462272 | -0.08354 | 0.037967 | 0.977472 | 0.919856 | 1.038697 |
| ieu-a-1128 | ER- Breast | cg08712631 | Wald ratio | 1 | -0.06362 | 0.038895 | 0.101877 | -0.13986 | 0.012609 | 0.938357 | 0.869481 | 1.012689 |
| ieu-a-1128 | ER- Breast | cg11095027 | Wald ratio | 1 | 0.015253 | 0.030506 | 0.617075 | -0.04454 | 0.075044 | 1.01537 | 0.956439 | 1.077931 |
| ieu-a-1128 | ER- Breast | cg03710029 | Wald ratio | 1 | -0.02897 | 0.026694 | 0.277875 | -0.08129 | 0.023355 | 0.971449 | 0.921929 | 1.02363 |
