# Supplementary material for: Smoking, DNA Methylation, and Breast Cancer: A Mendelian Randomization Study
Source: Front Oncol. 2021 Sep 28;11:745918. doi: 10.3389/fonc.2021.745918 (PMC8507148; doi:10.3389/fonc.2021.745918)
Supplement: Supplementary file 5 [file Table_4.docx]

Table S4. Phenome-wide associations of the four top findings in MR analysis.

| snp | chr | a1 | a2 | trait | efo | study | ancestry | year | beta | se | p | n |
| --- | --- | --- | --- | --- | --- | --- | --- | --- | --- | --- | --- | --- |
| 1 rs8035987 | 15 | C | T | Arm predicted mass left | - | Neale B | European | 2017 | 0.008038 | 0.00178 | 6.30E-06 | 331146 |
| 2 rs8035987 | 15 | C | T | Forced vital capacity | EFO_0004312 | Neale B | European | 2017 | 0.01231 | 0.002281 | 6.88E-08 | 307638 |
| 3 rs8035987 | 15 | C | T | Forced vital capacity | EFO_0004312 | Neale B | European | 2017 | 0.01307 | 0.002504 | 1.81E-07 | 255492 |
| 4 rs8035987 | 15 | C | T | Hand grip strength right | EFO_0006941 | Neale B | European | 2017 | 0.009364 | 0.00203 | 3.98E-06 | 335842 |
| 5 rs8035987 | 15 | C | T | Height | EFO_0004339 | Neale B | European | 2017 | 0.01746 | 0.001997 | 2.29E-18 | 336474 |
| 6 rs8035987 | 15 | C | T | Sitting height | EFO_0004339 | Neale B | European | 2017 | 0.01646 | 0.002172 | 3.47E-14 | 336172 |
| 7 rs8035987 | 15 | C | T | Trunk fat-free mass | - | Neale B | European | 2017 | 0.009497 | 0.00177 | 8.08E-08 | 331030 |
| 8 rs8035987 | 15 | C | T | Trunk predicted mass | - | Neale B | European | 2017 | 0.009446 | 0.001764 | 8.61E-08 | 330995 |
| 9 rs8035987 | 15 | C | T | Whole body fat-free mass | - | Neale B | European | 2017 | 0.007864 | 0.001777 | 9.62E-06 | 331291 |
| 10 rs8035987 | 15 | C | T | Whole body water mass | - | Neale B | European | 2017 | 0.008121 | 0.00178 | 5.04E-06 | 331315 |
| 11 rs4986117 | 17 | G | T | HbA1C | EFO_0004541 | MAGIC | European | 2010 | -0.0171 | 0.0038 | 8.73E-06 | 46368 |
| 12 rs4986117 | 17 | G | T | HbA1c | EFO_0004541 | MAGIC | European | 2017 | -0.01 | 0.002 | 3.72E-07 | 123665 |
| 13 rs8067926 | 17 | A | G | HbA1c | EFO_0004541 | MAGIC | European | 2017 | -0.011 | 0.0018 | 5.94E-10 | 123665 |
| 14 rs10852865 | 17 | A | C | Platelet distribution width | EFO_0004586 | Astle W | European | 2016 | -0.02541 | 0.00483 | 1.44E-07 | 173480 |
| 15 rs4411372 | 13 | C | T | Years of education | NCIT_C122393 | SSGAC | European | 2013 | NA | NA | 9.85E-06 | 101069 |
| 16 rs4411372 | 13 | C | T | Arm fat mass left | - | Neale B | European | 2017 | -0.01326 | 0.002649 | 5.61E-07 | 331164 |
| 17 rs4411372 | 13 | C | T | Arm fat mass right | - | Neale B | European | 2017 | -0.01339 | 0.002648 | 4.30E-07 | 331226 |
| 18 rs4411372 | 13 | C | T | Arm fat percentage left | - | Neale B | European | 2017 | -0.01272 | 0.002046 | 5.06E-10 | 331198 |
| 19 rs4411372 | 13 | C | T | Arm fat percentage right | - | Neale B | European | 2017 | -0.01269 | 0.002055 | 6.73E-10 | 331249 |
| 20 rs4411372 | 13 | C | T | Body fat percentage | EFO_0007800 | Neale B | European | 2017 | -0.01405 | 0.002072 | 1.20E-11 | 331117 |
| 21 rs4411372 | 13 | C | T | Body mass index | EFO_0004340 | Neale B | European | 2017 | -0.01336 | 0.002674 | 5.86E-07 | 336107 |
| 22 rs4411372 | 13 | C | T | Forced vital capacity | EFO_0004312 | Neale B | European | 2017 | 0.01016 | 0.002194 | 3.69E-06 | 307638 |
| 23 rs4411372 | 13 | C | T | Forced vital capacity | EFO_0004312 | Neale B | European | 2017 | 0.01122 | 0.002408 | 3.17E-06 | 255492 |
| 24 rs4411372 | 13 | C | T | Leg fat mass left | - | Neale B | European | 2017 | -0.01121 | 0.002136 | 1.55E-07 | 331275 |
| 25 rs4411372 | 13 | C | T | Leg fat mass right | - | Neale B | European | 2017 | -0.01218 | 0.002161 | 1.74E-08 | 331293 |
| 26 rs4411372 | 13 | C | T | Leg fat percentage left | - | Neale B | European | 2017 | -0.01072 | 0.00169 | 2.22E-10 | 331278 |
| 27 rs4411372 | 13 | C | T | Leg fat percentage right | - | Neale B | European | 2017 | -0.01167 | 0.001712 | 9.15E-12 | 331296 |
| 28 rs4411372 | 13 | C | T | college or university degree | EFO_0004784 | Neale B | European | 2017 | 0.006089 | 0.001267 | 1.55E-06 | 334070 |
| 29 rs4411372 | 13 | C | T | Sodium in urine | EFO_0009282 | Neale B | European | 2017 | -0.01301 | 0.002631 | 7.69E-07 | 326831 |
| 30 rs4411372 | 13 | C | T | Trunk fat mass | EFO_0005409 | Neale B | European | 2017 | -0.01515 | 0.002721 | 2.59E-08 | 331093 |
| 31 rs4411372 | 13 | C | T | Trunk fat percentage | - | Neale B | European | 2017 | -0.01598 | 0.002484 | 1.24E-10 | 331113 |
| 32 rs4411372 | 13 | C | T | strenuous sports | EFO_0008002 | Neale B | European | 2017 | 0.003723 | 0.000822 | 5.91E-06 | 335599 |
| 33 rs4411372 | 13 | C | T | Waist circumference | EFO_0004342 | Neale B | European | 2017 | -0.0109 | 0.002394 | 5.26E-06 | 336639 |
| 34 rs4411372 | 13 | C | T | Whole body fat mass | - | Neale B | European | 2017 | -0.01493 | 0.002637 | 1.49E-08 | 330762 |
| 35 rs4411372 | 13 | C | T | Years of educational attainment | EFO_0004784 | SSGAC | European | 2013 | 0.019 | 0.004 | 9.85E-06 | 101069 |
| 36 rs4411372 | 13 | C | T | Years of educational attainment | EFO_0004784 | SSGAC | European | 2016 | 0.014 | 0.003 | 2.93E-07 | 328917 |
